# The Dependency of Nematic and Twist-bend Mesophase Formation on Bend Angle

**DOI:** 10.1038/srep36682

**Published:** 2016-11-07

**Authors:** Richard J. Mandle, Craig T. Archbold, Julia P. Sarju, Jessica L. Andrews, John W. Goodby

**Affiliations:** 1 Department of Chemistry, University of York, YO10 5DD, UK

## Abstract

We have prepared and studied a family of cyanobiphenyl dimers with varying linking groups with a view to exploring how molecular structure dictates the stability of the nematic and twist-bend nematic mesophases. Using molecular modelling and 1D ^1^H NOESY NMR spectroscopy, we determine the angle between the two aromatic core units for each dimer and find a strong dependency of the stability of both the nematic and twist-bend mesophases upon this angle, thereby satisfying earlier theoretical models.

The discovery of a lower temperature nematic-like phase in bimesogens^1^, the phase later being identified as the ‘twist-bend nematic phase’ (N_TB_)^2^,^3^,^4^,^5^,^6^,^7^,^8^,^9^,^10^,^11^,^12^, has motivated the synthesis of a large number of novel compounds in an effort to understand what molecular features give rise to this unique mesophase^13^,^14^,^15^,^16^,^17^,^18^,^19^,^20^,^21^,^22^,^23^,^24^,^25^,^26^. Despite a great deal of research activity on the N_TB_ phase, a consensus concerning the local structure remains elusive, and consequently alternate models to the ‘twist-bend nematic’ have been proposed^27^,^28^,^29^. Around 100 compounds are known to exhibit the N_TB_ phase, and yet no apparent structure property relationship exists, except that the materials must exhibit a ‘bent’ gross molecular shape.

This study is motivated by a comparison of the mesophase properties of compounds **1** and **2**, shown in Fig. 1, in which the thermal stabilities of the nematic and twist-bend nematic phases of **2** are significantly higher than those of **1**. Compounds **1** (CB9CB) and **2** (CBI-5-ICB) are very closely related in chemical structure, with a proportion of the ‘linking’ dimethylene units of **1** replaced by imine (Schiff’s base) groups in **2**. Compared to a dimethylene unit an imine is more polar, exhibits electronic conjugation to the aromatic core and is less flexible. An imine adjacent to an aromatic ring has a C-N-C bond angle of 120° compared to 109.5° for the C-C-C bond in dimethylene unit bonded to an aromatic ring. We hypothesised that the stabilisation of both the N and N_TB_ phases may be due to one or more of these factors which will be manifested as changes in the flexibility of the spacer, the inter-aromatic angle, and the polarity of the individual mesogenic units. With this in mind we sought to prepare a range of cyanobiphenyl dimers with varying linking groups, allowing us to relate changes in the relative thermal stability of both mesophases to discrete changes in molecular structure. All materials were prepared so that the linking groups plus spacer length are equivalent in length and parity to the nonamethylene unit employed in CB9CB.

## Experimental

All reagents were purchased from commercial suppliers and used without further purification. Solvents were purchased from Fisher Scientific UK and dried by percolation over activated alumina prior to use. Compound **3** (CBO7OCB), was prepared *via* the alkylation of 4-hydroxy-4′-cyanobiphenyl with 1,7-dibromoheptane according to the procedure of Elmsley *et. al*.^30^ Quantum chemical calculations were performed in Gaussian G09 revision E.01^31^ on the York Advanced Research Computing Cluster (YARCC) as described in the text, with molecular structures rendered from output files using QuteMol^32^. Calculations were performed using the B3LYP functional and the 6-31G(d) basis set on isolated molecules at a temperature of 298 K. Small angle X-ray scattering was performed on a Bruker D8 Discover equipped with a temperature controlled, bored graphite rod furnace, custom built at the University of York. Full details of experimental procedures and instrumentation used, including full chemical characterisation data for all compounds, is given in the accompanying electronic supplementary information to this article. Raw data are available upon request from the University of York data catalogue.

## Results and Discussion

**Figure 2:**
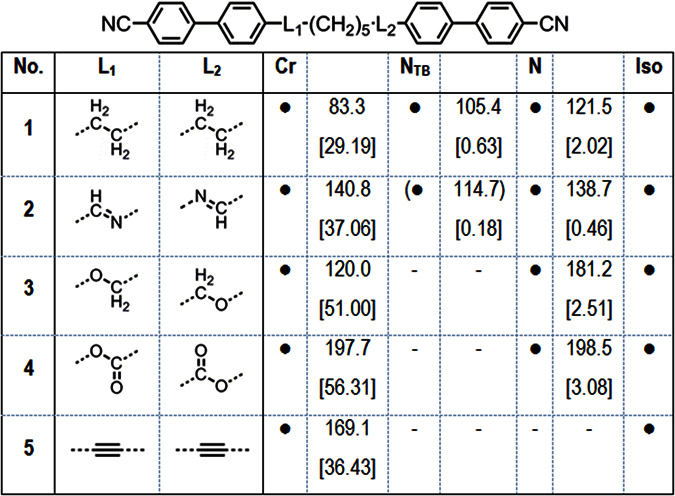
Transition temperatures (°C) and associated enthalpies of transition [kJ mol-1] for compounds **1–5**. Transitions in parenthesis () are monotropic, i.e. they occur below the melting point of the sample.

All final materials were subject to characterisation of their liquid crystal behaviour by a combination of polarised optical microscopy (POM) and differential scanning calorimetry (DSC), assisted by small angle X-ray scattering (SAXS). The structures, transition temperatures, and associated enthalpies of transition for compounds **1–5** are given in Fig. 2.

**Figure 3:**
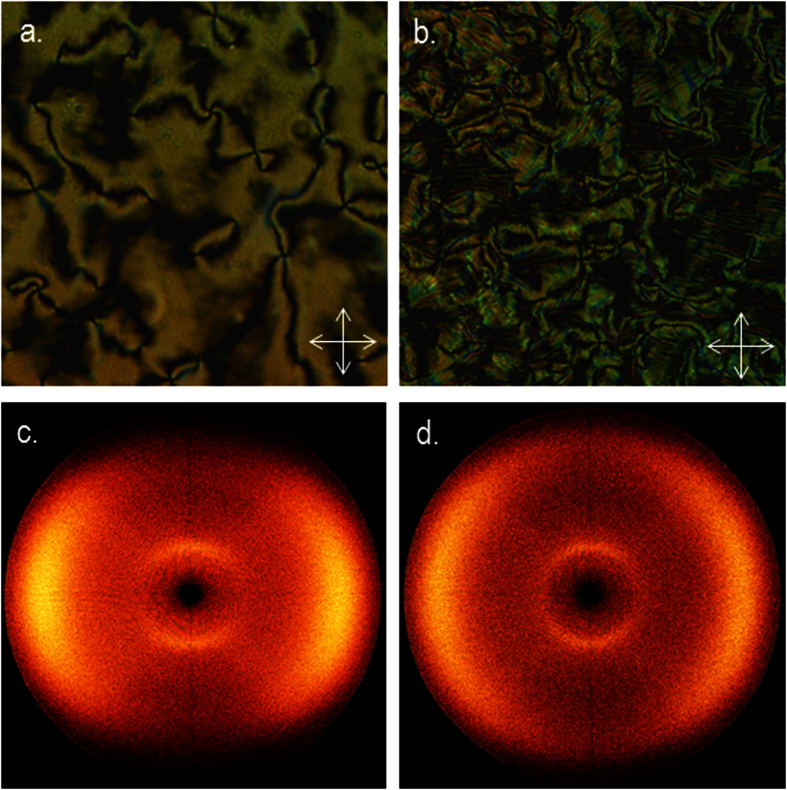
Photomicrographs (x100) of the nematic phase of compound **2** (**a**, 125.5 °C) and the N_TB_ phase of compound **2** (**b**, 107 °C) along with small angle X-ray scattering patterns of a magnetically aligned sample of compound **2** in the nematic phase (**c**, 120 °C) and the partially aligned N_TB_ phase (**d**, 110 °C).

In the case of compound **2** the nematic and twist-bend nematic phases of compound were initially each identified from their distinctive optical textures, with representative photomicrographs shown in Fig. 3. Small angle X-ray scattering of compound **2** confirmed that both mesophases lack lamellar order with the lower temperature mesophase confirmed unequivocally as the N_TB_ by miscibility studies with CB9CB; the phase diagram showing complete miscibility at all concentrations (phase diagrams are presented in the ESI). It is perhaps unsurprising that **2** exhibits nematic and N_TB_ mesophases given that closely related homologues also exhibit these phases^20^. The transition temperatures measured by us for compounds **1** (CB9CB), **2, 3** and **4** are in good agreement with literature values^6^,^27^,^30^,^33^. Compounds **3** (CBO7OCB) and **4**, with ether and ester linking groups respectively, do not exhibit the twist-bend nematic phase in their neat state, however both compounds exhibit enantiotropic nematic phases^33^,^34^. The diyne **5** was found to be non-mesogenic; the material could be supercooled to around 135 °C at which point recrystalisation of the sample occurred. The dramatic increase in melting point of compound **5** compared to the parent compound **1**, as well as the lack of mesomorphic behaviour in **5**, is a consequence of both the reduced flexibility and increased π-π intermolecular interactions that result from the material having two alkyne linking groups.

**Figure 4:**
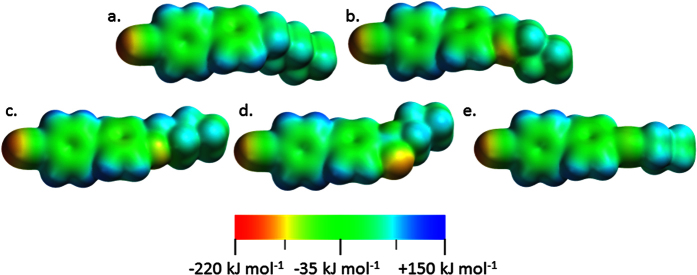
Electrostatic potential isosurfaces for one half fragments of (**a**, L = -CH_2_-CH_2_-) compound **1**, (**b**, L = -C(H) = N-) compound **2**, (**c**, L = -O-CH_2_-) compound **3**, **(d** L = -OC(O)-) compound **4** and (**e**, L = -C≡C-) compound **5**, as calculated at the B3LYP/6-31G(d) level of theory on geometries optimised at the same level of theory. Values range from – 220 kJ mol^−1^ (orange/red) to +150 kJ mol^−1^ (blue/purple).

**Figure 5:**
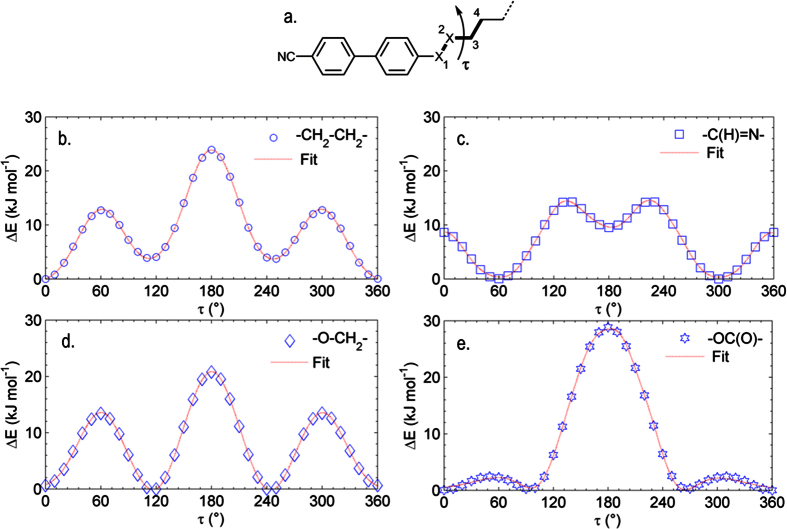
The dihedral angle τ between the atoms numbered 1 and 4 is defined as shown in (**a**). Plots of rotation about this dihedral at the B3LYP/6-31G(d) level of theory (36 × 10° steps) where ‘X’ is equal to a dimethylene unit (**b**, -CH_2_-CH_2_-), an imine (**c**, -C(H) = N-), an ether (**d**, -O-CH_2_-) and a carboxylate ester (**e**, -C(O)O-) are given along with 6-term Gaussian fits. Energies are given in kJ mol^−1^ and are relative to the lowest energy conformer.

Each of the linking groups employed in Fig. 2 impart differing steric and electronic properties which will now be considered in detail. Electrostatic potential (ESP) isosurfaces were calculated for one half of each symmetrical mesogen at the B3LYP/6-31G(d) level of theory, with values being in the region of approximately -200 kJ mol^−1^ to + 125 kJ mol^−1^ for all five systems studied. As expected the electrostatic potential across the biphenyl cores are similar, whereas significant differences are immediately apparent around the linking groups. Both compounds **1** (Fig. 4a) and **5** (Fig. 4e) have ‘non-polar’ linking groups and this is apparent from calculated ESP isosurfaces, therefore the significantly different mesomorphic behaviour exhibited by these two compounds suggests that it is steric rather than electronic factors that dictate the properties of each material in this instance. For example, compare compound **1**, with a ‘non polar’ linking group, with compound **2**, with a polar linking group; both exhibit the twist-bend nematic phase despite the different polarities associated with the linking units. It appears that the calculated electrostatic potentials are not particularly diagnostic for mesophase formation in this instance.

Turning now to steric factors, and taking inspiration from the work of Ivšić *et al*.^20^, we opted to probe the influence of the linking group by studying the dihedral torsional potentials. The alkyne **5** is non mesogenic and hence we opted not to investigate this computationally. The MODREDUNDANT keyword in Gaussian G09 allows, following completion of a calculation such as an optimization, the internal coordinates to be modified and the calculation repeated any number of times. In this work we use the MODREDUNDANT keyword to obtain optimised geometries at the B3LYP/6-31G(d) level of DFT where the dihedral angle between atoms 1–4 (Fig. 4a) was increased in 36 steps of 10°. The resulting data could be fitted satisfactorily using a 6 term Gaussian function. Plots of relative energy (ΔE, kJ mol^−1^) vs dihedral angle (τ,°) are given in Fig. 5.

**Figure 6:**
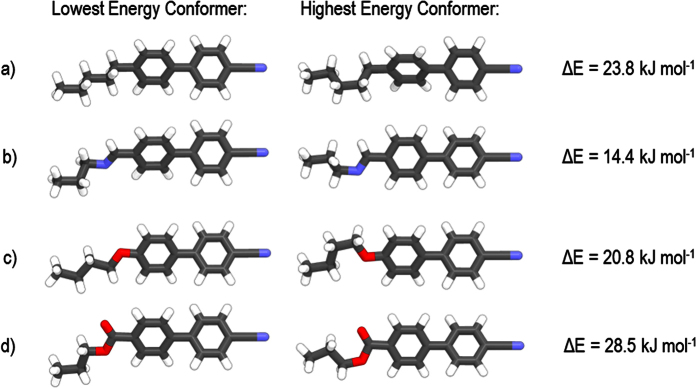
Lowest and highest energy conformations obtained at the B3LYP/6-31G(d) level of theory as described in the text for representative fragments of compounds **1** (**a**), **2** (**b**), **3** (**c**) and **4** (**d**). Colour coding refers to the element type in question (white = H, grey = C, blue = N, red = O).

For the methylene linked compound **1** (Fig. 5b) local minima exist (ΔE = +3.5 kJ mol^−1^) at ± 120° to the all *trans* global minima. The conformational landscape of **1** contrasts with that of the imine linked compound **2**, which exhibits its global minima at ≈ ±60° and a local energy minima at ≈180° (ΔE = 8.6 kJ mol^−1^). The ±60° states are separated by an energy barrier of ≈8.6 kJ mol^−1^ and are degenerate, giving two possible rotamers which are expected to be equally populated in the bulk, as described previously for the closely related dimer CBI-7-ICB^20^. For the ether linked compound **3** the single *gauche* conformer (±120°) lower in energy than the all *trans* conformer by 0.2 kJ mol^−1^. Compound **4** (Fig. 5e) exhibits local minima at ≈ ±90° which are similar in energy (ΔE = +0.1 kJ mol^−1^) to the global minima and as such are likely to be significantly populated. Molecular structures corresponding to global energy maxima and minima are presented in Fig. 6.

**Figure 7:**
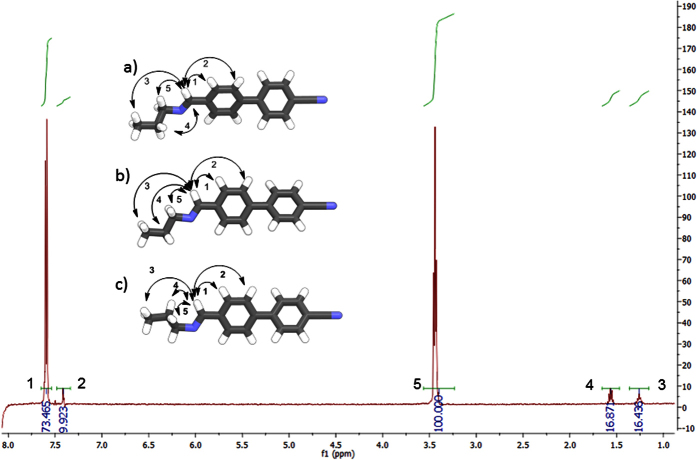
1D ^1^H NOESY spectra of compound **2** irradiated at 4058.17 Hz (8.153 ppm), which corresponds to the imine proton. Numbered black arrows indicate the assignment of observed NOE enhancements which are mapped onto the lowest energy geometry of **2** (**a**, τ ± 60), a local energy minimum of **2** (**b**, τ ± 0 and ± 180), and the energy maximum of **2** (**c**, τ ± 120).

We opted to study the conformational landscape of compounds **1–4** further using solution based 1D 1H NOESY (nuclear Overhauser Effect spectroscopy) NMR experiments in solution. For compound **1** and the homologous CB8CB crystal structures reveal that energetically unfavourable *gauche* conformers can exist in the solid state, demonstrating the need for experimental data to verify calculated geometries^35^. For flexible systems which exhibit a range of conformations in solutions, the observed NOE for each individual inter-proton distance for each conformer is averaged due to rapid interconversion on the NMR timescale^36^. A representative assigned 1D ^1^H NOE NMR spectra with assignments for compound **2** is given in Fig. 7; further NOE spectra for **2** and other compounds are given in the ESI to this article. In the case of compound **4** the flexible spacer only contains five methylene units, two of which are magnetically equivalent. The result is that for compound **4** ^1^H NOE difference experiments are not as useful as those performed on materials with multiple proton environments such as **1**, **2** and **3**.

Irradiation of the frequency that corresponds to the imine proton (4058 Hz, 8.153 ppm) yielded the spectrum presented in Fig. 7, which is overlaid with the lowest energy geometry of **2** (Fig. 7a, τ ± 60), a local energy minimum of **2** (Fig. 7b, τ ± 180), and the global energy maximum of **2** (Fig. 7c, τ ± 120). Our interpretation of the 1D ^1^H NOE spectra is that the highest energy conformer is insignificantly populated - If it were populated, then NOE enhancement ‘4’ would be much stronger than enhancement ‘3’. The lowest energy conformer is dominant, even over the local minima at τ ≈ ± 180; both local minima place the imine C-H closer in space to the *ortho* aromatic-H than the α-CH_2_, meaning that NOE enhancement ‘1’ should be significantly stronger than enhancement ‘5’, however, the observed NOE enhancement ‘1’ is only 73.5% as intense as ‘5’.

**Figure 8:**
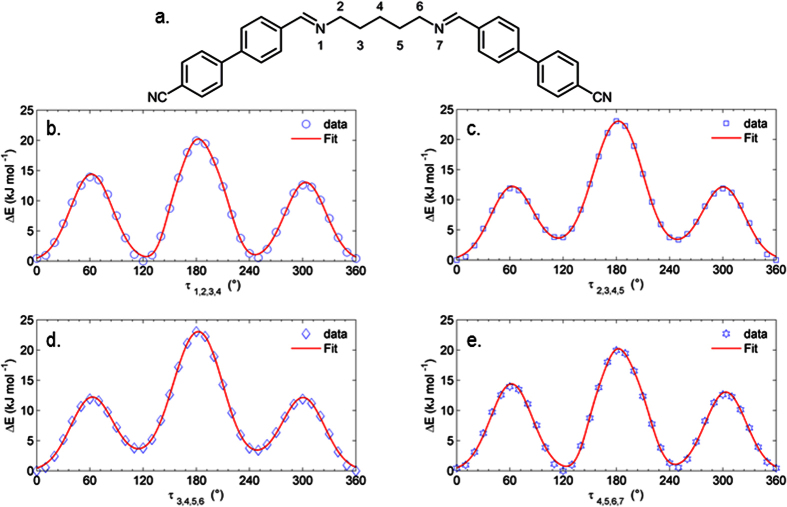
The dihedral angles labelled as τ are defined based on the atomic numbering shown in (**a**). Plots of energy as a function of each of the four dihedral angles present in the flexible portion of the spacer of compound **2** at the B3LYP/6-31G(d) level of theory (36 × 10° steps) are given along with 6-term Gaussian fits (**b–e**). Energies are given in kJ mol^−1^ and are relative to the lowest energy conformer.

Turning now to the other dihedrals angles present in the flexible spacer of compound **2** (Fig. 8), we see that in each case the *trans* conformer is the lowest energy, although for the degenerate dihedral angles τ_1,2,3,4_ and τ_4,5,6,7_ the *gauche* conformer at ± 120° is sufficiently low in energy (ΔE ≈1 kJ mol^−1^) that it is likely to be populated, while for the degenerate dihedral angles τ_2,3,4,5_ and τ_3,4,5,6_ the *trans* conformer is lower in energy than the *gauche* (ΔE = 3.6 kJ mol^−1^).

**Figure 9:**
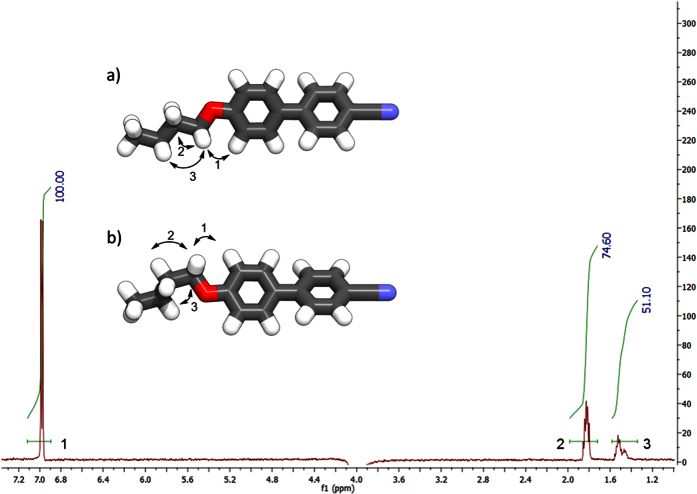
1D ^1^H NOESY spectra of compound **3** irradiated at 2027.15 Hz (4.052 ppm), which corresponds to the protons of the first methylene unit. Numbered black arrows indicate the assignment of observed NOE enhancements which are mapped onto the B3LYP/6-31G(d) geometries of the *trans* (**a**, τ = 0) and *gauche* conformers of **3** (**b**, τ = ±120). Numbers correspond to integrated intensities of the observed NOE enhancements, which have been arbitrarily set to a maximum of 100.

Essentially the influence of the linking group does not go beyond the first dihedral, which is to say that the remainder of the spacer behaves as if it were a linear hydrocarbon and preferentially adopts an all *trans* conformation. This behaviour is mirrored in compounds **1**, **3** and **4**; further plots of the dihedral angles *versus* energy for compounds **1**–4 are given in the ESI to this article. For compound **3** calculated torsional potentials suggested that the first dihedral angle is *gauche* rather than *trans*, however, when we opted to study this further by NOESY NMR we unexpectedly find that the all *trans* conformer appears dominant (Fig. 9).

Using the distance between the first methylene unit and the aromatic proton as a standard distance (2.33 Å for both *trans* and *gauche*) along with the well-known relationship between internuclear distance and the magnitude of NOE enhancement 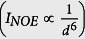 the observed NOE intensities can be used to give qualitative estimates of the internuclear distances. As this technique gives distances within ±10% of their actual value^37^ we cannot discriminate directly between *gauche* and *trans* conformers directly, as the difference in computed interatomic distances lies within this limit. However, for the *trans* and *gauche* conformers the difference in the hydrogen-hydrogen internuclear distance between the first and third methylene units is appreciably larger. From the B3LYP/6-31G(d) minimised geometry this distance is an average of 2.85 Å for *trans* and 3.30 Å for gauche, compared with a ‘measured’ NOESY NMR value of 2.64 Å. In any case, studying the selective NOE spectra of the methylene linked dimer **1** (CB9CB) reveals near identical NOE enhancements to those of **3** which, *inter alia*, are indicative of a near identical distribution of conformers. The implication is that the all *trans* conformer is dominant, contrary to what is suggested by DFT calculations. If the single gauche conformer was significantly populated, as has been reported recently based on DFT calculations for the dimer CB6OCB^38^, then the increased interatomic distance between the protons of the first and third methylene units would be expected to lead to a large increase in the internuclear distance as determined by selective 1D ^1^H NOESY NMR.

**Figure 10:**
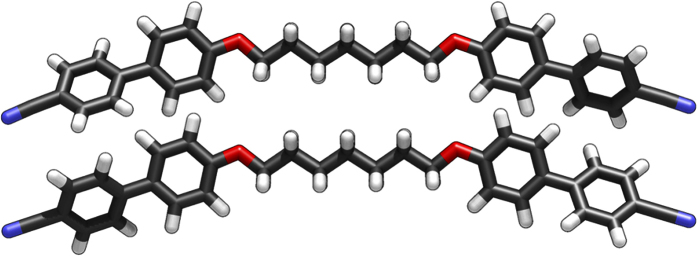
The molecular structure of compound **3** obtained by single crystal X-ray diffraction (top, R-factor 5.2)^39^ and the all *trans* structure obtained at the B3LYP/6-31G(d) level of DFT.

It is possible that there is a significant difference in conformational distribution between DFT calculations on isolated molecules and NMR spectra recorded in dilute solution, however, insight into conformational distribution gained from analysis of dielectric strength of the ether-linked dimer FFO9OCB suggests the all *trans* conformer is significantly populated in the bulk nematic phase^39^. In any case, the crystal structure of compound **3** has been reported previously and is given in Fig. 10 40, we note the structure from single crystal XRD is the all *trans*, matching that determined by selective 1D ^1^H NOESY NMR. While there is clearly significant merit in calculated geometries, we must add that we feel that the experiment – in this instance XRD and 1D ^1^H NOESY NMR - trumps theory.

**Figure 11:**
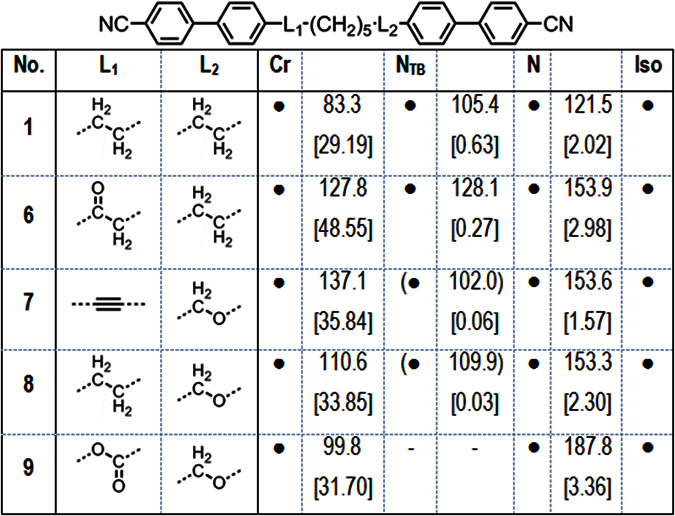
Transition temperatures (°C) and associated enthalpies of transition [kJ mol^−1^] for compounds **1** and **6**–**9**; transitions in parenthesis () are monotropic, *i.e.* they occur below the melting point of the sample.

Next we opted to screen a selection of cyanobiphenyl dimers with two different linking groups. The transition temperatures of compounds **6**–**9**, with compound **1** for comparison, are given in Fig. 11.

**Figure 12:**
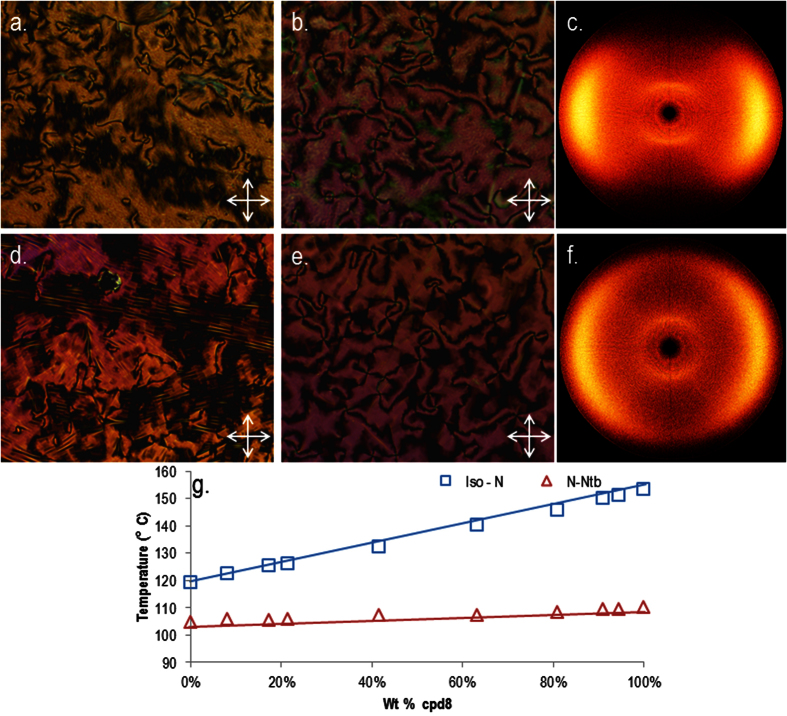
Photomicrographs (x100) of the nematic phase of compound **7** (**a**, 115 °C), the nematic phase of compound **8** (**b**, 143 °C), the N_TB_ phase of compound **7** (**d**, 95 °C), and the N_TB_ phase of compound **8** (**e**, 109 °C). Two dimensional small angle X-ray scattering patterns for the magnetically aligned nematic phase of compound **7** at 120 °C (**c**) and the partially aligned N_TB_ phase of compound **7** at 99 °C (**f**). Phase diagram for binary mixtures of CB9CB and compound **8** (**g**).

Replacement of one methylene unit of **1** with a carbonyl moiety to afford compound **6** yields dramatic increases in the thermal stability of both the nematic and N_TB_ phases. Compound **6** also exhibits a notable increase in melting point compared to **1**. The mixed alkyne/ether linked compound **7** has a comparable N_TB_-N transition temperature to **1**, however the clearing point of the former is over 30 °C higher than the latter. The mixed methylene/ether linked compound **8** was prepared by selective hydrogenation of **7**. Compound **8** exhibits a similar clearing point to that of **7**; however there is a moderate increase in the N-N_TB_ transition temperature of **8**. The mixed carboxylate ester/ether linked **9** exhibits a comparable clearing point to that of the diester compound **4**, however this material does not exhibit the twist-bend phase. Representative photomicrographs of the nematic and twist-bend phases of compounds **7** and **8** are given in Fig. 12. The identification of the N_TB_ phase in **7** and **8** was supported by small angle X-ray scattering studies (Fig. 12) which showed that the lower temperature phase (*i.e.* the N_TB_) lacks lamellar ordering, instead showing only diffuse nematic-like scattering patterns. The identity of the N_TB_ phase in compounds **6**, **7** and **8** was confirmed unambiguously by the construction of binary phase diagrams with CB9CB, itself known to exhibit the N_TB_ mesophase. The CB9CB/compound **8** phase diagram is presented in Fig. 8; the analogous phase diagrams for compounds **6** and **7** are given in the ESI to this article.

**Figure 13:**
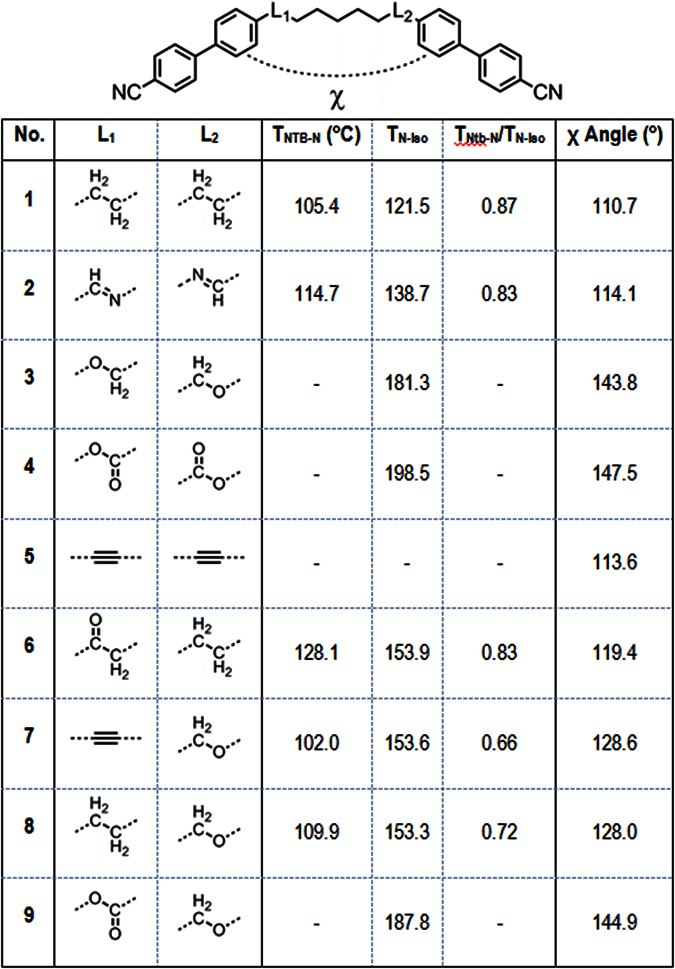
Temperature of the nematic to N_TB_ transition (°C), scaled N_TB_ transition temperature (T_Ntb_-N/T_N-Iso_) and the inter-aromatic angle χ as determined by geometries optimised using the B3LYP functional and the 6-31G(d) basis set.

Knowing that the all *trans* geometry is dominant we chose to study the relationship between the linking groups and the thermal stability of both the nematic and twist-bend nematic phases. Having already excluded polarity (Fig. 4) and flexibility (Figs 5 and 8) we next considered that the angle formed between the two mesogenic units may be of key importance. Indeed, theoretical treatments have postulated a link between the inter-aromatic angle and the incidence of the N_TB_ phase^41^. Although often widely assumed to be true this hypothesis this has not been tested experimentally, to the best of our knowledge. The geometry optimisation of compounds **1**–**9** was performed using the B3LYP functional and the 6-31G(d) basis set, analysis of the atomic Cartesian coordinates allows the inter-aromatic angle, shown as χ in Fig. 13, to be determined. Although this provides an angle for the minimum energy conformer it does not provide any information about the conformational landscape, or the distribution of conformers. However, it is implied that the all *trans* conformer is dominant^42^,^43^.

Compounds **3**, **4** and **9**, which have the largest inter-aromatic angles, do not exhibit the twist-bend nematic phase, although all three of these compounds exhibit relatively high clearing points compared to the other materials in Fig. 13. The lack of mesomorphic behaviour for compound **5**, despite having a comparable inter-aromatic angle to **1** and **2**, suggests that the rigidity imposed by the two alkyne groups is detrimental to both the N_TB_ and nematic phases. Given the reported non-linearity of the N-N_TB_ transition temperature as a function of concentration for ether linked^33^ and ester linked bimesogens^34^ with the CBnCB compounds, calculation of virtual transition temperatures for compounds **3**, **4**, **5** and **9** was not undertaken.

## Discussion

**Figure 14:**
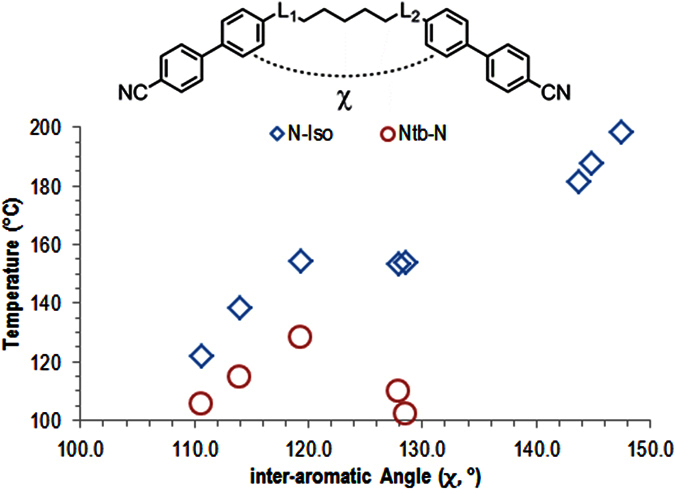
Plot of the nematic to isotropic (◊) and twist-bend nematic to nematic (⚪) transition temperatures (°C) vs the inter-aromatic angle, as determined computationally at the B3LYP/6-31G(d) level of theory.

**Figure 15:**
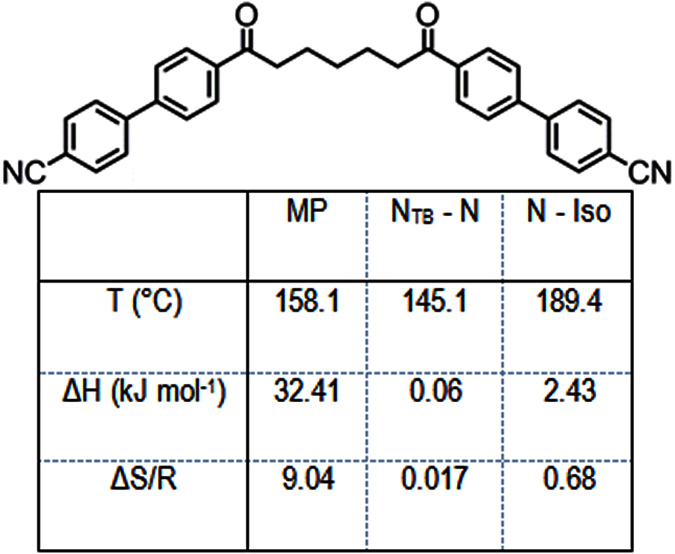
Transition temperatures (°), associated enthalpies (kJ mol^−1^) and associated dimensionless entropies (ΔS/R) of transition for the diketone linked compound, 10.

As shown in Fig. 14 both the nematic and twist-bend nematic phases exhibit a dependency on the inter-aromatic angle. The stability of the nematic phase appears to reach an apparent plateau between 120° and 130° before increasing once again. The thermal stability of the N_TB_ phase is seen to increase with increasing inter-aromatic angle at first, up to around 120° (compound **6**), before the thermal stability decreases again with further increases in the inter-aromatic angle (compounds **7** and **8**). In the binary phase diagrams of ether-linked^33^ and ester-linked^34^ cyanobiphenyl dimers with the CBnCB compounds it has been reported that the thermal stability of the N_TB_ phase initially *increases* before falling away. The reported maximum thermal stability occurs at 45 wt % ether linked cyanobiphenyl dimer and 40 wt% for the ester linked cyanobiphenyl dimer. This reported stabilisation may have its origins in the angle dependency; if we consider the phase diagrams not as a function of weight percent but instead a weighted average of the two inter-aromatic angles in question, we find the stabilisation in both cases corresponds to weighted average inter-aromatic angle of 125°. This angle resides between that of compounds **6** (χ = 119.4°) and compound **8** (χ = 128.0°). Our calculations show that where both L_1_ and L_2_ are ketones (*i.e.* the symmetric variant of **6**) the inter-aromatic angle is 125°. This compound has resisted our efforts to synthesize it to date; however, no such difficulties were encountered with the heptamethylene analogue (**10**, Fig. 15) Compound **10** exhibits dramatic increases in melting point and clearing point compared to both CB7CB and CB9CB (compound **1**), while the temperature of the nematic to twist-bend phase transition occurs at a much higher temperature than seen for **1**–**9**. The large increase in thermal stability, as judged by the onset temperature of the N_TB_ phase, strongly supports our view that a bend angle in the region of 125° is the optimum for a material to exhibit the twist-bend nematic phase, confirming that this phenomenon is not restricted to nonamethylene equivalent cyanobiphenyl dimers.

**Figure 16:**
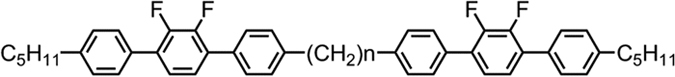
The chemical structures of DTC5C7 (n = 7) and DCT5C9 (n = 9)^7^.

Recently it has been reported that the liquid crystal bimesogen CB9CB (referred to as compound **1** in this work) and DTC5C9 and DTC5C7 (Fig. 16) exhibit an increase in the nematic to isotropic transition temperature when subjected to a large external magnetic field (22 T)^44^. This field induced increase is reported to be due to an increase in the inter-aromatic angle (*i.e.* the molecules become more linear). Such behaviour compliments and supports our observation that both T_N-Iso_ and T_NTB-N_ exhibit a strong dependence on the inter-aromatic angle. It is also possible that for a bimesogen with an even spacer parity, a transition to the N_TB_ phase may be induced on application of an external field to ‘bend’, rather than ‘straighten’, the molecule. This could be realised by synthesising a dimer employing mesogenic units with differing signs of diamagnetic or dielectric anisotropy, similar to the ether-linked bimesogens have reported previously^14^,^19^. Patterson *et. al.* have very recently demonstrated a N_TB_ phase transition driven by the photoisomerisation of an azo group; this is an elegant demonstration of the fact that the ability to change the bend angle can give rise to (or eliminate) the N_TB_ phase^45^. Such results confirm to us the fact that the incidence of the N_TB_ phase is ultimately a product of the gross topology of the molecule, and thus the minimisation of free volume that leads to condensed phases^38^,^46^.

## Additional Information

**How to cite this article**: Mandle, R. J. *et al*. The Dependency of Nematic and Twist-bend Mesophase Formation on Bend Angle. *Sci. Rep.* **6**, 36682; doi: 10.1038/srep36682 (2016).

**Publisher’s note**: Springer Nature remains neutral with regard to jurisdictional claims in published maps and institutional affiliations.

## Supplementary Material

Supplementary Information

## Figures and Tables

**Figure 1:**
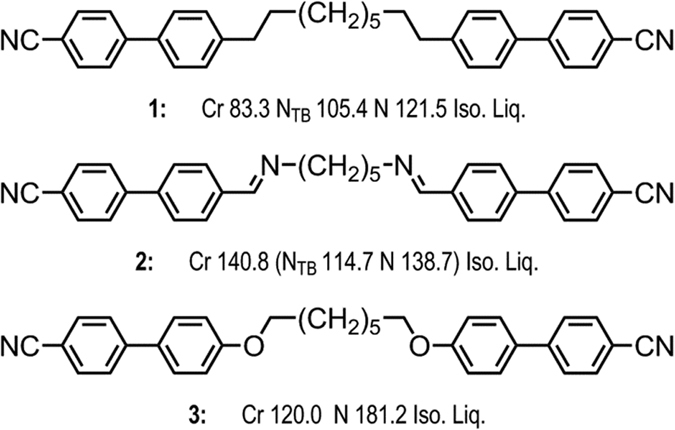
Molecular structure and transition temperatures (°C) of compounds **1** (CB9CB, top)^6^,^11^,^27^, **2** (CBI-5-ICB, middle) and **3** (CBO7OCB, bottom)^30^.
