# Supplementary material for: The Dependency of Nematic and Twist-bend Mesophase Formation on Bend Angle
Source: Sci Rep. 2016 Nov 7;6:36682. doi: 10.1038/srep36682 (PMC5098182; doi:10.1038/srep36682)
Supplement: Supplementary Information [file srep36682-s1.pdf]

Supplementary Information for

The Dependency of Nematic and Twist-bend Mesophase Formation on Bend Angle

Richard J. Mandle*, Craig T. Archbold, Julia P. Sarju, Jessica L. Andrews and John W. Goodby

Department of Chemistry, the University of York, Heslington, York, YO10 5DD.

1.1. General Methods.

All chemicals were purchased from Sigma-Aldrich or Apollo Scientific and used as received, without further purification. Solvents were dried by percolation through a column of activated alumina prior to use. Compound ***i4*** was prepared as described previously [1]. CBO7OCB was prepared *via* the alkylation of 4-hydroxy-4'-cyanobiphenyl with 1,7-dibromoheptane according to the procedure of Elmsley *et. al*. [2] CB9CB was prepared using an analogous procedure to that described previously by us for CB11CB. [1]

NMR spectra were recorded on a JEOL ECS spectrometer operating at 400 MHz (^1H), 100.5 MHz ($^{13}C\{^1H\}$) as solutions in $CDCl_3$, unless stated otherwise. One dimensional ^1H NOE NMR experiments were performed on a Bruker AVANCE 500 spectrometer operating at 500 MHz (pulse program selnogp [3-5]) at a temperature of 298 K and on samples in solution in $CDCl_3$.

Mass spectra were recorded on a Bruker micrOTOF MS-Agilent series 1200LC spectrometer. FTIR spectroscopy was performed using a Shimadzu IR Prestige-21 with Specac Golden Gate diamond ATR IR insert. High-performance liquid chromatography was performed on a Shimadzu Prominence modular HPLC system comprising a LC-20A quaternary solvent pump, a DGU-20A_5 degasser, a SIL-20A autosampler, a CBM-20A communication bus, a CTO-20A column oven, and a SPO-20A dual wavelength UV-vis detector. The column used was an Alltech C18 bonded reverse-phase silica column with a 5 µm pore size, an internal diameter of 10 mm and a length of 250 mm. In all cases the purity of final compounds, as determined by HPLC, was found to be in excess of 99%.

Polarised optical microscopy was performed on a Zeiss Axioskop 40Pol microscope using a Mettler FP82HT hotstage controlled by a Mettler FP90 central processor. Photomicrographs were captured *via* an InfinityX-21 MP digital camera mounted atop the microscope. Differential scanning calorimetry was performed on a Mettler DSC822^e fitted with an autosampler operating with Mettler Star^e software and calibrated before use against an indium standard (onset = 156.55 ± 0.2 °C, ΔH = 28.45 ± 0.40 Jg^{-1}) under an atmosphere of dry nitrogen. DSC thermograms were obtained at a heat/cool rate of 10 °C min^{-1}, with the presented enthalpy values being the average of three runs. Computational studies were performed using Gaussian 09 rev E.01, [6] with molecular structures rendered in Qutemol. [7]

Small angle X-ray diffraction was performed using a Bruker D8 Discover equipped with a temperature controlled, bored graphite rod furnace, custom built at the University of York. The radiation used was copper Kα (λ =

0.154056 nm) from a 1 μS microfocus source. Diffraction patterns were recorded on a 2048x2048 pixel Bruker VANTEC 500 area detector. Samples were filled into 1mm capillary tubes and aligned with a pair of 1T magnets. Diffraction patterns were collected as a function of temperature and the data processed using Matlab. Raw data are available upon request from the University of York data catalogue.

1.2.1. Synthesis of Chemical Intermediates

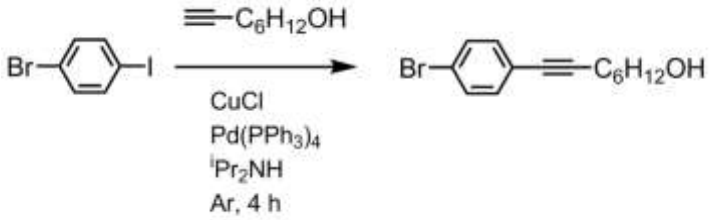

i1**:** **8-(4-Bromophenyl)oct-7-yn-1-ol**,

A solution of 1-bromo-4-iodobenzene (2.24 g, 7.94 mmol) and oct-7-yn-1-ol (1 g, 7.94 mmol) in freshly distilled diisopropylamine (50 ml) was thoroughly degassed by sparging with argon for 0.5 h. To the degassed solution copper (I) chloride (50 mg) and $Pd(PPh_3)_4$ (50 mg) were added in one portion. The reaction was monitored by TLC, with the consumption of 1-bromo-4-iodobenzene (Rf_{hexane} = 0.35) and the formation of ***i1*** (Rf_{DCM} = 0.4). Upon completion (< 4 h), the diisopropylamine was recovered by distillation leaving a solid mass. This mass was partitioned between water and dichloromethane (1:1, 100 ml). The organic layer was separated; the aqueous washed with dichloromethane (3 x 25 ml) and discarded. The combined organic layers were washed with brine, dried with $MgSO_4$ and concentrated *in vacuo*. The crude material was purified column chromatography with a gradient of hexanes/DCM as the eluent (Rf_{DCM} = 0.4) affording ***i1*** as a pale yellow oil.

Yield:	1.9 g (85 %)
^{1}H NMR (400 MHZ, $CDCl_3$):	1.31 – 1.49 (4H, m, -C$\underline{H}_2$ -), 1.51 – 1.62 (4H, m, -C$\underline{H}_2$-), 1.85 (1H, broad S, CH_2-O$\underline{H}$), 2.36 (2H, t, J_{H-H} = 7.0 Hz, Ar-CΞC-$\underline{CH_2}$-CH_2), 3.61 (2H, t, J_{H-H} = 7.0 Hz, -C$\underline{H}_2$-OH), 7.21 (2H, ddd, J_{H-H} = 1.8 Hz, J_{H-H} = 2.3 Hz, J_{H-H} = 8.7 Hz, Ar$\underline{H}$), 7.37 (2H, ddd, J_{H-H} = 1.8 Hz, J_{H-H} = 2.3 Hz, J_{H-H} = 8.7 Hz, Ar$\underline{H}$)
^{13}C{^{1}H} NMR (100.5 MHZ, $CDCl_3$):	19.14, 25.10, 28.34, 28.49, 32.30, 62.32, 79.48, 91.40, 121.33, 122.75, 131.15, 132.78
FT-IR (ν max, cm-1):	700, 821, 1010, 1070, 1166, 1178, 1192, 1263, 1485, 1726, 2850, 2922, 3329 (broad)

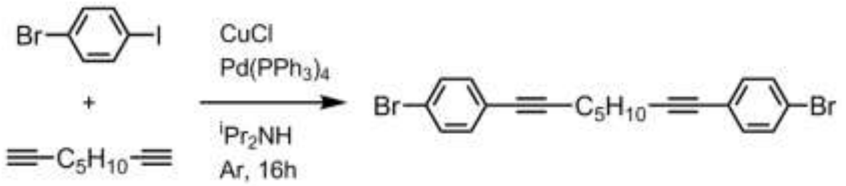

i2: **1,9-*bis*(4-bromophenyl)nona-1,8-diyne**

Quantities used: 4-bromoiodobenzene (20 g, 70 mmol), 1,8-nonadiyne (5 ml, 4 g, 33.3 mmol) freshly distilled diisopropylamine (150 ml), copper chloride (<100 mg) and $Pd(PPh_3)_4$ (<100 mg). The experimental procedure was as described for compound ***i1***. The title compound was purified by column chromatography with hexanes as the eluent ($Rf_{hexanes}$ = 0.3) affording the title compound as a white solid following recrystalisation from petroleum ether 40-60.

Yield:	10.4 g (89%)
^{1}H NMR (400 MHz, $CDCl_3$):	1.59 – 1.67 (6H, m, Ar-CC-CH_2-<u>$(CH_2)_3$-</u>CH_2-CC-Ar), 2.38 – 2.43 (4H, m Ar-CC-<u>CH_2</u>-$(CH_2)_3$-<u>CH_2</u>-CC-Ar), 7.19 (4H, ddd, J_{H-H} = 1.8 Hz, J_{H-H} = 2.8 Hz, J_{H-H} = 8.2 Hz, Ar<u>H</u>), 7.36 (4H, ddd, J_{H-H} = 1.8 Hz, J_{H-H} = 2.8 Hz, J_{H-H} = 8.2 Hz, Ar<u>H</u>)
$^{13}C\{^1H\}$ NMR (100.5 MHZ, $CDCl_3$):	19.32, 28.04, 79.83, 91.33, 121.58, 122.89, 131.38, 132.97
FT-IR (ν max, cm-1):	704, 732, 773, 821, 960, 995, 1008, 1066, 1095, 1172, 1259, 1327, 1346, 1392, 1462, 1483, 1583, 1901, 2235, 2856, 2887, 2937

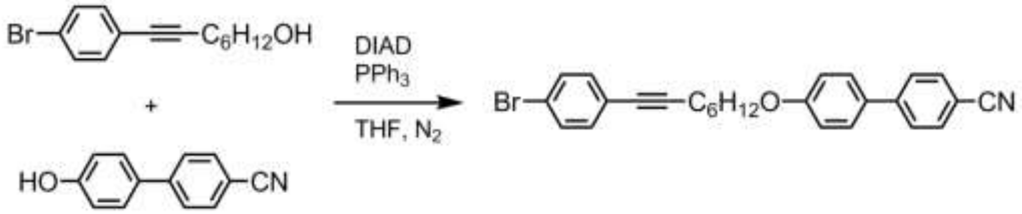

i3: **4'-((8-(4-Bromophenyl)oct-7-yn-1-yl)oxy)-[1,1'-biphenyl]-4-carbonitrile**

DIAD (383 mg, 373 µl, 1.9 mmol) was added dropwise to a solution of ***i1*** (0.5 g, 1.78 mmol), 4-hydroxy-4'-cyanobiphenyl (1.9 mmol, 371 mg) and triphenylphosphine (498 mg, 1.9 mmol) in anhydrous THF (10 ml) under an atmosphere of dry nitrogen. The reaction solution was stirred until complete consumption of ***i1*** (<2 h). The solvent was removed *in vacuo* and the crude residue purified by column chromatography with a gradient of hexanes/DCM as the eluent (Rf_{DCM} = 0.9) and recrystalisation from n-hexanes.

Yield:	0.65 g (77%)
^{1}H NMR (400 MHZ, $CDCl_3$):	1.48 (4H, m, -CH_2-), 1.63 (2H, quintet, J_{H-H} = 6.9 Hz, Ar-CΞC-CH_2-<u>CH_2</u>-CH_2) 1.83 (2H, quintet, J_{H-H} = 6.9 Hz, Ar-O-CH_2-<u>CH_2</u>-CH_2), 2.39 (2H, t, J_{H-H} = 6.9 Hz, Ar-CΞC-<u>CH_2</u>-CH_2-), 4.00 (2H, t, J_{H-H} = 6.9 Hz, Ar-O-<u>CH_2</u>-CH_2), 6.96 (2H, ddd, J_{H-H} = 1.8 Hz, J_{H-H} = 2.3 Hz, J_{H-H} = 8.7 Hz, Ar<u>H</u>) 7.22 (2H, ddd, J_{H-H} = 1.8 Hz, J_{H-H} = 2.3 Hz, J_{H-H} = 8.7 Hz, Ar<u>H</u>), 7.37 (2H, ddd, J_{H-H} = 1.8 Hz, J_{H-H} = 2.3 Hz, J_{H-H} = 8.7 Hz, Ar<u>H</u>), 7.50 (2H, ddd, J_{H-H} = 2.3 Hz, J_{H-H} = 2.8 Hz, J_{H-H} = 9.2 Hz, Ar<u>H</u>), 7.62 (2H, ddd, J_{H-H} = 1.8 Hz, J_{H-H} = 1.8 Hz, J_{H-H} = 8.7 Hz, Ar<u>H</u>), 7.67 (2H, ddd, J_{H-H} = 1.8 Hz, J_{H-H} = 1.8 Hz, J_{H-H} = 8.7 Hz, Ar<u>H</u>)
$^{13}C\{^1H\}$ NMR (100.5 MHZ, $CDCl_3$):	19.31, 25.54, 28.40, 28.52, 29.02, 67.92, 79.71, 91.45, 109.96, 115.00, 119.08, 121.53, 122.90, 127.00, 128.27, 131.22, 131.35, 132.51, 132.95, 145.16, 159.67
FT-IR (ν max, cm-1):	659, 704, 723, 738, 821, 846, 856, 937, 954, 999, 1010, 1031, 1055, 1070, 1095, 1112, 1176, 1217, 1228, 1267, 1292, 1309, 1365, 1425, 1471, 1485, 1492, 1519, 1551, 1604, 2223, 2939, 2970

1.2.2. Synthesis of Liquid Crystal Dimers

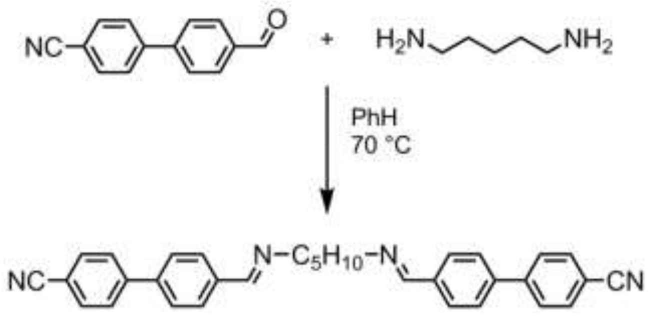

2: 4',4'''-((1E,1'E)-(pentane-1,5-diylbis(azanylylidene))bis(methanylylidene))bis(([1,1'-biphenyl]-4-carbonitrile))

1,5-Diaminopentane (100 mg, 87 µl, 0.95 mmol) was added in one portion to a stirred solution of 4'-formyl-[1,1'-biphenyl]-4-carbonitrile (500 mg, 1.9 mmol) in anhydrous benzene (10 ml) at 70 °C under an atmosphere of dry nitrogen. The heating was continued for 16 h, the solution was then cooled and petroleum ether 40:60 added to induce precipitation of the title compound, which was collected as a pale yellow solid.

Yield:	300 mg (66%)
^{1}H NMR (400 MHz, $CDCl_3$):	1.42 – 1.52 (2H, m, -CH_2-C$\underline{H}_2$-CH_2-), 1.78 (4H, Quintet, J_{H-H} = 7.8 Hz, ArCHN-CH_2-C$\underline{H}_2$-CH_2-C$\underline{H}_2$-CH_2-NHCAr), 3.65 (4H, t, J_{H-H} = 7.8 Hz, ArCHN-C$\underline{H}_2$-$(CH_2)_3$-C$\underline{H}_2$-NHCAr), 7.61 (4H, ddd, J_{H-H} = 1.8 Hz, J_{H-H} = 3.0 Hz, J_{H-H} = 8.4 Hz, Ar$\underline{H}$), 7.66 – 7.73 (8H, m, Ar$\underline{H}$), 7.81 (4H, ddd, J_{H-H} = 1.8 Hz, J_{H-H} = 2.3 Hz, J_{H-H} = 8.7 Hz, Ar$\underline{H}$), 8.31 (2H, s, ArC$\underline{H}$N-CH_2)
^{13}C{^{1}H} NMR (100.5 MHZ, $CDCl_3$):	25.04, 30.63, 61.69, 111.26, 118.77, 127.38, 127.66, 128.69, 132.61, 136.45, 140.93, 144.79, 160.06
FT-IR (ν max, cm-1):	715, 815, 945, 970, 1004, 1026, 1178, 1288, 1311, 1398, 1462, 1492, 1604, 1639, 2229, 2841, 2935, 3064
MS (ESI+, *m/z*):	503.2211 (calcd. for $C_{33}H_{28}N_4Na$: 503.2206, M + Na)<br>481.2400 (calcd. for $C_{33}H_{29}N_4$: 481.2387, M + H)

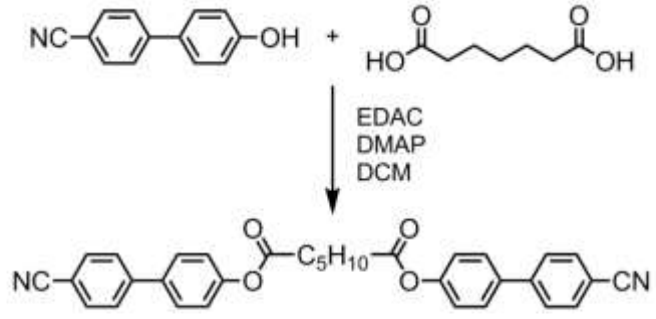

4: ***bis*(4'-Cyano-[1,1'-biphenyl]-4-yl) heptanedioate**

4-Hydroxy-4'-cyanobiphenyl (3.9 g, 2 mmol), pimelic acid (1.6 g, 1 mmol), EDAC (5.8 g, 3 mmol) and DMAP (10 mg) were suspended in dichloromethane (250 ml) and the resulting suspension stirred for 16 hours. The solvent was removed *in vacuo* and the crude material purified by flash chromatography with DCM as the eluent, followed by recrystalisation from 20:1 ethanol/THF to afford the title compound as a white solid.

Yield:	3.7 g (74%)
^{1}H NMR (400 MHz, $CDCl_3$):	1.52 – 1.62 (2H, m, -CH_2-C<u>H</u>$_2$-CH_2-), 1.85 (4H, Quintet, J_{H-H} = 7.3 Hz, ArOOC-CH_2-C<u>H</u>$_2$-CH_2-C<u>H</u>$_2$-CH_2-COOAr), 2.63 (4H, t, J_{H-H} = 7.3 Hz, ArOOC-C<u>H</u>$_2$-$(CH_2)_3$-C<u>H</u>$_2$-OOCAr), 7.19 (4H, ddd, J_{H-H} = 2.3 Hz, J_{H-H} = 2.8 Hz, J_{H-H} = 8.7 Hz, Ar<u>H</u>), 7.57 (4H, ddd, J_{H-H} = 2.3 Hz, J_{H-H} = 2.8 Hz, J_{H-H} = 8.7 Hz, Ar<u>H</u>), 7.63 (4H, ddd, J_{H-H} = 1.8 Hz, J_{H-H} = 2.3 Hz, J_{H-H} = 8.7 Hz, Ar<u>H</u>), 7.70 (4H, ddd, J_{H-H} = 1.8 Hz, J_{H-H} = 2.3 Hz, J_{H-H} = 8.7 Hz, Ar<u>H</u>)
$^{13}C\{^1H\}$ NMR (100.5 MHZ, $CDCl_3$):	24.43, 28.44, 34.06, 110.97, 118.80, 122.26, 127.61, 128.29, 132.60, 136.76, 144.66, 151.05, 171.88
FT-IR (ν max, cm-1):	713, 746, 792, 817, 842, 889, 920, 1004, 1020, 1049, 1082, 1107, 1143, 1168, 1192, 1226, 1302, 1365, 1464, 1494, 1606, 1639, 1747, 2229, 2333, 2366, 2831, 2862, 2941, 3072
MS (ESI+, *m/z*):	537.1799 (calcd. for $C_{33}H_{26}N_2NaO_4$: 53.1785, M + Na)

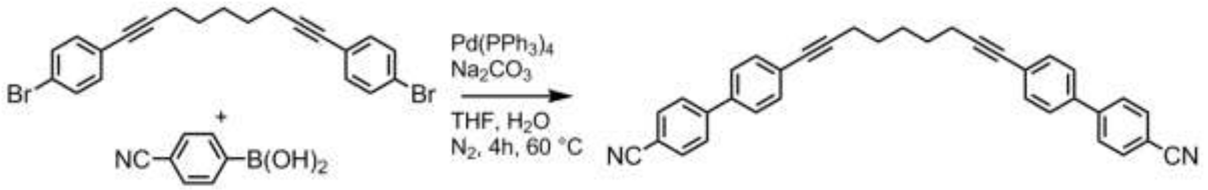

5: 4',4'''-(nona-1,8-diyne-1,9-diyl)bis(([1,1'-biphenyl]-4-carbonitrile))

Quantities used: ***i1*** (1000 mg, 2.33 mmol), 4-cyanophenylboronic acid (6 mmol, 882 mg), $Pd(PPh_3)_4$ (<25 mg), aqueous sodium carbonate (2M, 75 ml), THF (75 ml). The experimental procedure was as described for compound i**3**. The crude material was purified by column chromatography with 3:1 hexanes/DCM as the eluent (Rf_{DCM} = 0.5) and was obtained as fine colourless needles following recrystalisation from ethanol/THF (approx. 10:1).

Yield:	950 mg (86%)
^{1}H NMR (400 MHz, $CDCl_3$):	1.66 – 1.74 (6H, m, Ar-CC-CH_2-$\underline{(CH_2)_3}$-CH_2-CC-Ar), 2.49 (4H, t, $J_{H\text{-}H}$ = 6.4 Hz, Ar-CC-$\underline{CH_2}$-$(CH_2)_3$-$\underline{CH_2}$-CC-Ar), 7.48 (8H, s, Ar$\underline{H}$), 7.64 (4H, ddd, $J_{H\text{-}H}$ = 1.4 Hz, $J_{H\text{-}H}$ = 1.8 Hz, $J_{H\text{-}H}$ = 8.7 Hz, Ar$\underline{H}$), 7.70 (4H, ddd, $J_{H\text{-}H}$ = 1.4 Hz, $J_{H\text{-}H}$ = 1.8 Hz, $J_{H\text{-}H}$ = 8.7 Hz, Ar$\underline{H}$)
$^{13}C\{^1H\}$ NMR (100.5 MHZ, $CDCl_3$):	19.41, 28.12, 28.14, 80.29, 91.96, 111.02, 118.80, 124.52, 126.93, 127.45, 132.22, 132.59, 137.96, 144.80
FT-IR (ν max, cm-1):	713, 808, 819, 856, 1004, 1109, 1217, 1228, 1365, 1427, 1489, 1602, 2223, 2862, 2970
MS (ESI+, *m/z*):	497.1998 (calcd. for $C_{35}H_{26}N_2Na$: 497.1988, M + Na)

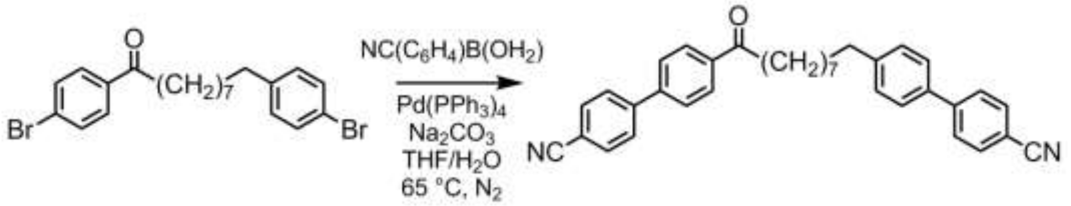

6: 4',4'''-(1-Oxononane-1,9-diyl)*bis*(([1,1'-biphenyl]-4-carbonitrile))

A solution of 1,9-*bis*(4-bromophenyl)nonan-1-one (***i4***, 200 mg, 0.44 mmol) in a biphasic mixture of THF (20 ml) and 2M aqueous sodium carbonate (20 ml) were degassed by sparging with nitrogen whilst agitating in an ultrasonic bath. The suspension was heated under reflux whilst vigorously stirring before the addition of 4-cyanobenzene boronic acid (147 mg, 1 mmol) and $Pd(PPh_3)_4$ (50 mg) in on portion. Heating was continued until complete consumption of the starting materials. The reaction was cooled, the biphasic mixture separated, the aqueous layer washed with DCM (3 x 15 ml) and discarded. The combined organic extracts were dried over $MgSO_4$, filtered through a plug of celite and concentrated *in vacuo*. The crude material was purified by column chromatography with DCM/hexanes as the eluent (2:1), followed by recrystalisation of the isolated material from ethanol, affording **6** as a fine white powder.

Yield:	185 mg (85%)
^{1}H NMR (400 MHZ, $CDCl_3$):	1.31 – 1.42 (8H, m, -CH_2-$\underline{(CH_2)_4}$-CH_2), 1.63 (2H, quintet, J_{H-H} = 7.0 Hz, Ar-CH_2-$\underline{CH_2}$-$(CH_2)_6$C(O)-Ar), 1.74 (2H, quintet, J_{H-H} = 7.0 Hz, ArC(O)-CH_2-$\underline{CH_2}$-$(CH_2)_6$-Ar), 2.63 (2H, t, J_{H-H} = 7.0 Hz, Ar-$\underline{CH_2}$-$(CH_2)_7$-C(O)-Ar), 2.97 (2H, t, J_{H-H} = 7.0 Hz, Ar-C(O)-$\underline{CH_2}$-$(CH_2)_7$-Ar), 7.27 (2H, d, J_{H-H} = 8.2 Hz, Ar*H*), 7.49 (2H, d, J_{H-H} = 7.8 Hz, Ar*H*), 7.63 – 7.71 (8H, m, Ar*H*), 7.75 (2H, d, J_{H-H} = 8.7 Hz, Ar*H*), 8.04 (2H, d, J_{H-H} = 8.2 Hz, Ar*H*)
^{13}C{^{1}H} NMR (100.5 MHZ, $CDCl_3$):	24.22, 29.20, 29.28, 29.38, 31.33, 35.56, 38.70, 110.45, 111.79, 118.61, 119.00, 127.02, 127.39, 127.41, 127.85, 128.81, 129.13, 132.52, 132.70, 136.40, 136.77, 143.26, 143.67, 144.28, 145.54, 199.85
FT-IR (ν max, cm-1):	692, 721, 750, 773, 819, 835, 860, 964, 1002, 1114, 1178, 1321, 1365, 1465, 1492, 1602, 1680, 1737, 2222, 2854, 2926, 3028
MS (ESI+, *m/z*):	519.2412 (calcd. for $C_{35}H_{32}N_2NaO$: 519.2407, M + Na)

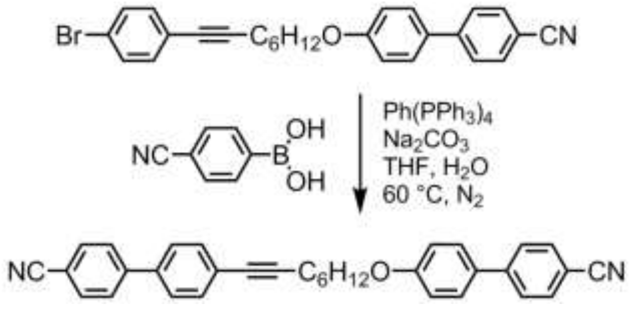

7: **4'-((8-(4'-Cyano-[1,1'-biphenyl]-4-yl)oct-7-yn-1-yl)oxy)-[1,1'-biphenyl]-4-carbonitrile**

Quantities used: ***i3*** (300 mg, 0.65 mmol), 4-cyanophenyl boronic acid (147 mg, 1 mmol), $Pd(PPh_3)_4$ (< 50 mg), 2M aqueous sodium carbonate (15 ml), THF (15 ml). The experimental procedure was as described in the synthesis of **6**, with the title compound obtained as fine white crystals following column chromatography with a gradient of DCM/hexanes as the eluent (Rf_{DCM} = 0.8) and recrystalisation from ethanol/THF (4:1).

Yield:	300 mg (96%)
^{1}H NMR (400 MHZ, $CDCl_3$):	1.52 – 1.59 (4H, m, -CH_2-), 1.63 – 1.72 (2H, m, -CH_2-), 1.81 – 1.91 (2H, m, -CH_2-), 2.47 (2H, t, J_{H-H} = 6.9 Hz, Ar-C-C-C<u>H</u>$\underline{H_2}$-CH_2), 4.00 (2H, t, J_{H-H} = 6.9 Hz, ArO-C$\underline{H_2}$-CH_2), 6.99 (2H, ddd, J_{H-H} = 2.3 Hz, J_{H-H} = 3.2 Hz, J_{H-H} = 8.7 Hz, Ar$\underline{H}$), 7.48 – 7.55 (6H, m, Ar$\underline{H}$), 7.60 – 7.74 (8H, m, Ar$\underline{H}$)
^{13}C{^{1}H} NMR (100.5 MHZ, $CDCl_3$):	19.41, 25.59, 28.51, 28.60, 29.07, 67.97, 80.19, 92.05, 109.99, 110.99, 115.02, 118.82, 119.07, 124.51, 126.94, 127.01, 127.46, 128.29, 131.24, 132.19, 132.52, 132.59, 137.97, 144.81, 145.17, 159.69.
FT-IR (ν max, cm-1):	727, 821, 854, 900, 1033, 1112, 1217, 1228, 1265, 1294, 1365, 1492, 1521, 1558, 1602, 2222, 2945, 2970
MS (ESI+, *m/z*):	503.2094 (calcd. for $C_{34}H_{28}N_2NaO$: 503.2094, M + Na)

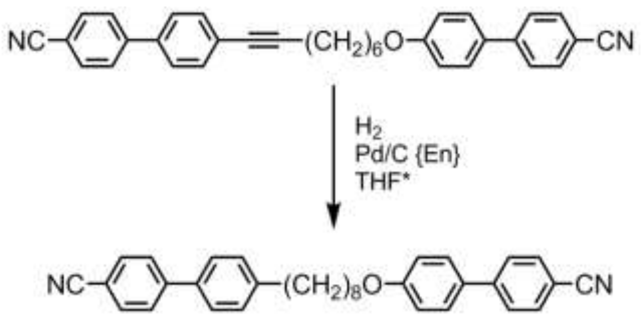

8: 4'-((8-(4'-cyano-[1,1'-biphenyl]-4-yl)octyl)oxy)-[1,1'-biphenyl]-4-carbonitrile

Palladium on carbon (5%, 5g) was poisoned with diethylamine by stirring in deoxygenated methanol (100 ml) and diethylamine (12 ml) for 24 hours. The suspension was filtered through a fine sinter, washed with methanol (2 x 40 ml) and hexane (2 x 40 ml) and dried by suction filtration for 24 h affording Pd/C {En}. THF was deoxygenated by sparging freshly distilled THF with argon for 4 hours. An oven dried reaction vial was charged with compound **7** (100 mg), Pd/C {En} (2 mg) and deoxygenated THF (3 ml) before fitting a septum cap. The suspension was vigorously stirred while sparging with hydrogen gas for 16 h. The reaction suspension was filtered through a pad of celite, eluting with dichloromethane. The solvents were removed *in vacuo* and the crude material recrystalised from DCM/hexanes (1:8) affording **8** as a white solid.

Note, no reduction of the aromatic nitrile to the primary amine was observable by either TLC or NMR analysis of the crude reaction mixture.

Yield: 85 mg (84 %)

^{1}H NMR (400 MHZ, $CDCl_3$): 1.33 – 1.51 (8H, m, -CH_2-), 1.59 – 1.69 (2H, m, -CH_2-), 1.79 (2H, quintet, $J_{H\text{-}H}$ = 6.9 Hz, -CH_2-), 2.64 (2H, t, $J_{H\text{-}H}$ = 6.9 Hz, Ar-CH_2-C<u>H</u>$_2$-CH_2), 4.98 (2H, t, $J_{H\text{-}H}$ = 6.9 Hz, ArO-C<u>H</u>$_2$-CH_2), 6.95 (2H, ddd, $J_{H\text{-}H}$ = 2.3 Hz, $J_{H\text{-}H}$ = 2.8 Hz, $J_{H\text{-}H}$ = 8.7 Hz, Ar<u>H</u>), 7.27 (2H, m, Ar<u>H</u>), 7.47 – 7.52 (4H, m, Ar<u>H</u>), 7.58 – 7.71 (8H, m, Ar<u>H</u>)

^{13}C{^{1}H} NMR (100.5 MHZ, $CDCl_3$): 26.00, 29.19, 29.20, 29.28, 29.39, 31.36, 35.59, 68.10, 110.03, 110.51, 115.03, 119.10, 127.05, 127.06, 127.45, 128.31, 129.16, 131.26, 132.55, 136.46, 143.68, 145.23, 145.56, 159.74.

FT-IR (ν max, cm-1): 661, 725, 785, 813, 850, 900, 1012, 1028, 1091, 1112, 1217, 1228, 1292, 1365, 1492, 1521, 1558, 1600, 1739, 2222, 2854, 2941, 2970, 3005, 3454

MS (ESI+, *m/z*): 507.2412 (calcd. for $C_{34}H_{32}N_2NaO$: 507.2407, M + Na)

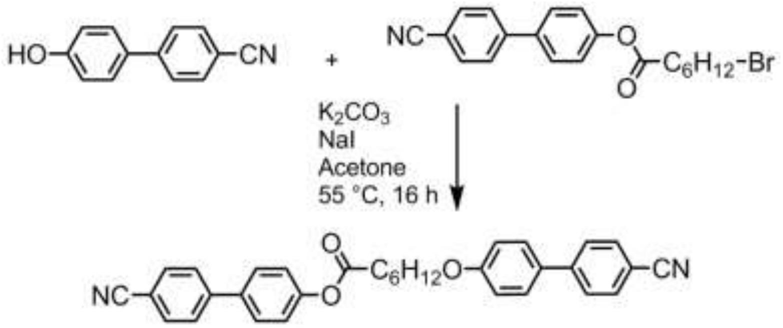

9: 4'-Cyano-[1,1'-biphenyl]-4-yl 7-((4'-cyano-[1,1'-biphenyl]-4-yl)oxy)heptanoate

A suspension of 4-hydroxy-4'-cyanobiphenyl (50 mg, 0.256 mmol), 4'-cyano-[1,1'-biphenyl]-4-yl 8-bromooctanoate (103 mg, 0.256 mmol), potassium carbonate (137 mg, 1 mmol) and sodium iodide (150 mg, 1 mmol) in acetone (10 ml) was heated under reflux with vigorous stirring for 16 h. The reaction solution was cooled, DCM (50 ml) and magnesium sulphate (5 g) added and after stirring for 5 minutes the insoluble matter was removed by filtration. The solvents were removed *in vacuo* and the crude material purified by flash chromatography with DCM/hexanes as the eluent. Recrystalisation of the chromatographed material from ethanol afforded compound **9** as a white solid.

Yield: 65 mg (49 %)

^{1}H NMR (400 MHZ, $CDCl_3$): 1.47 – 1.63 (4H, m, -CH_2-($C\underline{H}_2)_2$-CH_2-), 1.78 – 1.91 (4H, m, O-CH_2-$C\underline{H}_2$-$(CH_2)_2$-$C\underline{H}_2$-CH_2-COO), 2.62 (2H, t, J_{H-H} = 7.8 Hz, ArOOC-$C\underline{H}_2$-CH_2), 4.03 (2H, t, J_{H-H} = 7.8 Hz, ArO$C\underline{H}_2$-CH_2), 6.99 (2H, ddd, J_{H-H} = 2.3 Hz, J_{H-H} = 3.1 Hz, J_{H-H} = 8.4 Hz, Ar$\underline{H}$), 7.20 (2H, ddd, J_{H-H} = 2.3 Hz, J_{H-H} = 3.1 Hz, J_{H-H} = 8.4 Hz, Ar$\underline{H}$), 7.53 (2H, ddd, J_{H-H} = 2.3 Hz, J_{H-H} = 3.1 Hz, J_{H-H} = 8.4 Hz, Ar$\underline{H}$), 7.58 (2H, ddd, J_{H-H} = 2.3 Hz, J_{H-H} = 3.1 Hz, J_{H-H} = 8.4 Hz, Ar$\underline{H}$), 7.61 – 7.74 (8H, m, Ar$\underline{H}$)

$^{13}C\{^1H\}$ NMR (100.5 MHZ, $CDCl_3$): 24.77, 25.75, 28.80, 29.02, 34.25, 67.89, 110.05, 111.03, 115.04, 118.81, 119.08, 122.28, 127.06, 127.64, 128.32, 131.34, 132.55, 132.63, 136.78, 144.72, 145.21, 151.12, 159.68, 172.07, 192.53

FT-IR (ν max, cm-1): 661, 727, 798, 813, 858, 921, 997, 1128, 1165, 1178, 1205, 1217, 1228, 1247, 1269, 1290, 1365, 1463, 1473, 1492, 1598, 1743, 2222, 2937, 2970

MS (ESI+, *m/z*): 523.1998 (calcd. for $C_{33}H_{28}N_2O_3$: 523.1992, M + Na)

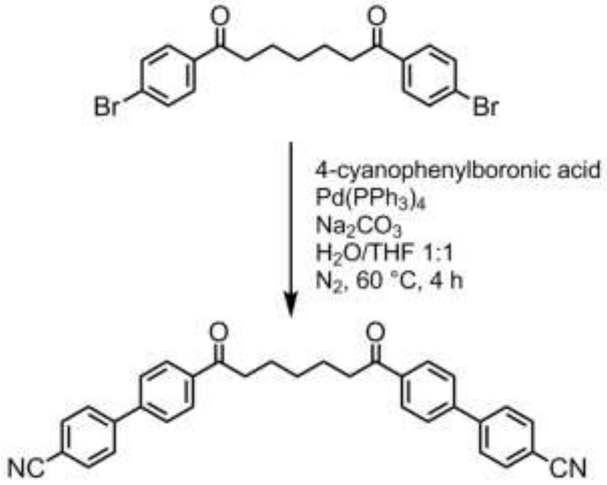

10: 4',4'''-Heptanedioylbis(([1,1'-biphenyl]-4-carbonitrile))

Quantities used: 4-cyanophenylboronic acid (350 mg, 2.40 mmol), 1,7-*bis*(4-bromophenyl)heptane-1,7-dione (500 mg, 1.14 mmol), $Pd(PPh_3)_4$ (10 mg), 2M aqueous sodium carbonate (20 ml) THF (20 ml). The experimental procedure was as described in the synthesis of compound **6**, with the exception that an extra 500 mg of 4-cyanoboronic acid was added after 24 h to complete the reaction. The crude material was purified by flash chromatography with DCM as the eluent, with the chromatographed material recrystalised from 10:1 ethanol THF to afford a white powder.

Yield:	210 mg (33 %)
^{1}H NMR (400 MHZ, $CDCl_3$):	1.47 – 1.57 (2H, m, ArC(=O)-CH_2-CH_2-CH_2-CH_2-CH_2-C(=O)Ar), 1.83 (4H, quintet, $J_{H\text{-}H}$ = 7.2 Hz, Ar-C(=O)-CH_2-CH_2-CH_2-CH_2-CH_2-C(=O)Ar), 3.03 (4H, t, $J_{H\text{-}H}$ = 7.2 Hz, ArC(=O)-CH_2-$(CH_2)_3$-CH_2-C(=O)Ar), 7.65 – 7.76 (12H, m, Ar*H*), 8.05 (4H, d*, $J_{H\text{-}H}$ = 8.4 Hz, Ar*H*)
	*poorly defined
^{13}C{^{1}H} NMR (100.5 MHZ, $CDCl_3$):	23.93, 28.86, 38.40, 111.80, 118.12, 127.43, 127.87, 128.81, 132.70, 136.96, 143.34, 144.26, 199.58 (C=O)
FT-IR (ν max, cm-1):	509, 552, 579, 631, 695, 725, 806, 829, 849, 943, 970, 1001, 1034, 1132, 1181, 1205, 1246, 1289, 1321, 1360, 1397, 1448, 1493, 1521, 1603, 1678, 2225, 2937,
MS (ESI+, *m/z*):	505.1868 (calcd. for $C_{33}H_{26}N_2NaO_3$: 505.1886, M + Na)
Assay (RP- HPLC):	95.8 % (retention time 15.0 min)

1.3. Supplemental 1D 1H NOESY difference NMR spectra

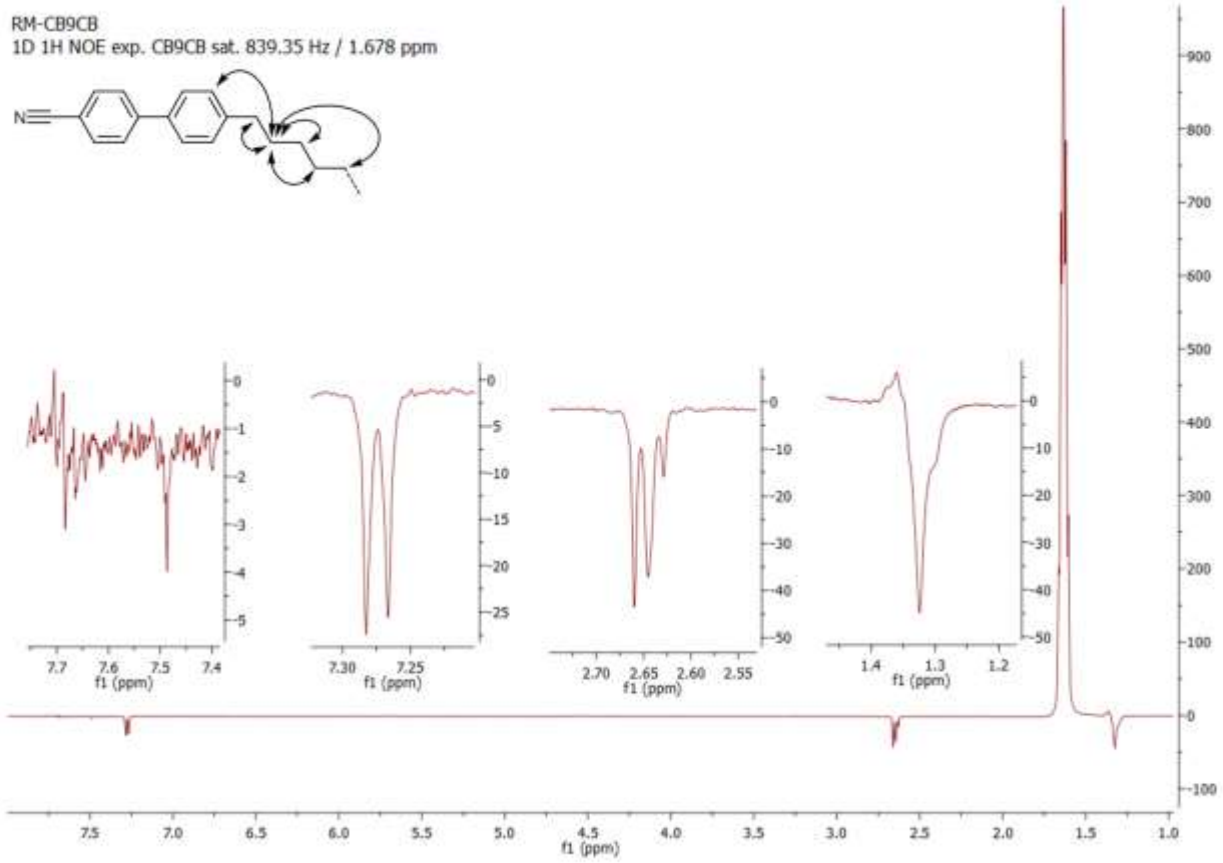

Figure SI1: 1D ^{1}H NOE spectra of CB9CB (**1**) saturated at 839.35 Hz (1.678 ppm). Black arrows indicate the assignment of observed NOE enhancements.

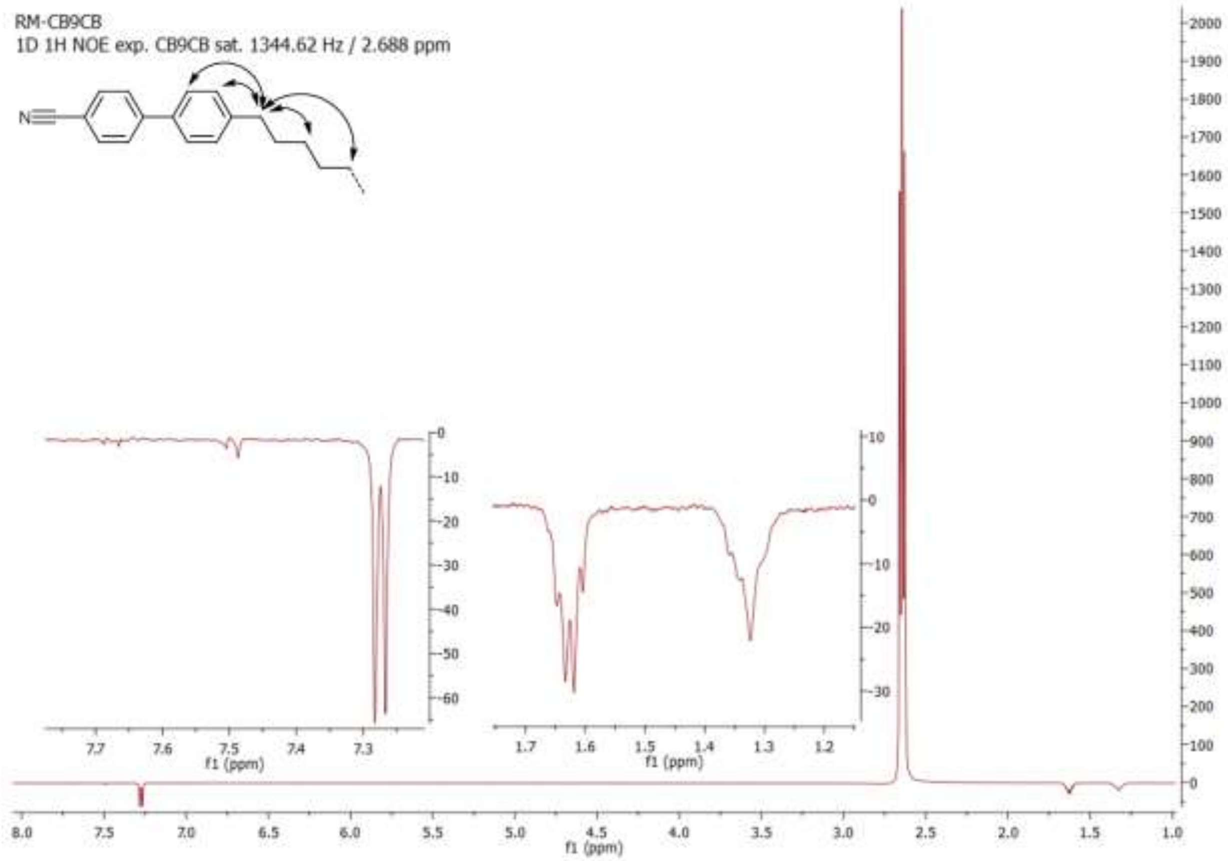

Figure SI2: 1D ^{1}H NOE spectra of CB9CB (**1**) saturated at 1344.62 Hz (2.688 ppm). Black arrows indicate the assignment of observed NOE enhancements.

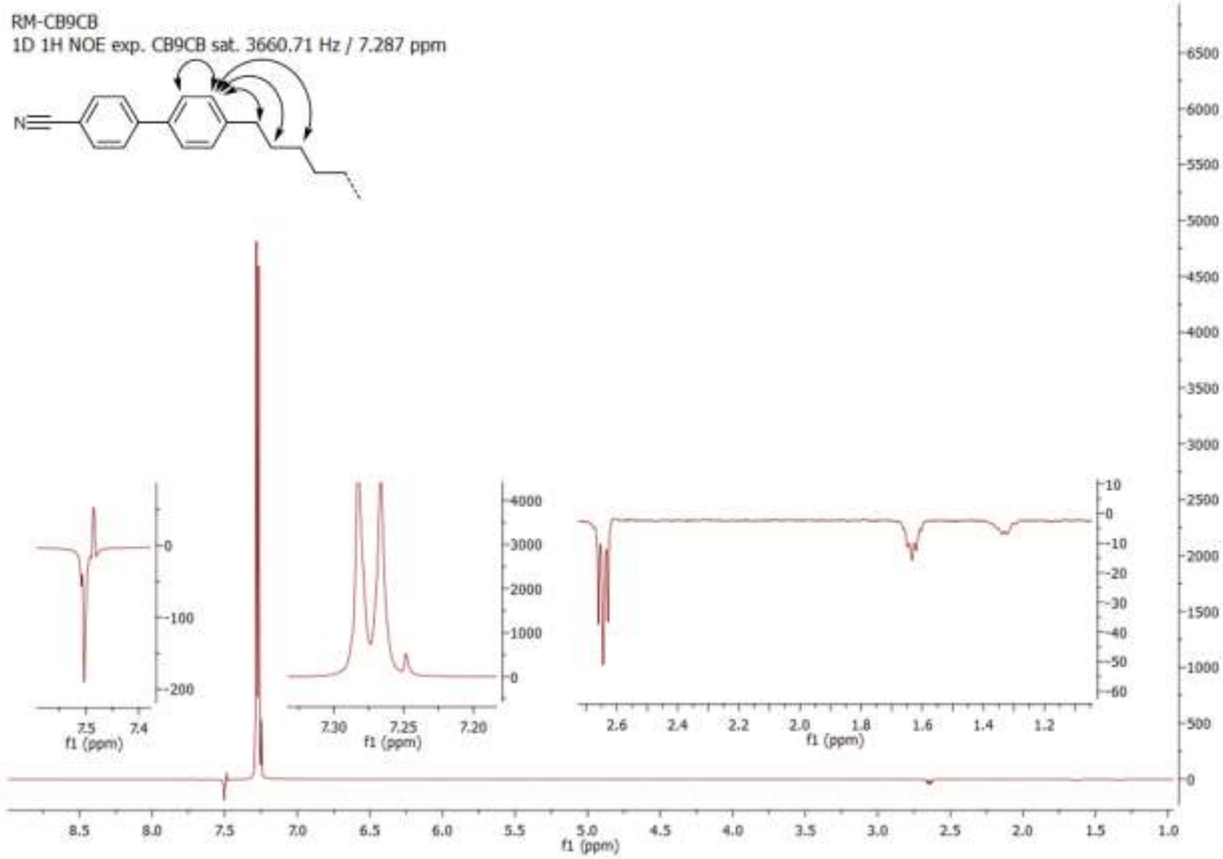

Figure SI3: 1D ^{1}H NOE spectra of CB9CB (**1**) saturated at 3660.71 Hz (7.287 ppm). Black arrows indicate the assignment of observed NOE enhancements.

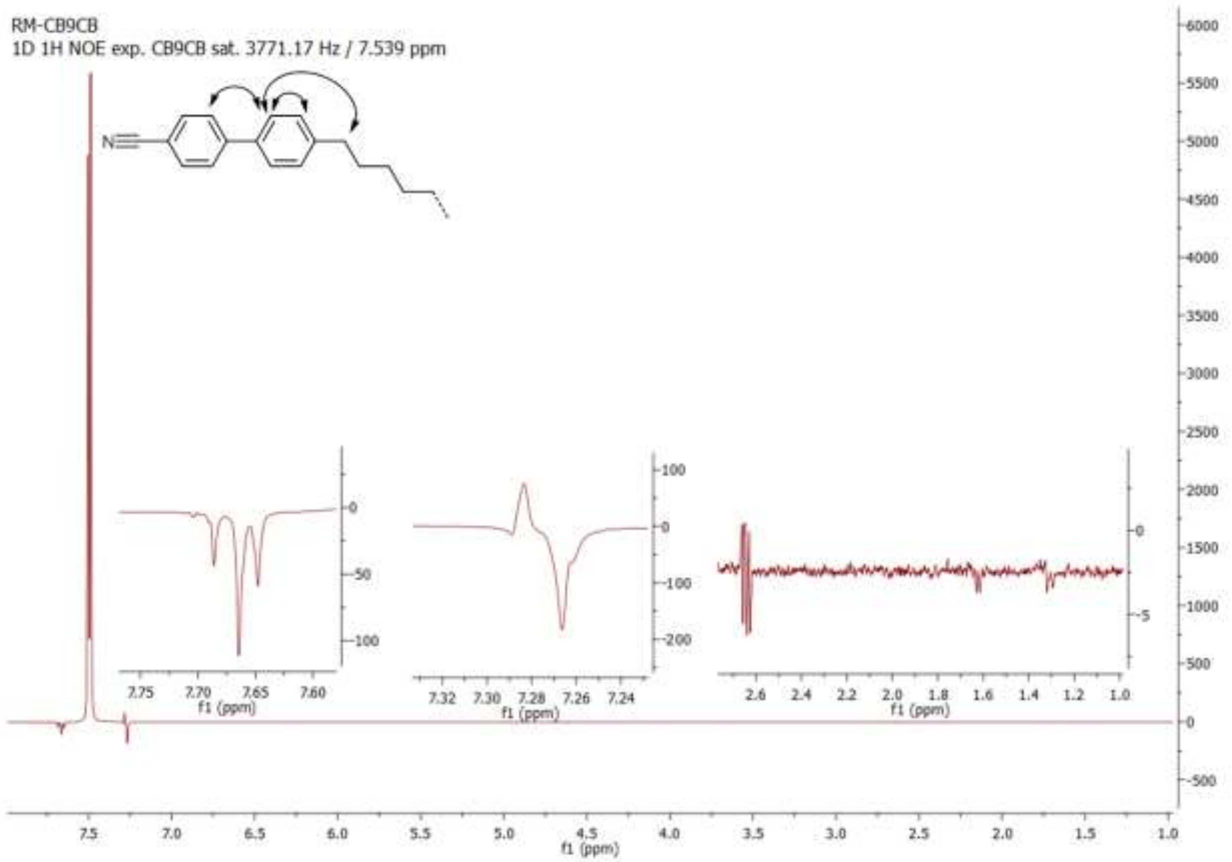

Figure SI4: 1D ^{1}H NOE spectra of CB9CB (**1**) saturated at 3771.17 Hz (7.539 ppm). Black arrows indicate the assignment of observed NOE enhancements.

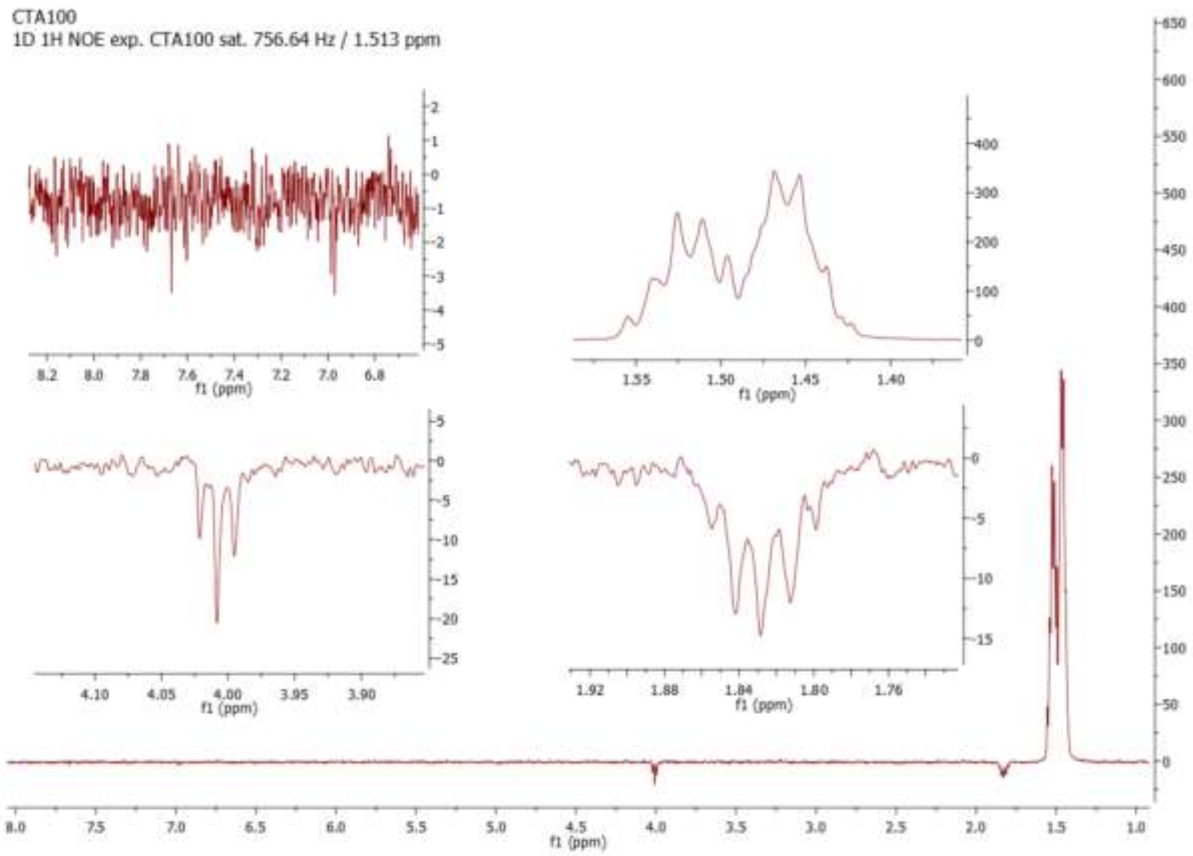

Figure SI6: 1D ^{1}H NOE spectra of CBO7OCB (**3**) saturated at 756.64 Hz (1.513). Multiple proton environments were saturated due to their close proximity, so no assignment of NOE enhancements were made based on this spectra.

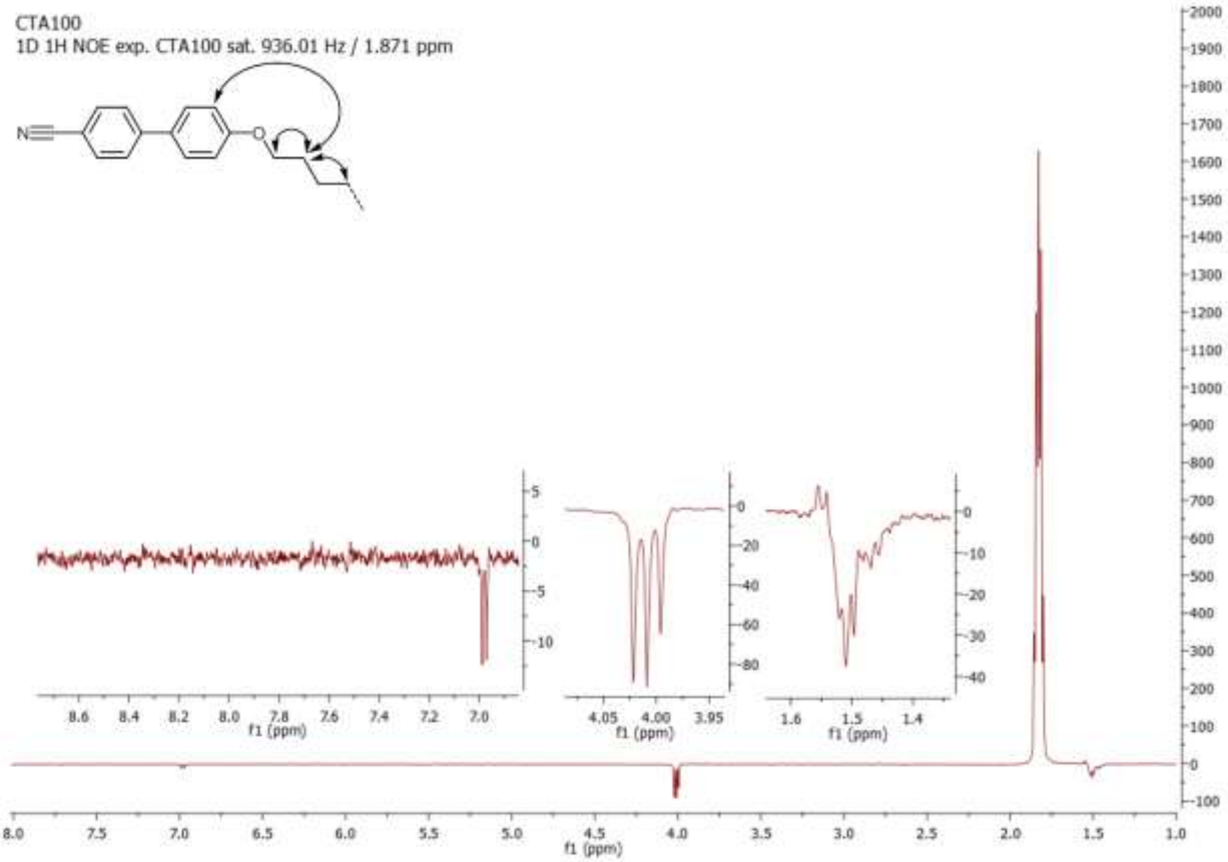

Figure SI6: 1D ^{1}H NOE spectra of CBO7OCB (**3**) saturated at 936.01 Hz (1.871ppm). Black arrows indicate the assignment of observed NOE enhancements.

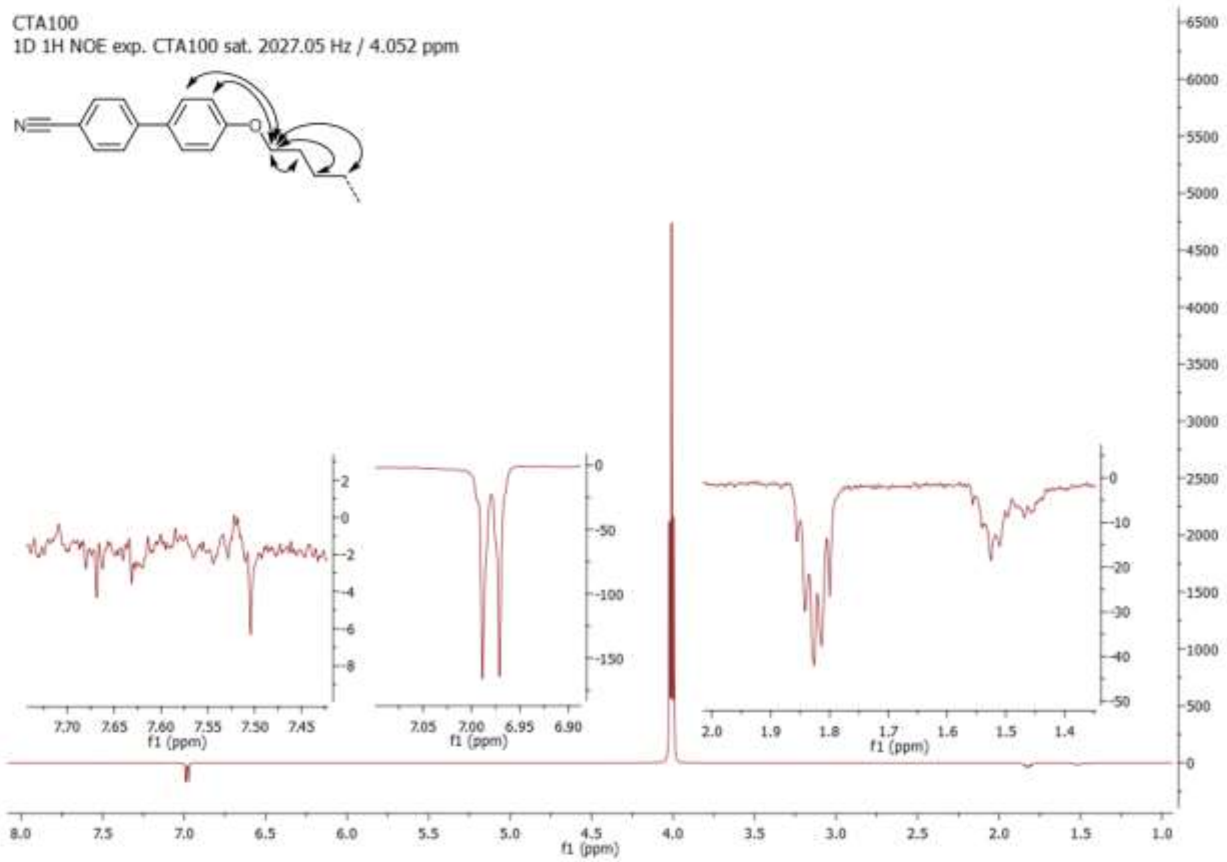

Figure SI7: 1D ^{1}H NOE spectra of CBO7OCB (**3**) saturated at 2027.05 Hz (4.052 ppm). Black arrows indicate the assignment of observed NOE enhancements.

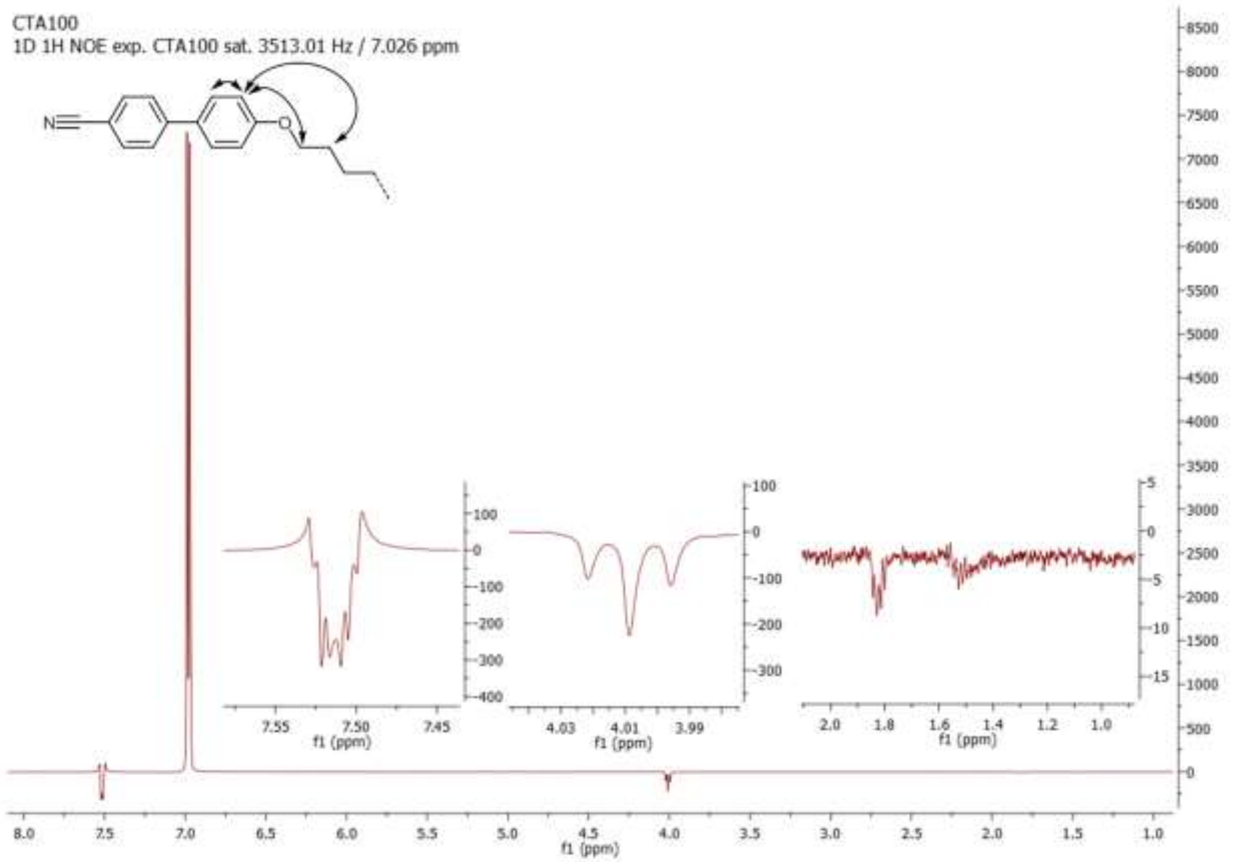

Figure SI8: 1D ^{1}H NOE spectra of CBO7OCB (**3**) saturated at 3513.01 Hz (7.026 ppm). Black arrows indicate the assignment of observed NOE enhancements.

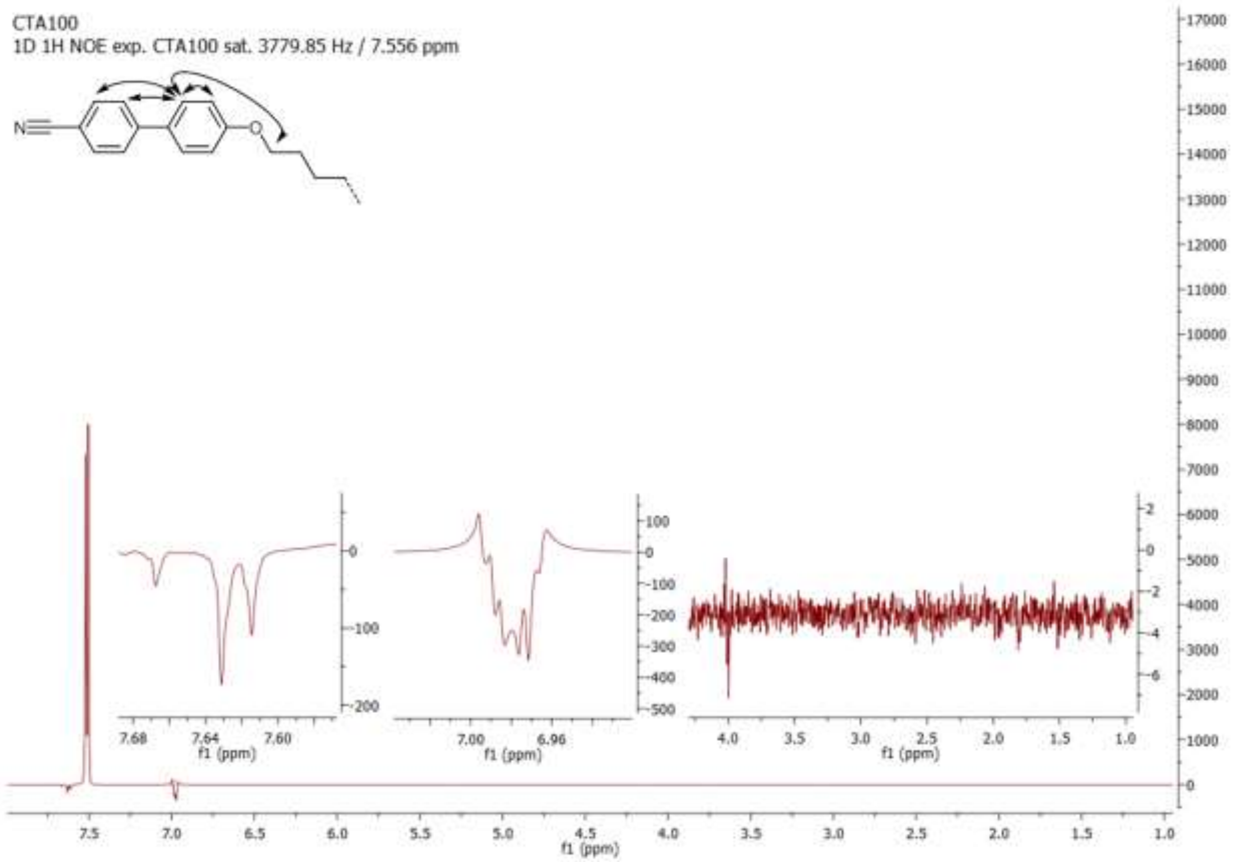

Figure SI9: 1D ^{1}H NOE spectra of CBO7OCB (**3**) saturated at 3779.85 Hz (7.556 ppm). Black arrows indicate the assignment of observed NOE enhancements.

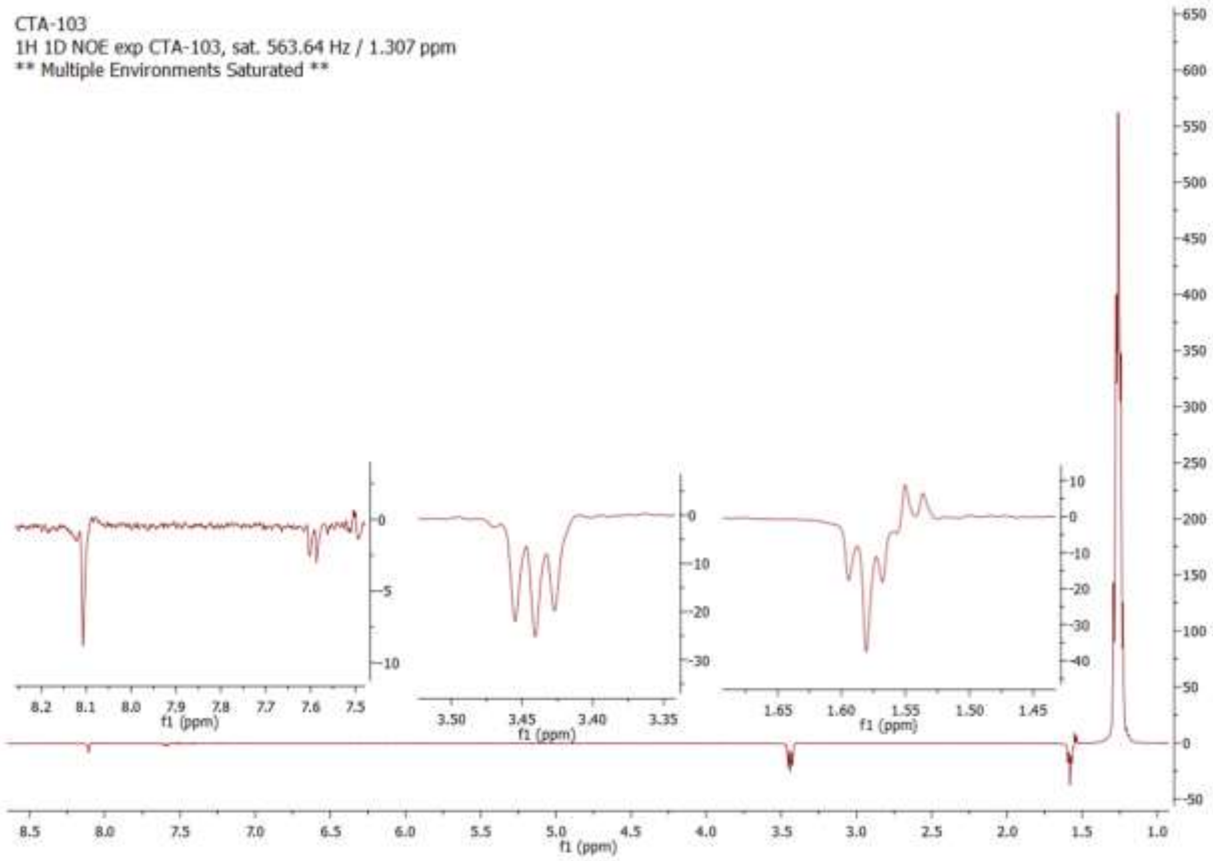

Figure SI9: 1D ¹H NOE spectra of **2** saturated at 563.64 Hz (1.307). Multiple proton environments were saturated due to their close proximity, so no assignment of NOE enhancements were made based on this spectra.

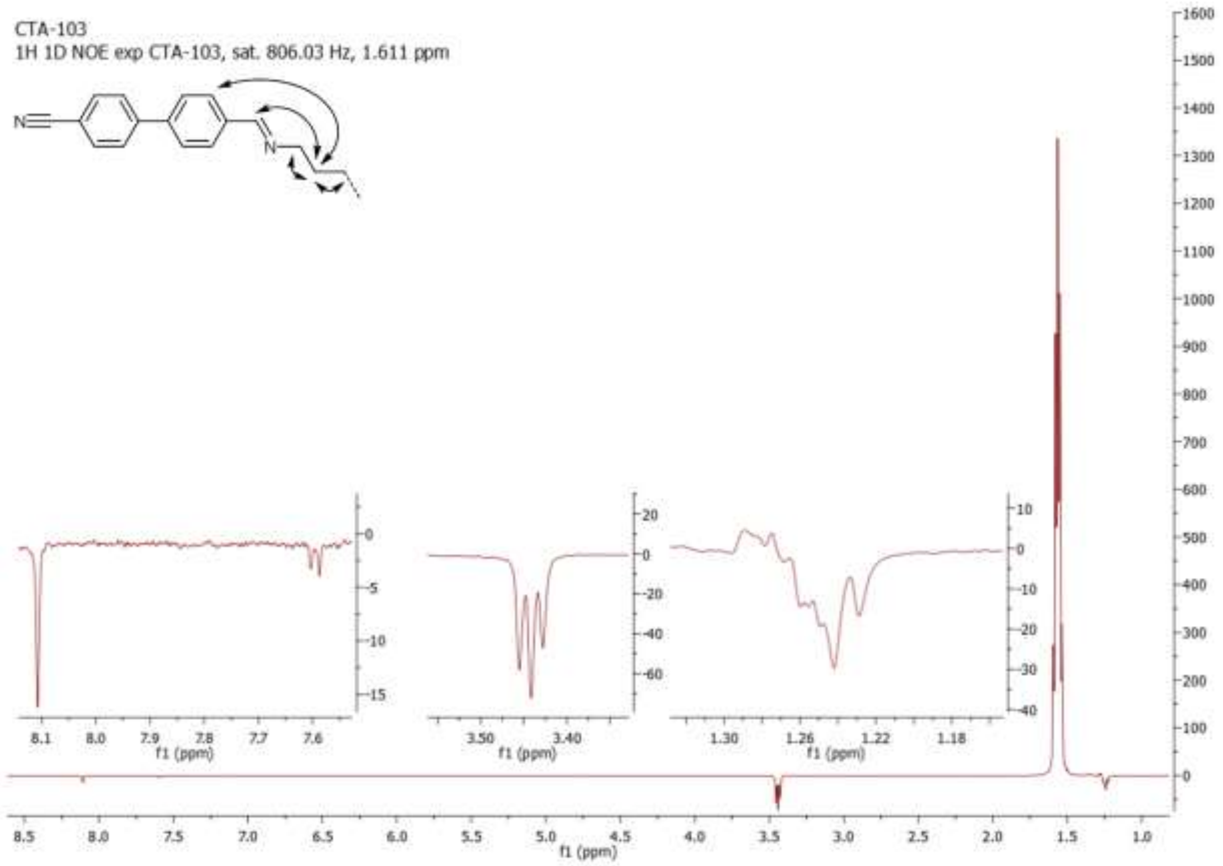

Figure SI10: 1D ^{1}H NOE spectra of **2** saturated at 806.03 Hz (1.611 ppm). Black arrows indicate the assignment of observed NOE enhancements.

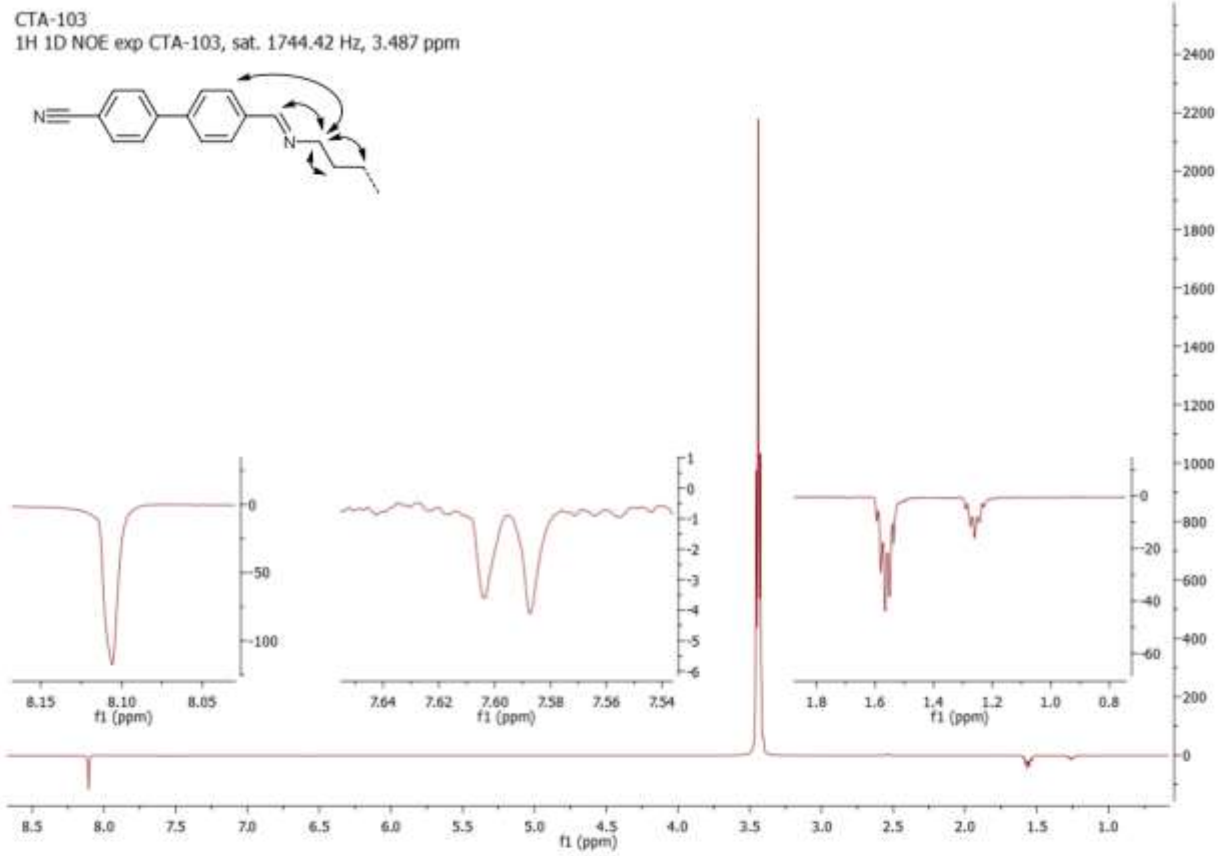

Figure SI11: 1D ^{1}H NOE spectra of **2** saturated at 1744.47 Hz (3.487 ppm). Black arrows indicate the assignment of observed NOE enhancements.

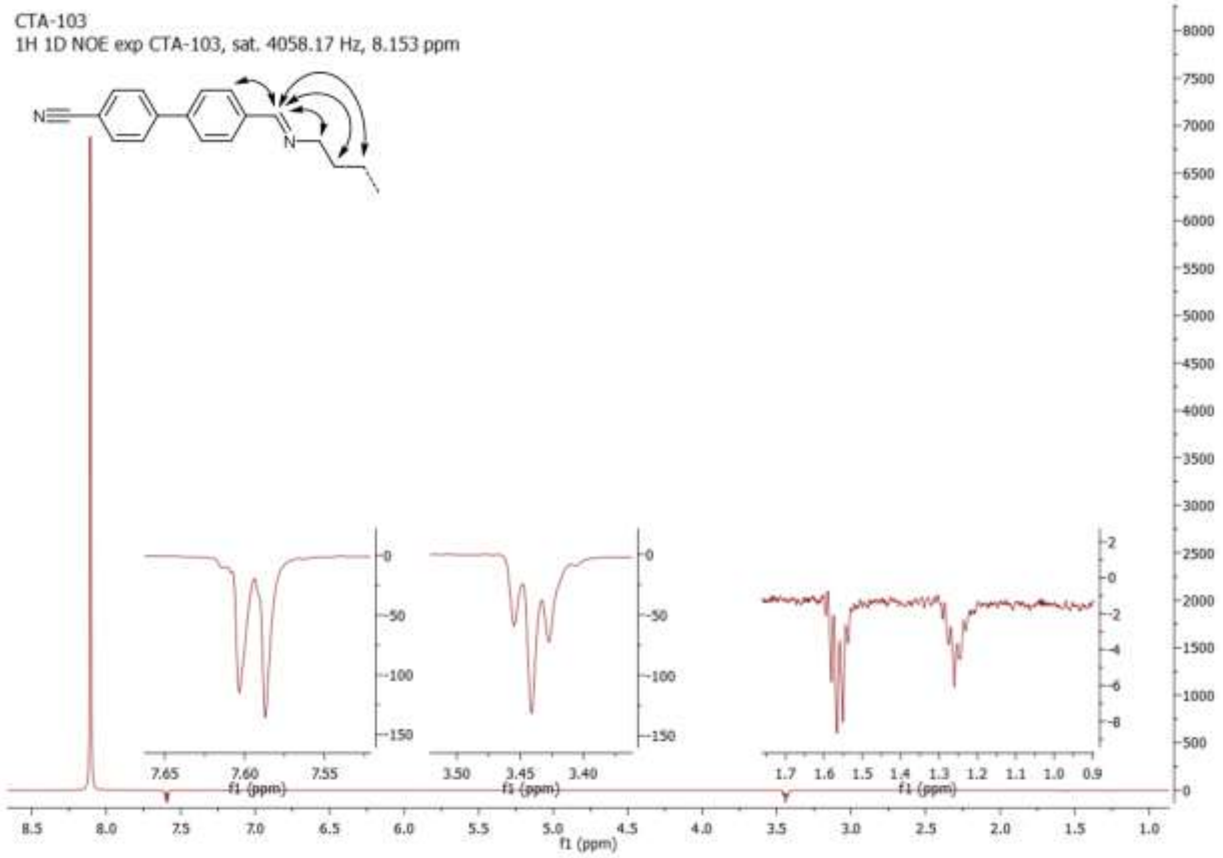

Figure SI12: 1D ^{1}H NOE spectra of **2** saturated at 4058.17 Hz (8.153 ppm). Black arrows indicate the assignment of observed NOE enhancements.

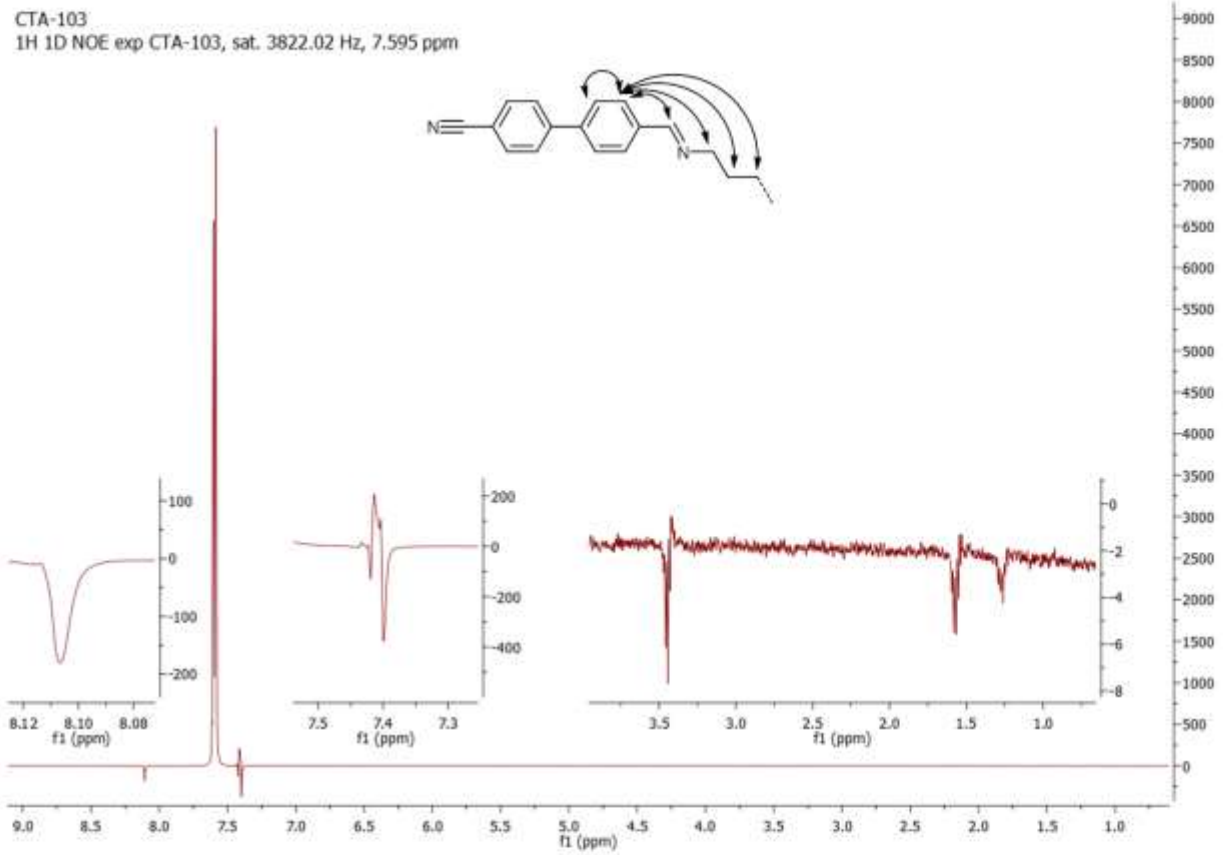

Figure SI13: 1D ^{1}H NOE spectra of **2** saturated at 3822.02 Hz (7.595 ppm). Black arrows indicate the assignment of observed NOE enhancements.

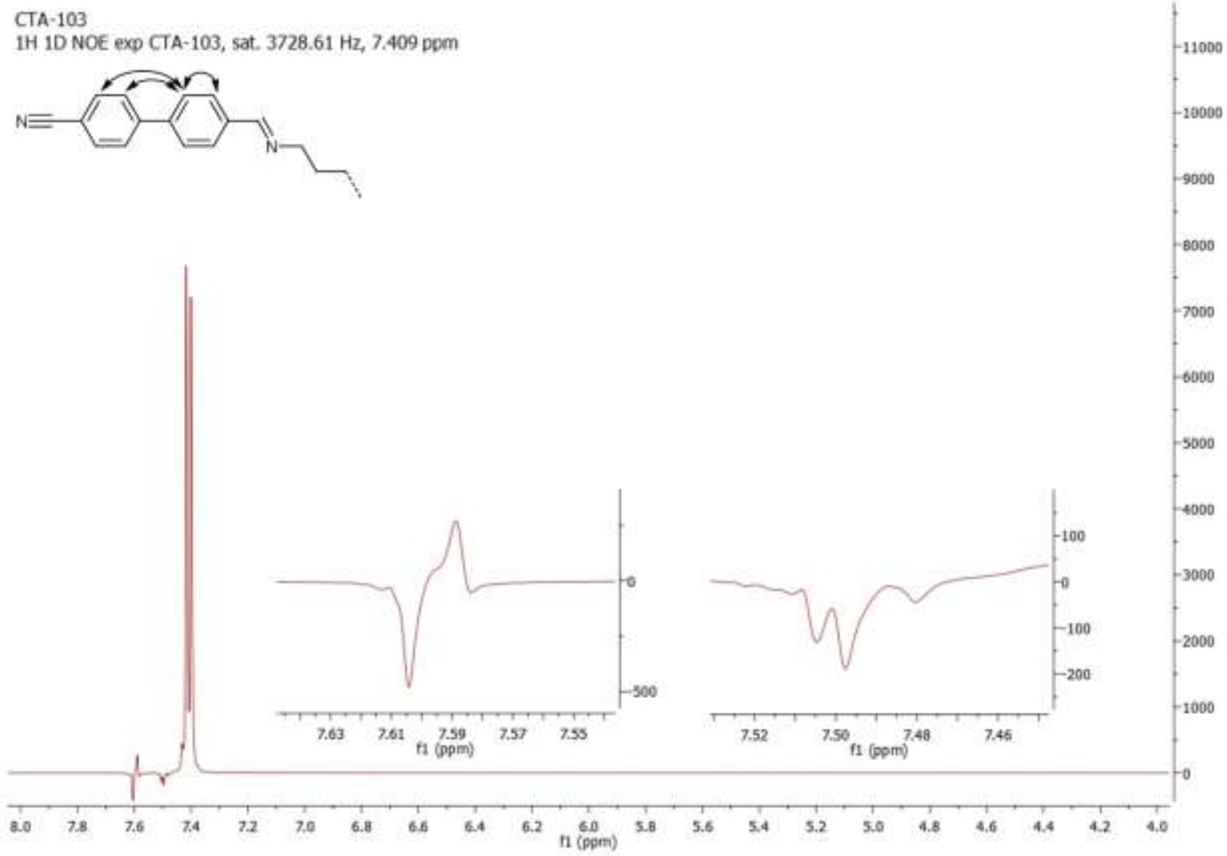

Figure SI14: 1D ^{1}H NOE spectra of **2** saturated at 3728.61 Hz (7.408 ppm). Black arrows indicate the assignment of observed NOE enhancements.

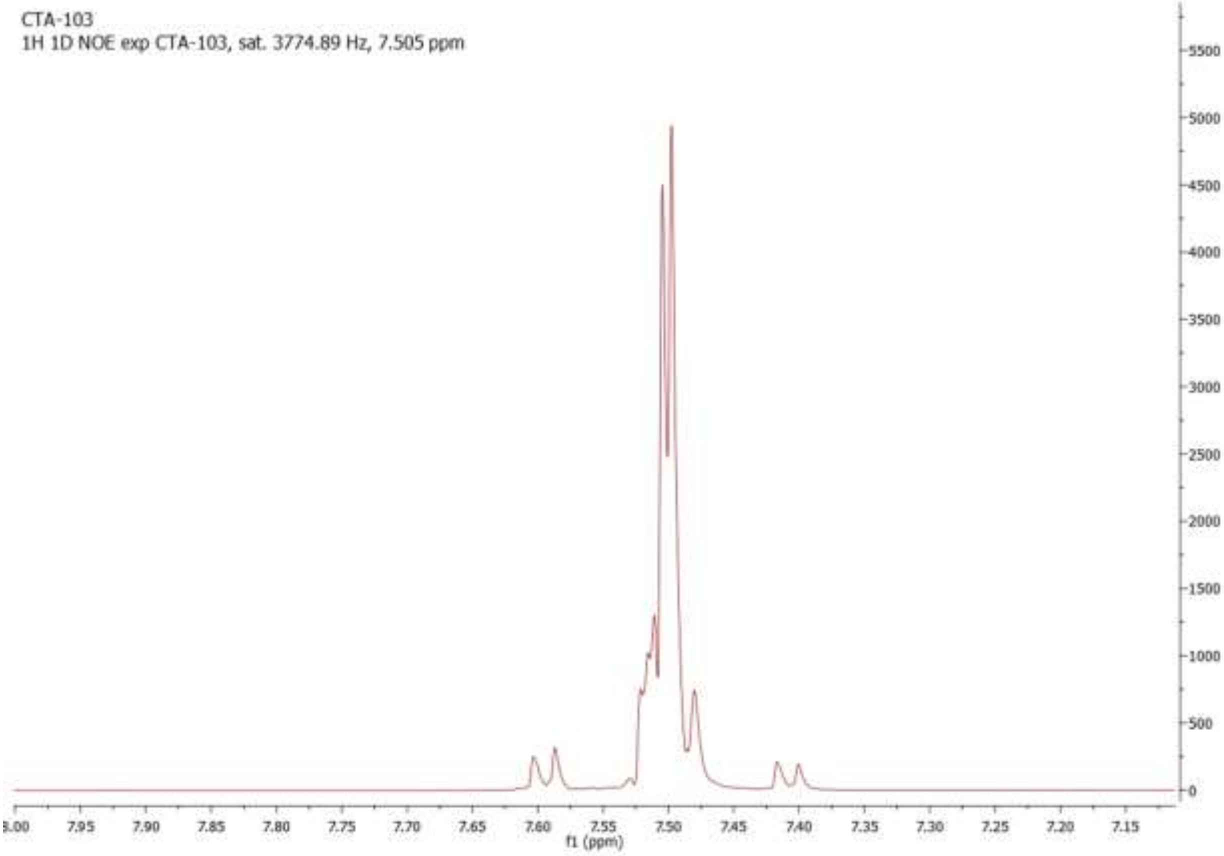

Figure SI15: 1D ^{1}H NOE spectra of **2** saturated at 3774.89 Hz (7.505). Multiple aromatic proton environments were saturated due to their close proximity, so no assignment of NOE enhancements could be made based on this spectra.

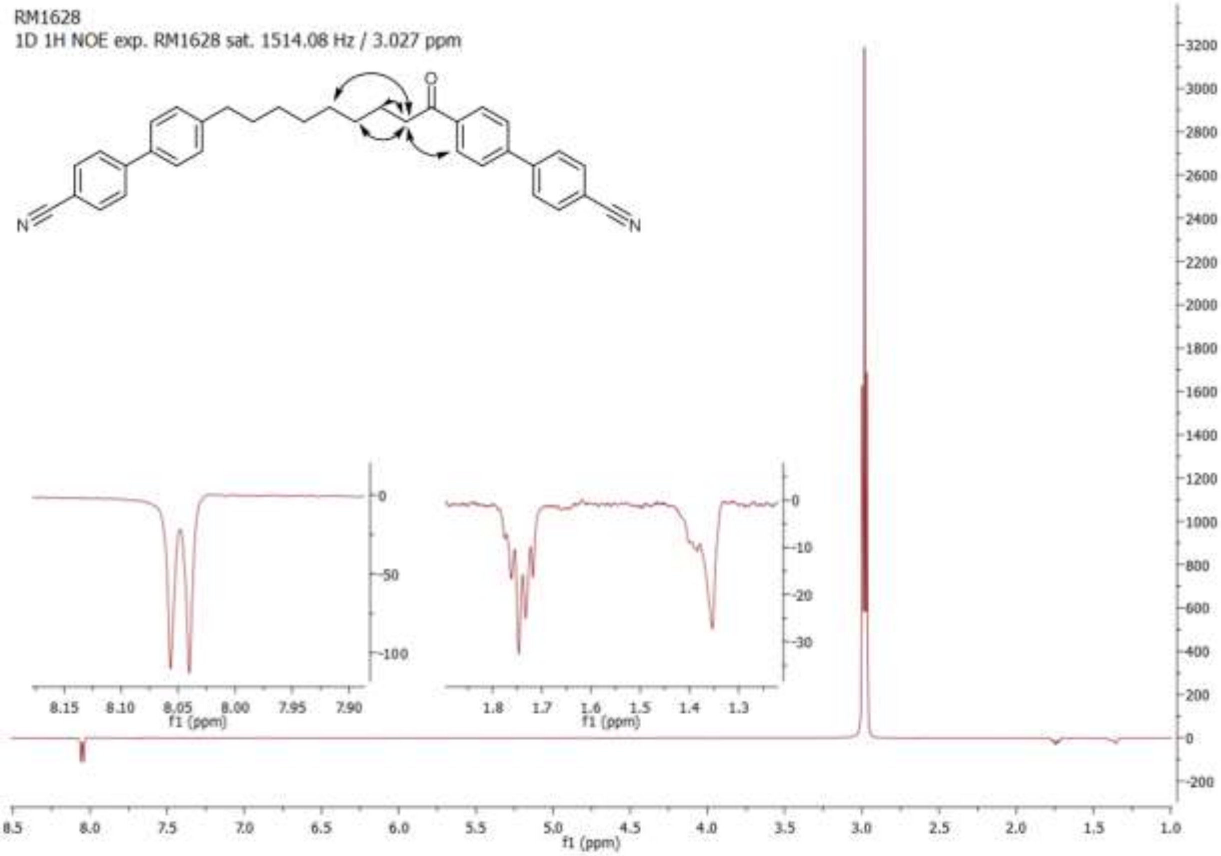

Figure SI16: 1D ^{1}H NOE spectra of **6** saturated at 1514.08 Hz (3.027 ppm). Black arrows indicate the assignment of observed NOE enhancements.

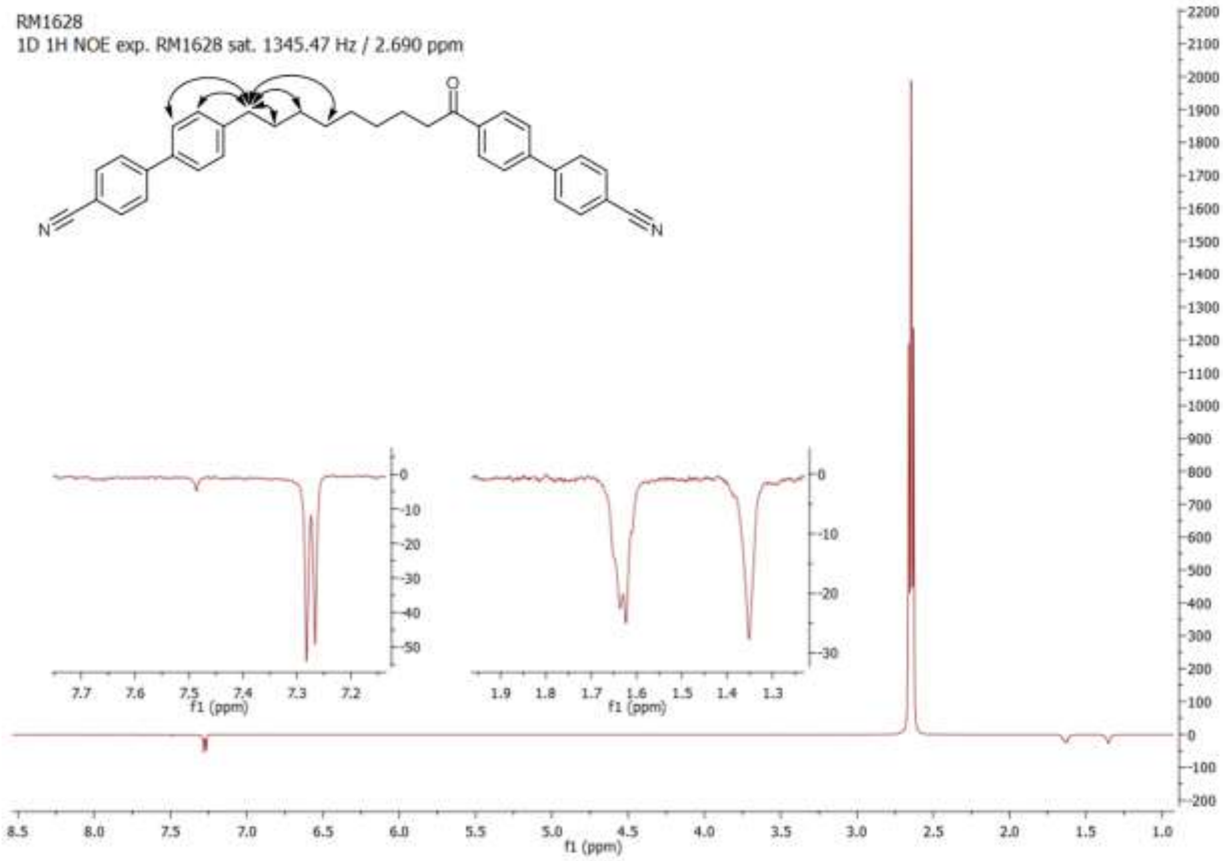

Figure SI17: 1D ^{1}H NOE spectra of **6** saturated at 1345.47 Hz (2.690 ppm). Black arrows indicate the assignment of observed NOE enhancements.

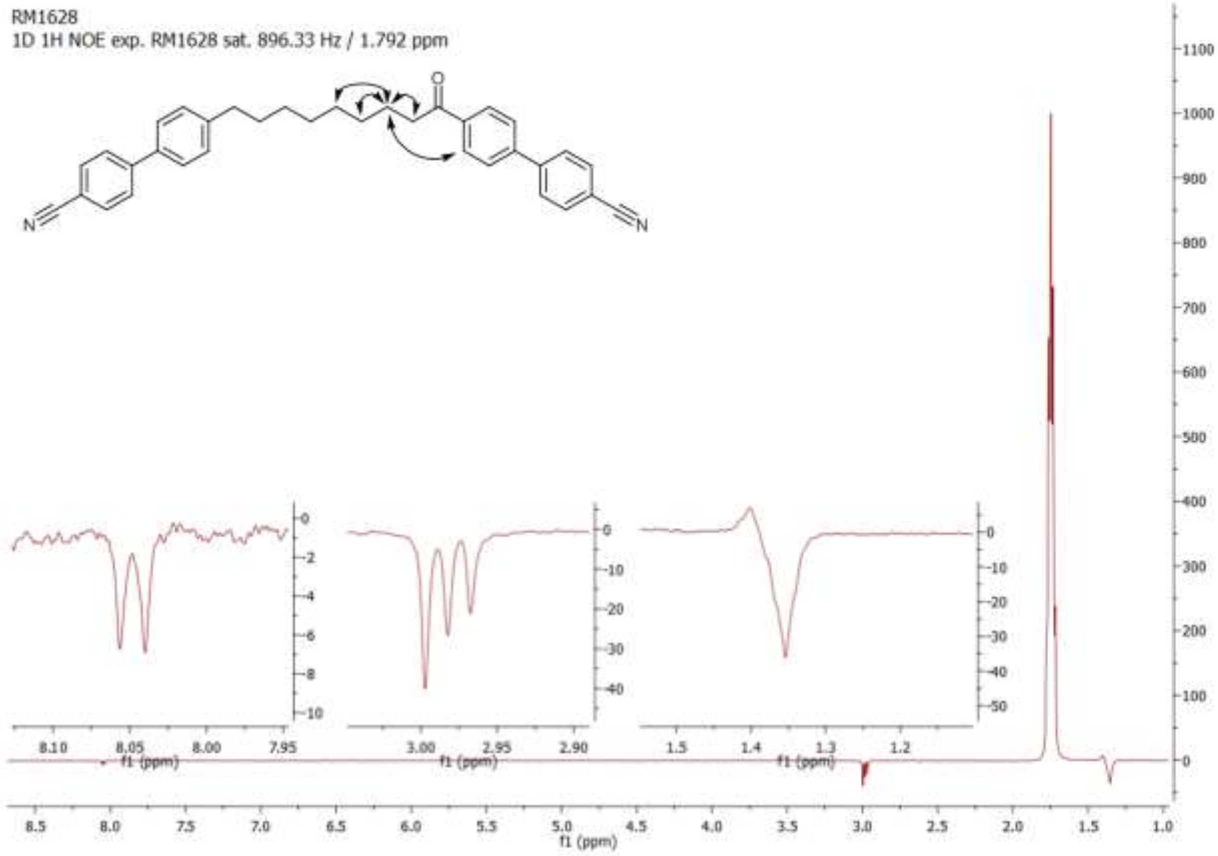

Figure SI17: 1D ^{1}H NOE spectra of **6** saturated at 896.33 Hz (1.792 ppm). Black arrows indicate the assignment of observed NOE enhancements.

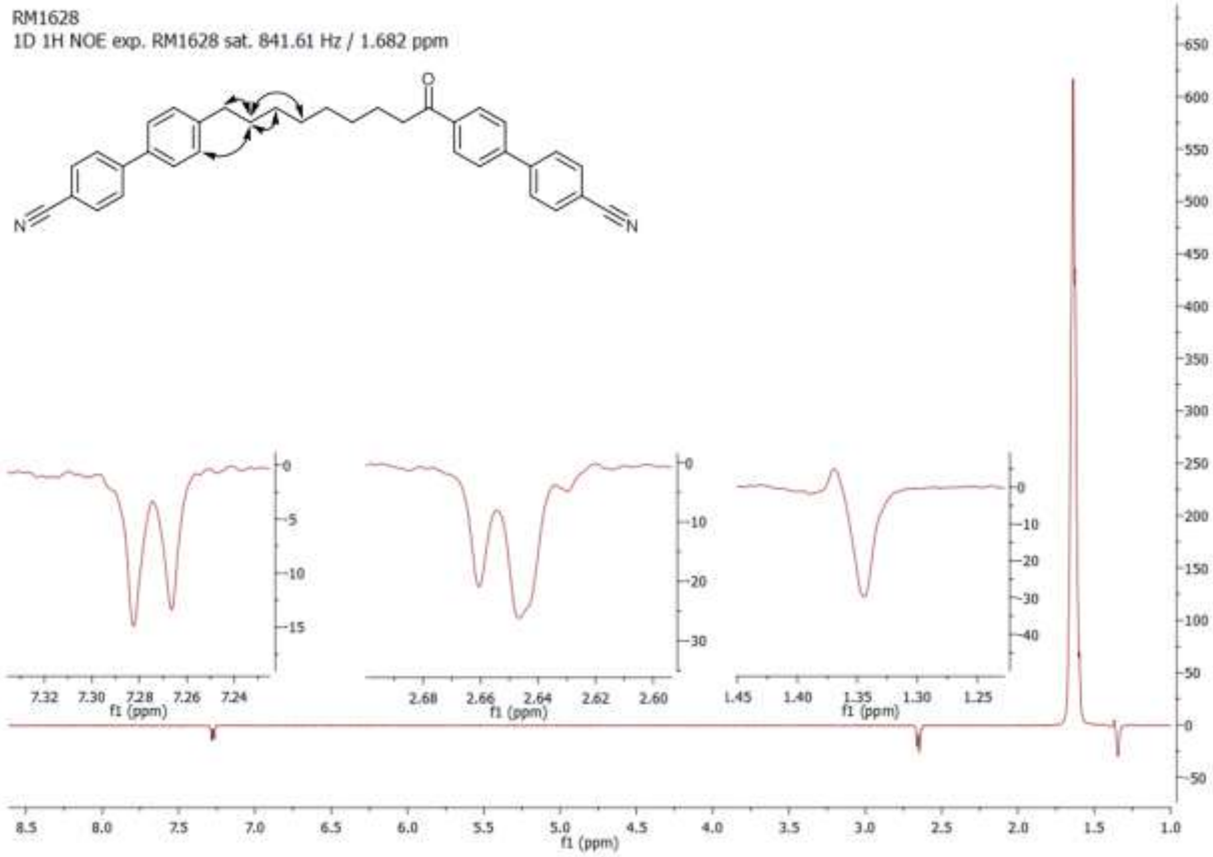

Figure SI18: 1D ^{1}H NOE spectra of **6** saturated at 841.61 Hz (1.682 ppm). Black arrows indicate the assignment of observed NOE enhancements.

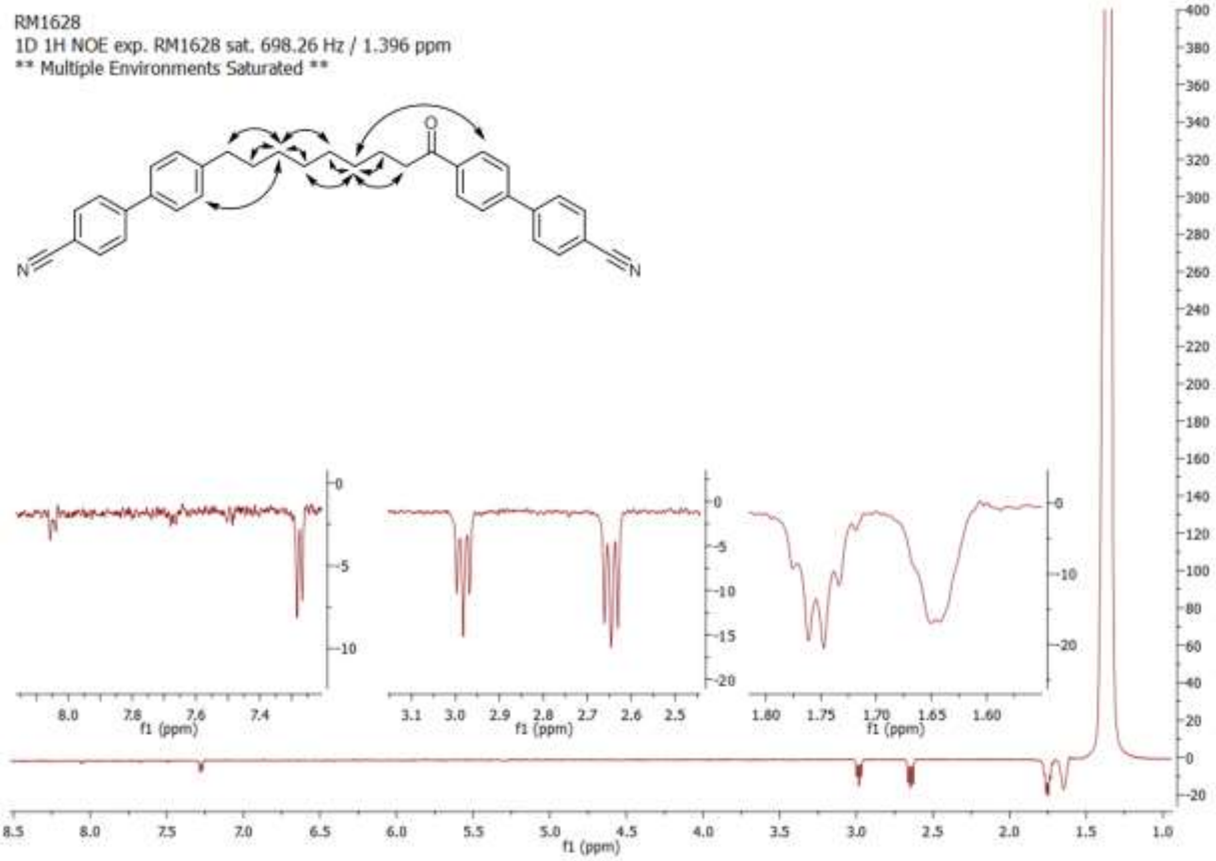

Figure SI19: 1D ^{1}H NOE spectra of **6** saturated at 698.26 Hz (1.396). While multiple proton environments were saturated (The C3 and C6 methylene units of the spacer) this did not prevent the assignments of some NOE enhancements.

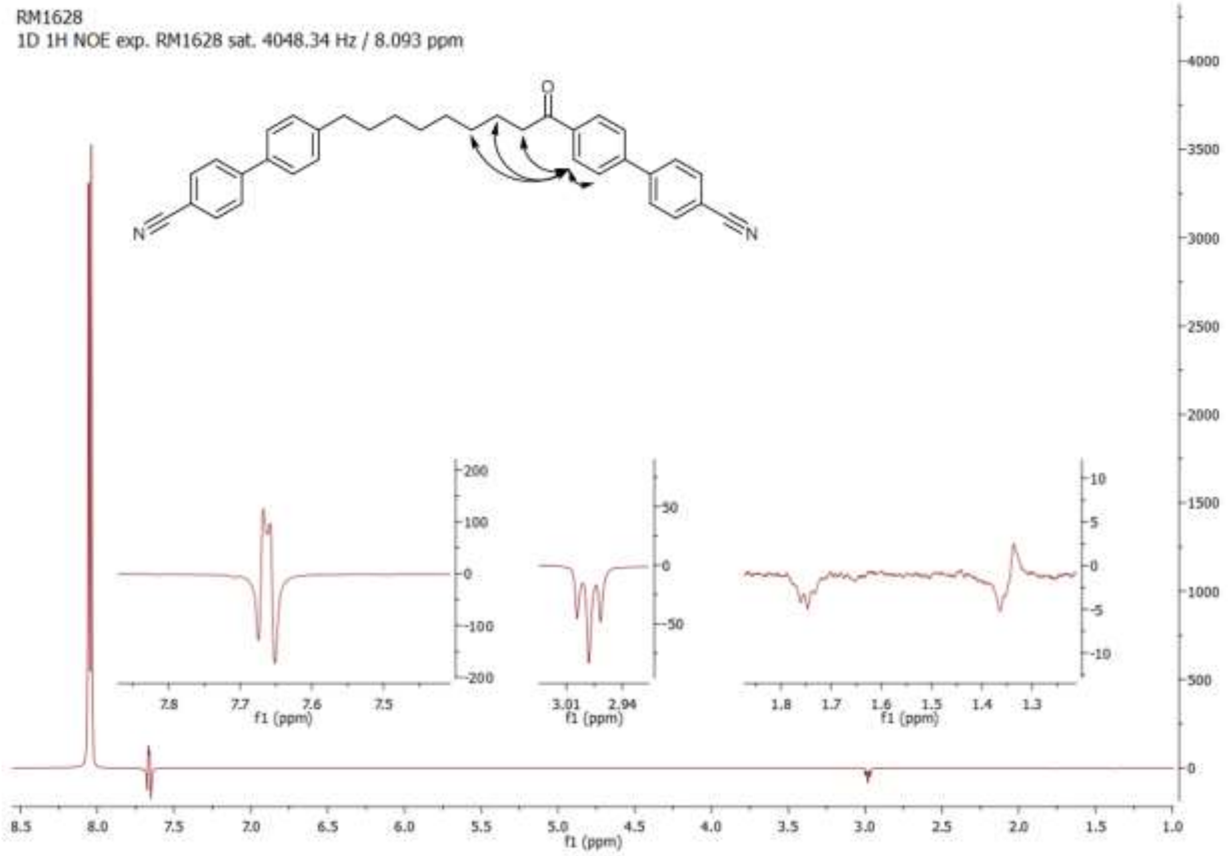

Figure SI20: 1D ^{1}H NOE spectra of **6** saturated at 4048.34 Hz (8.093 ppm). Black arrows indicate the assignment of observed NOE enhancements.

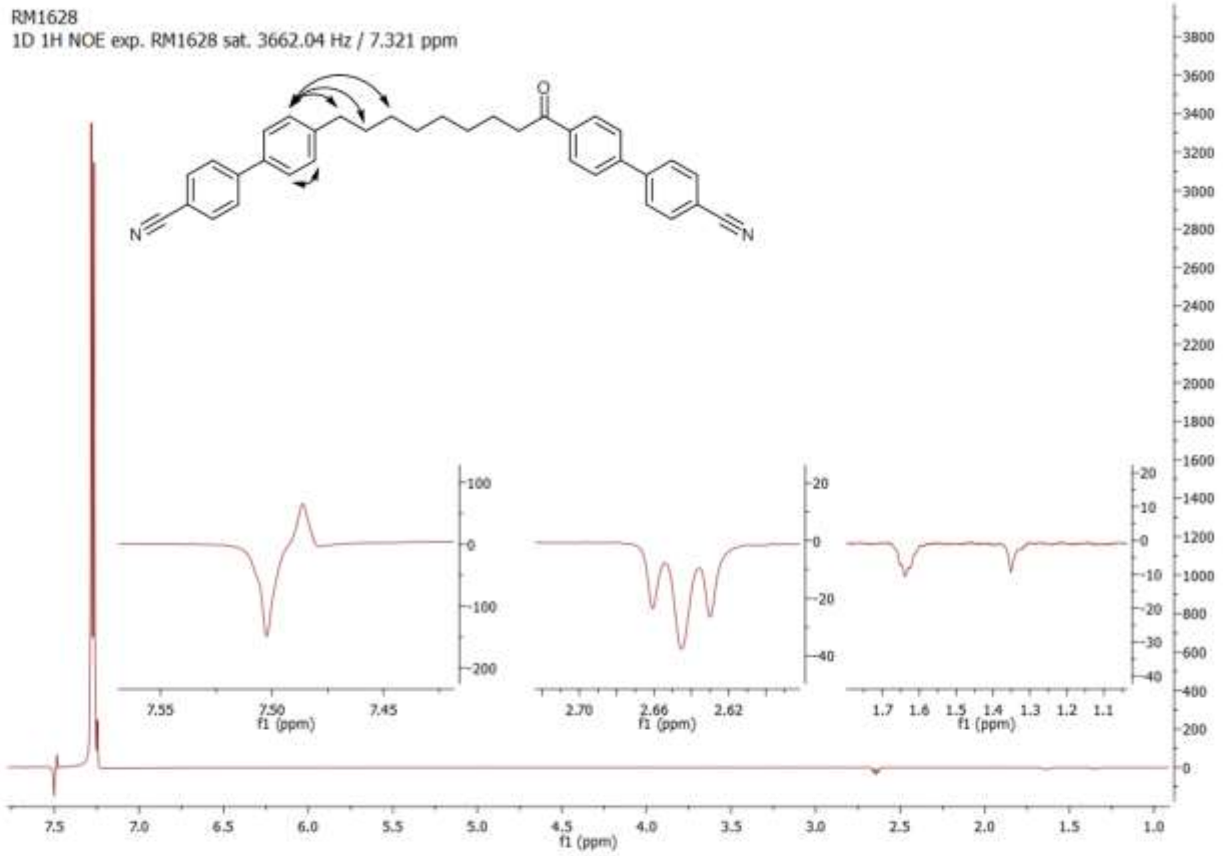

Figure SI21: 1D ^{1}H NOE spectra of **6** saturated at 3662.04 Hz (7.321 ppm). Black arrows indicate the assignment of observed NOE enhancements.

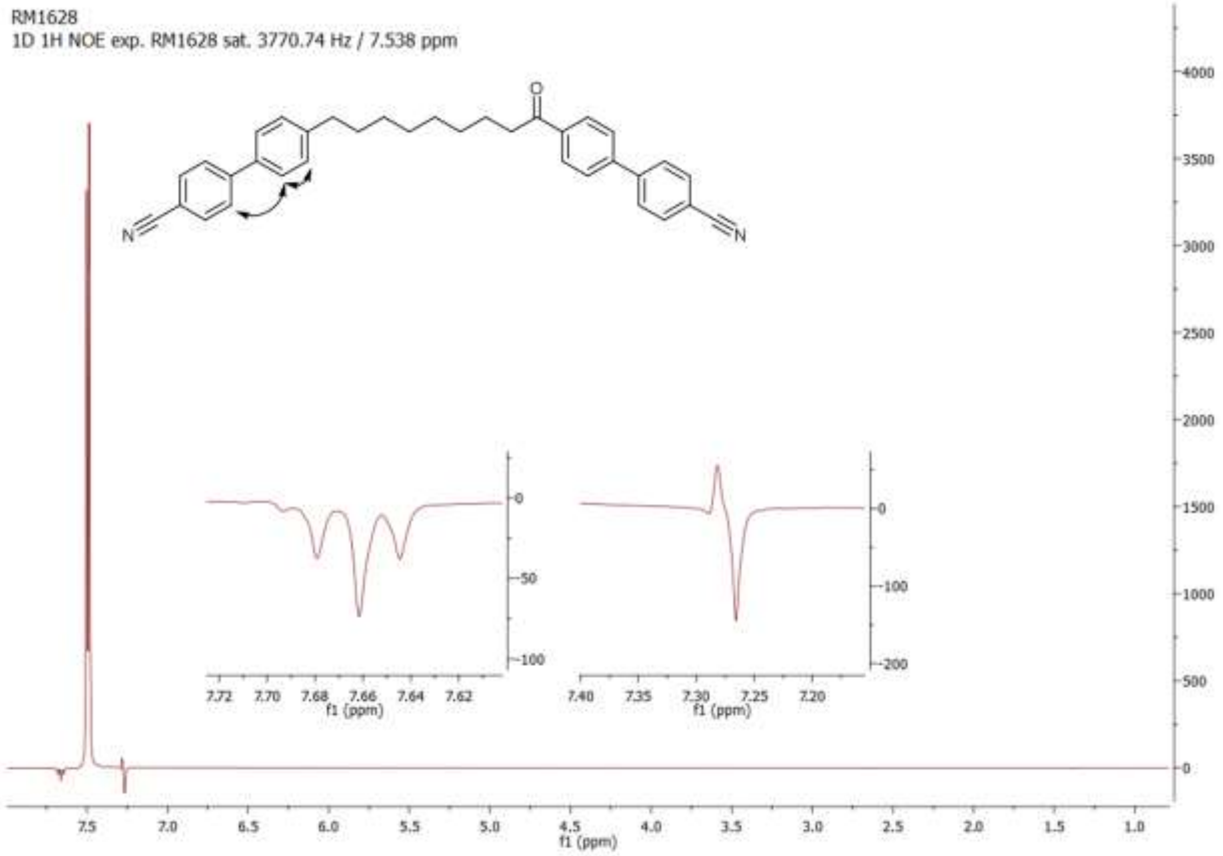

Figure SI22: 1D ^1H NOE spectra of **6** saturated at 3770.74 Hz (7.538 ppm). Black arrows indicate the assignment of observed NOE enhancements.

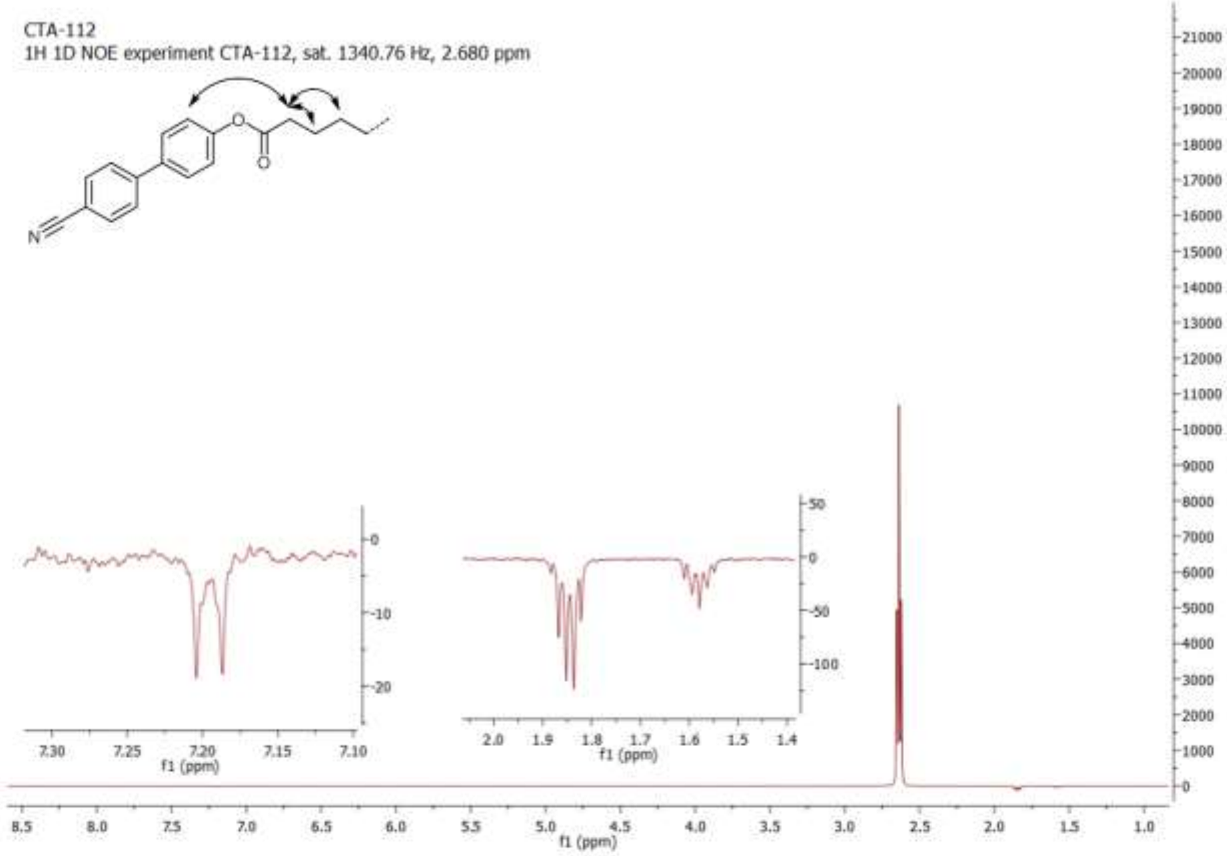

Figure SI23: 1D ^{1}H NOE spectra of **4** saturated at 1340.76 Hz (2.680 ppm). Black arrows indicate the assignment of observed NOE enhancements.

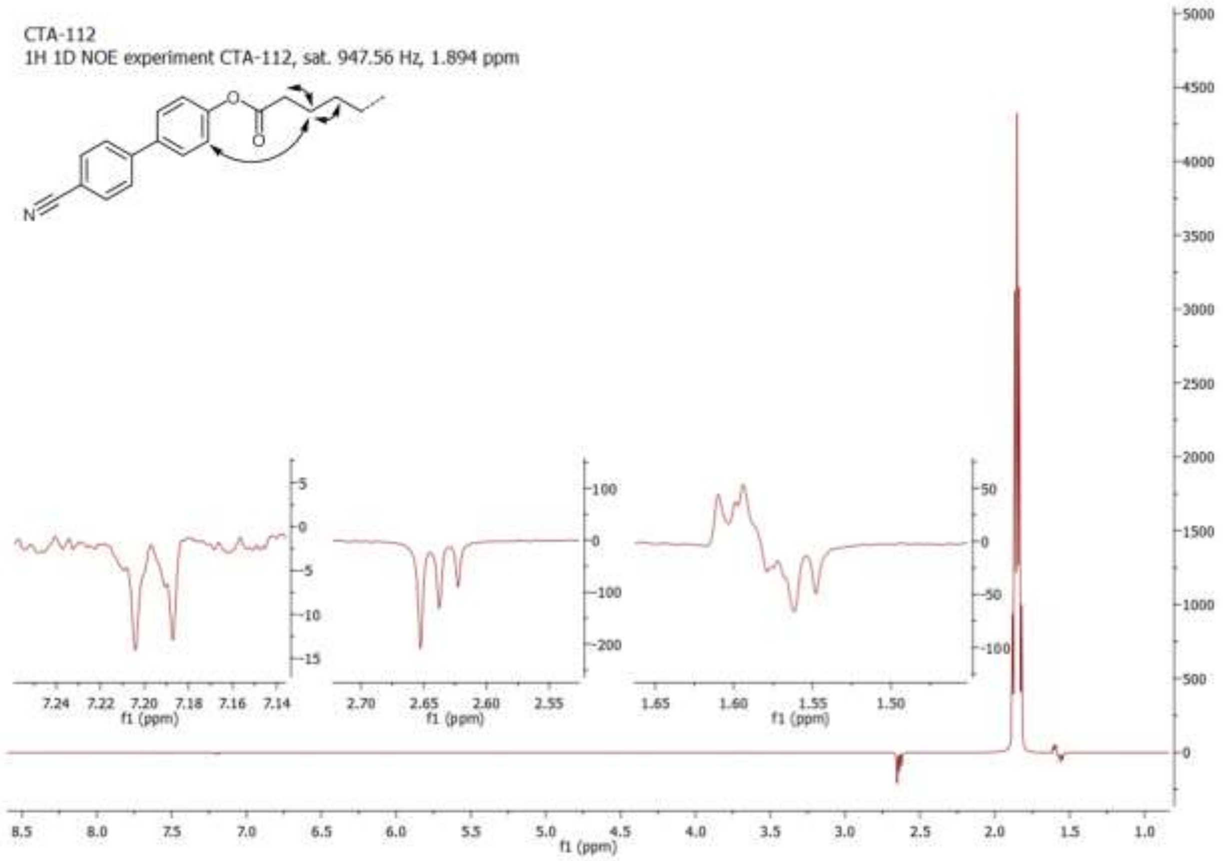

Figure SI24: 1D ^{1}H NOE spectra of **4** saturated at 947.56 Hz (1.894 ppm). Black arrows indicate the assignment of observed NOE enhancements.

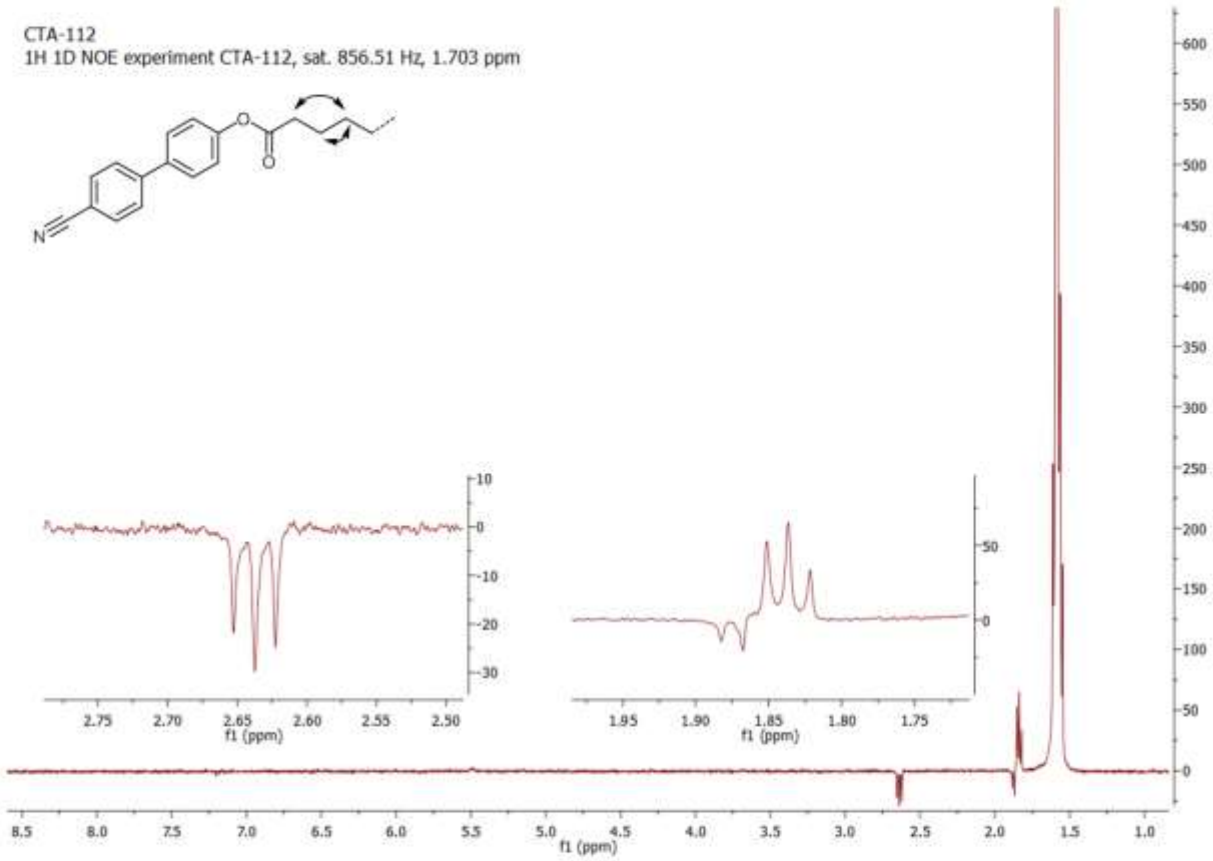

Figure SI25: 1D ^{1}H NOE spectra of **4** saturated at 856.51 Hz (1.703 ppm). Black arrows indicate the assignment of observed NOE enhancements.

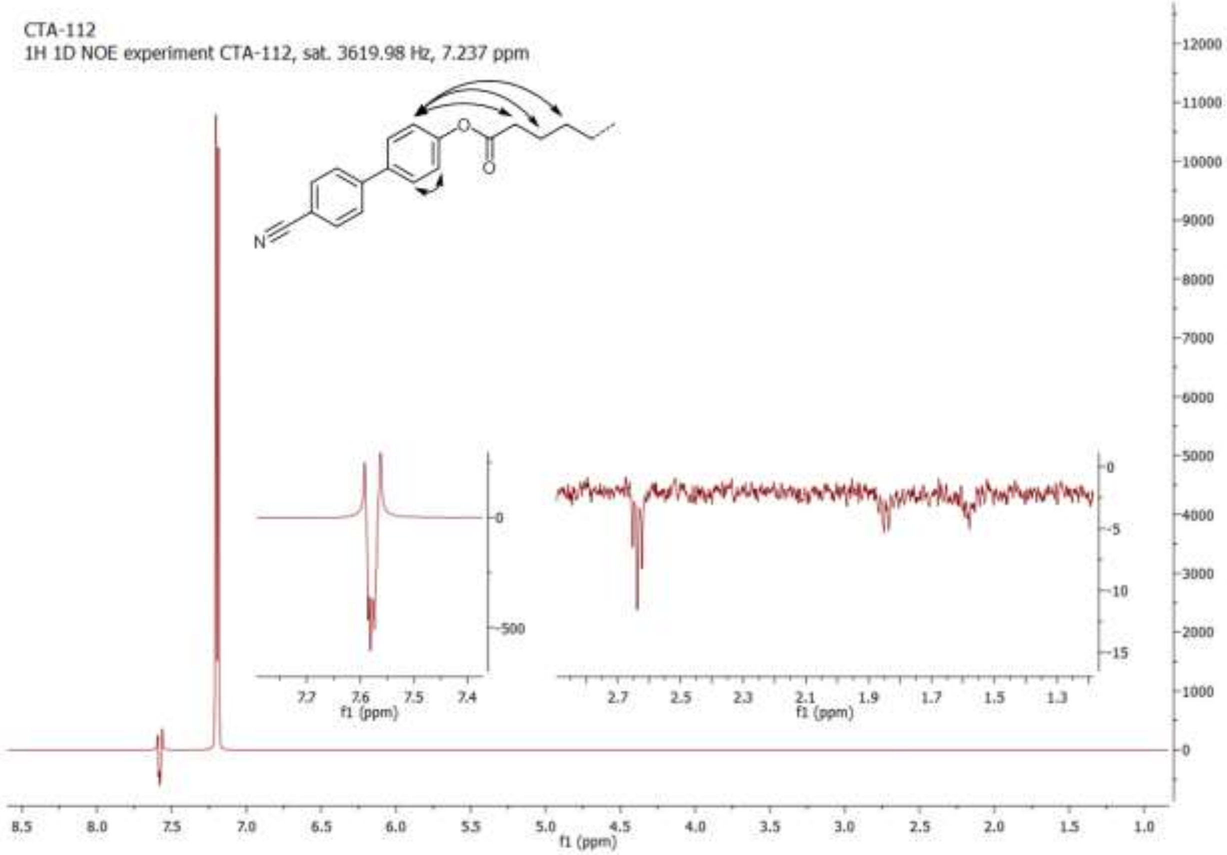

Figure SI26: 1D ^{1}H NOE spectra of **4** saturated at 3619.98 Hz (7.237 ppm). Black arrows indicate the assignment of observed NOE enhancements.

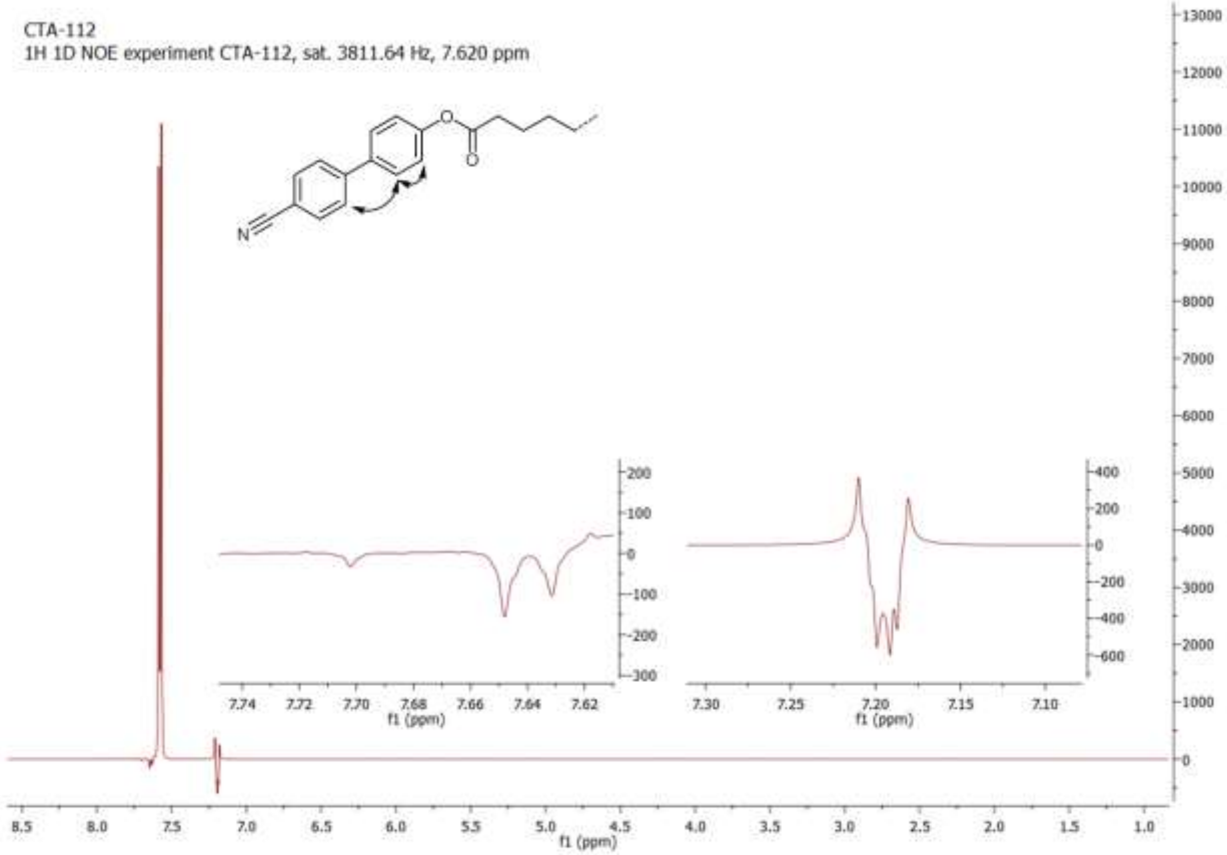

Figure SI27: 1D ^{1}H NOE spectra of **4** saturated at 3811.64 Hz (7.620 ppm). Black arrows indicate the assignment of observed NOE enhancements.

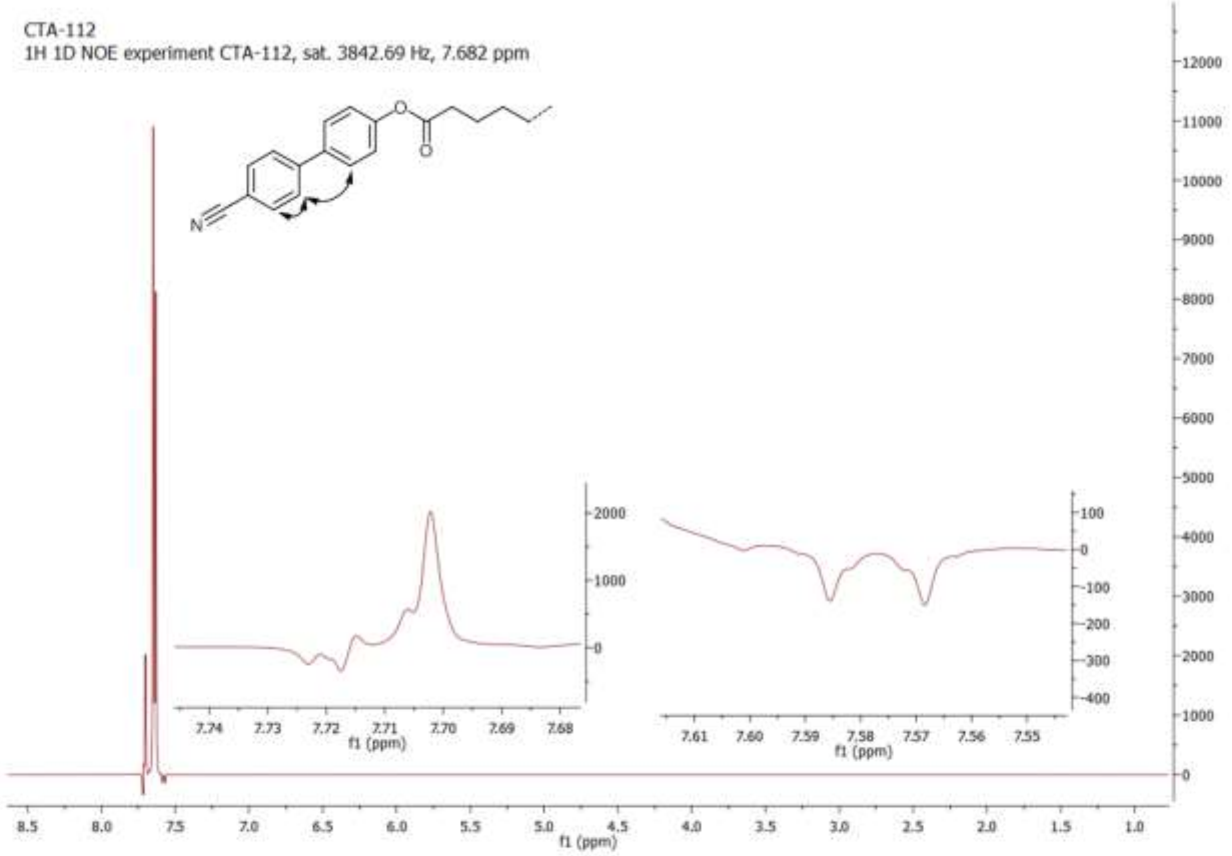

Figure SI28: 1D ^{1}H NOE spectra of **4** saturated at 3842.69 Hz (7.682 ppm). Black arrows indicate the assignment of observed NOE enhancements.

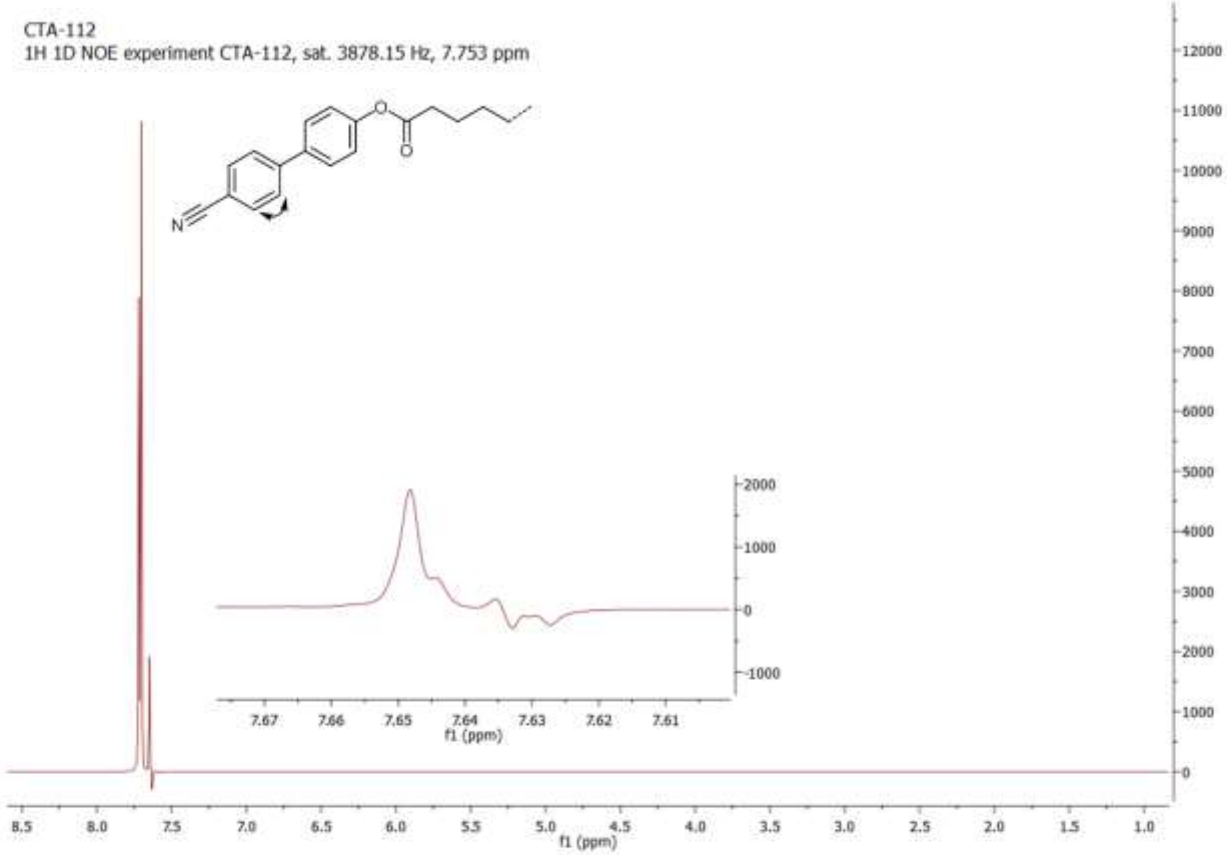

Figure SI29: 1D ^{1}H NOE spectra of **4** saturated at 3878.15 Hz (7.753 ppm). Black arrows indicate the assignment of observed NOE enhancements.

1.4. Supplemental Dihedral Calculations.

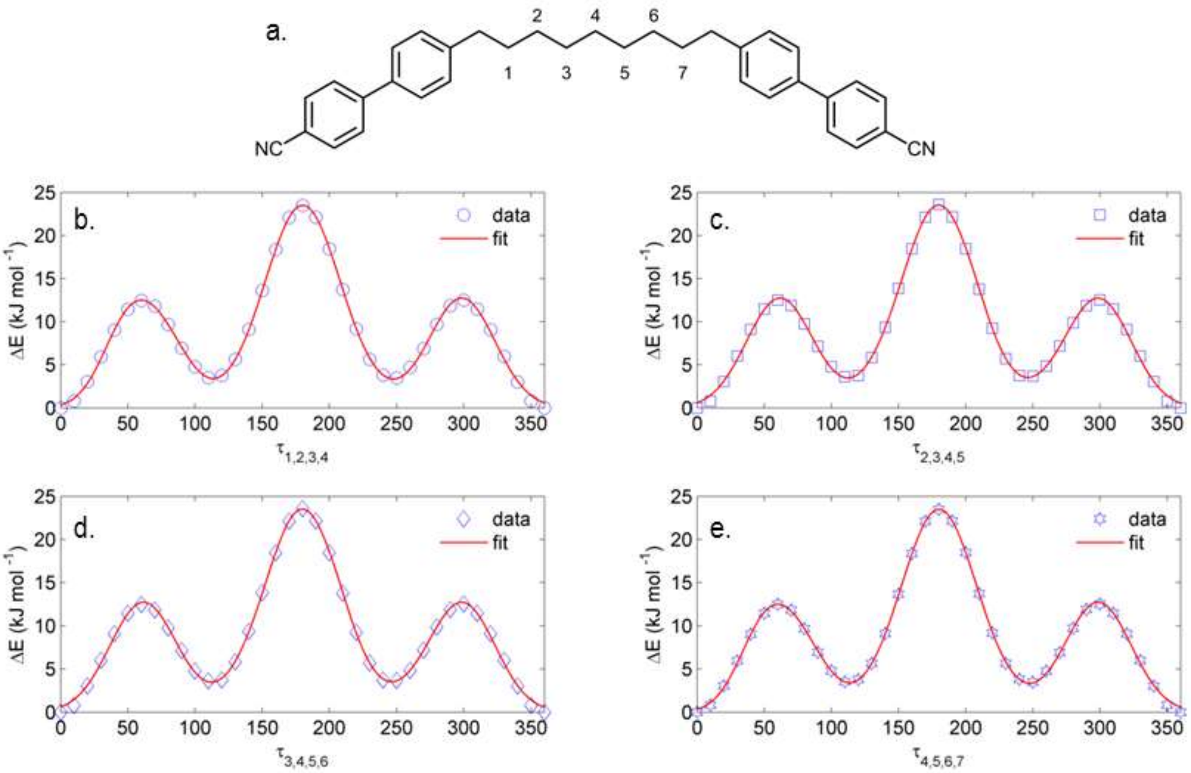

Figure SI30: The dihedral angles labelled as τ are defined based on the atomic numbering shown in (a). Plots of energy as a function of each of the four dihedral angles present in the flexible portion of the spacer of compound **1** at the B3LYP/6-31G(d) level of theory (36 x 10 ° steps) are given along with 6-term Gaussian fits (b – e). Energies are given in kJ mol^{-1} and are relative to the lowest energy conformer.

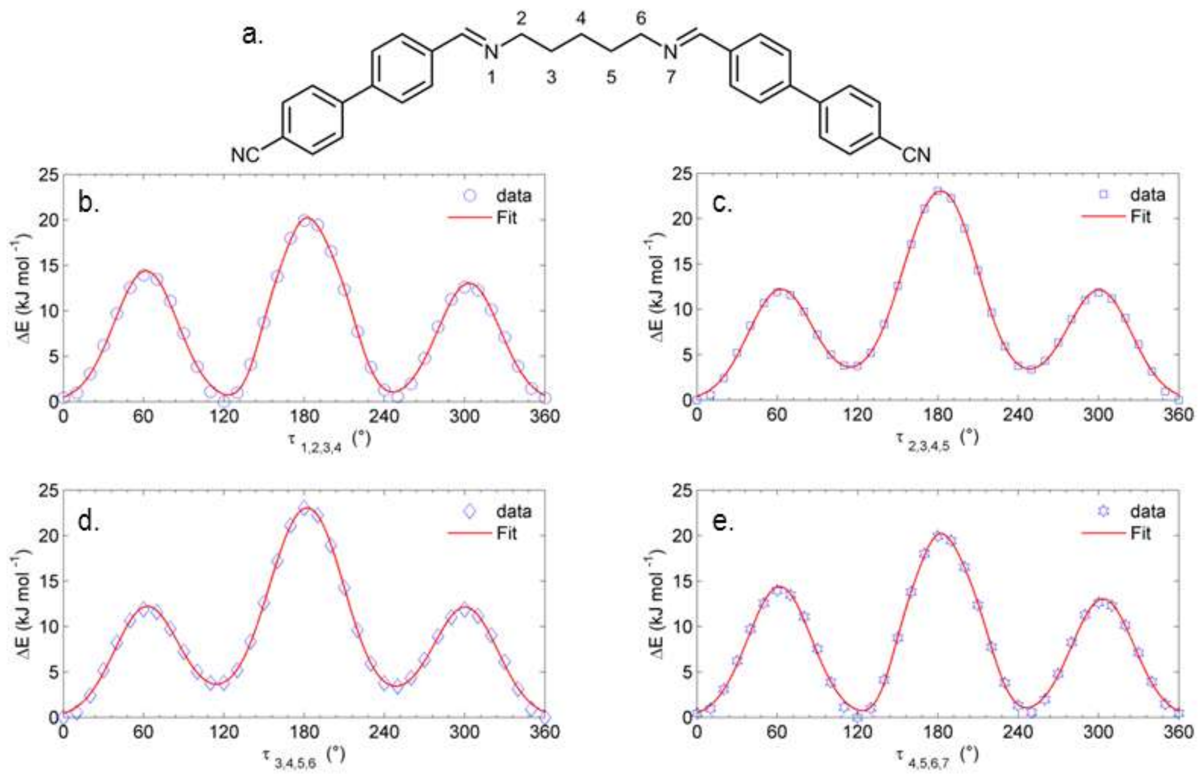

Figure SI31: The dihedral angles labelled as τ are defined based on the atomic numbering shown in (a). Plots of energy as a function of each of the four dihedral angles present in the flexible portion of the spacer of compound **2** at the B3LYP/6-31G(d) level of theory (36 x 10 ° steps) are given along with 6-term Gaussian fits (b – e). Energies are given in kJ mol^{-1} and are relative to the lowest energy conformer.

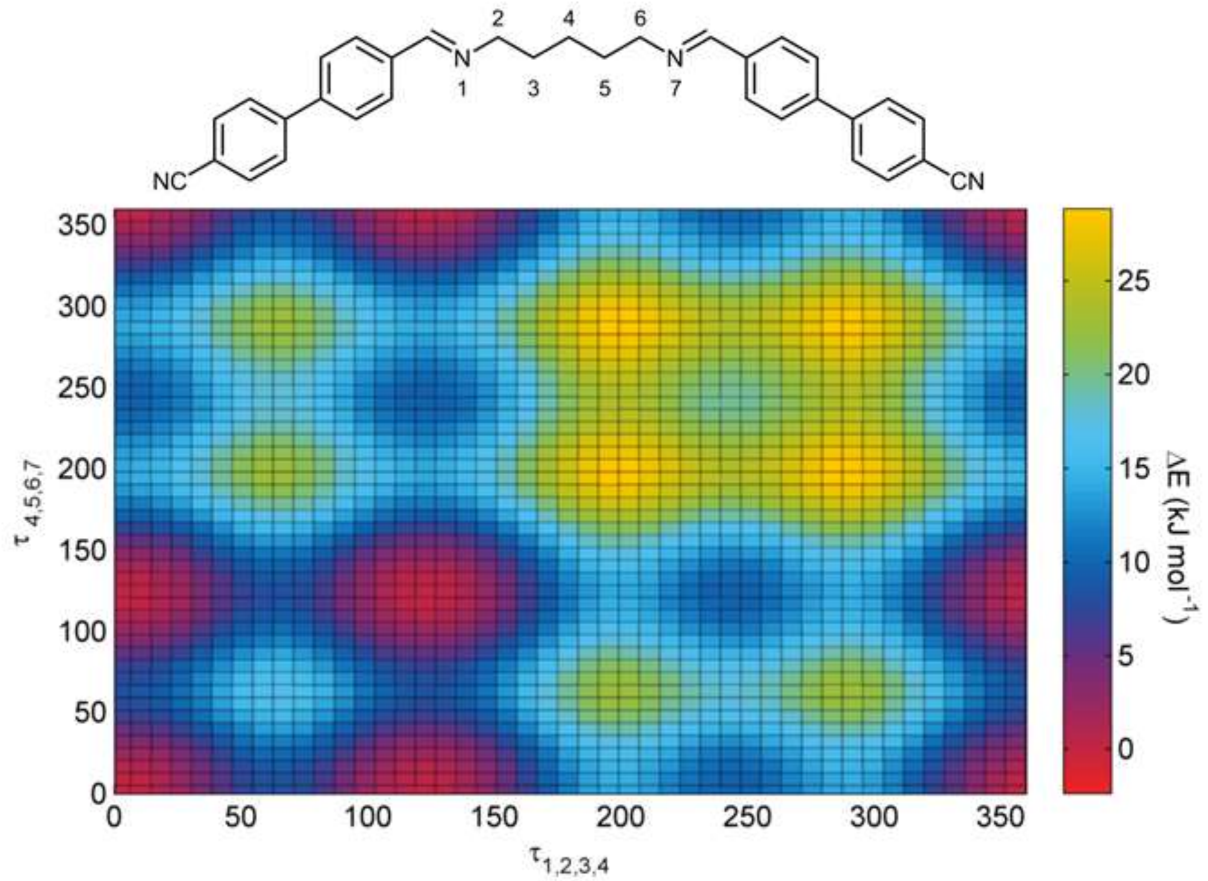

Figure SI32: Heatmap plot of energy (kJ mol^{-1}) relative to the lowest energy conformer as a function of the dihedral angles between atoms 1, 2, 3, 4 and 4, 5, 6, 7 of compound **2**. Calculations performed at the B3LYP/6-31G(d) level of theory (36 x 10 ° steps) using the MODREDUNDANT keyword.

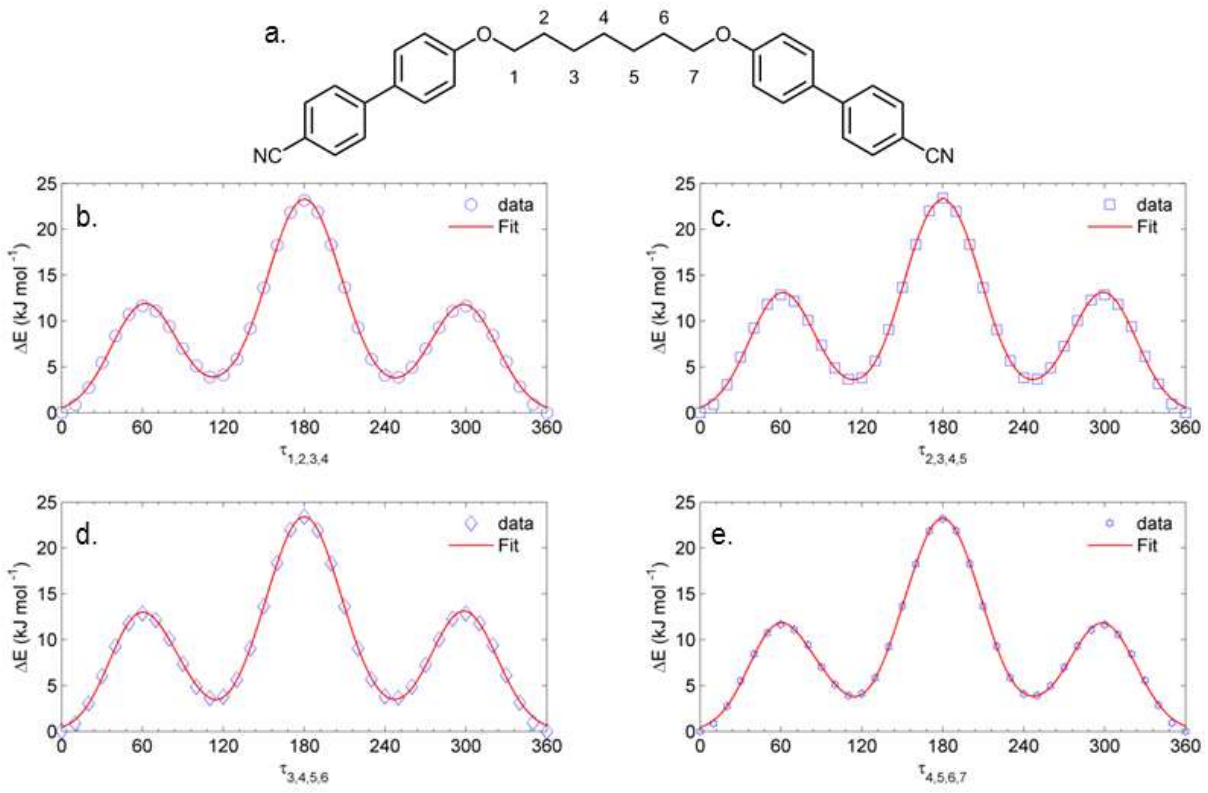

Figure SI33: The dihedral angles labelled as τ are defined based on the atomic numbering shown in (a). Plots of energy as a function of each of the four dihedral angles present in the flexible portion of the spacer of compound **3** at the B3LYP/6-31G(d) level of theory (36 x 10 ° steps) are given along with 6-term Gaussian fits (b – e). Energies are given in kJ mol^{-1} and are relative to the lowest energy conformer.

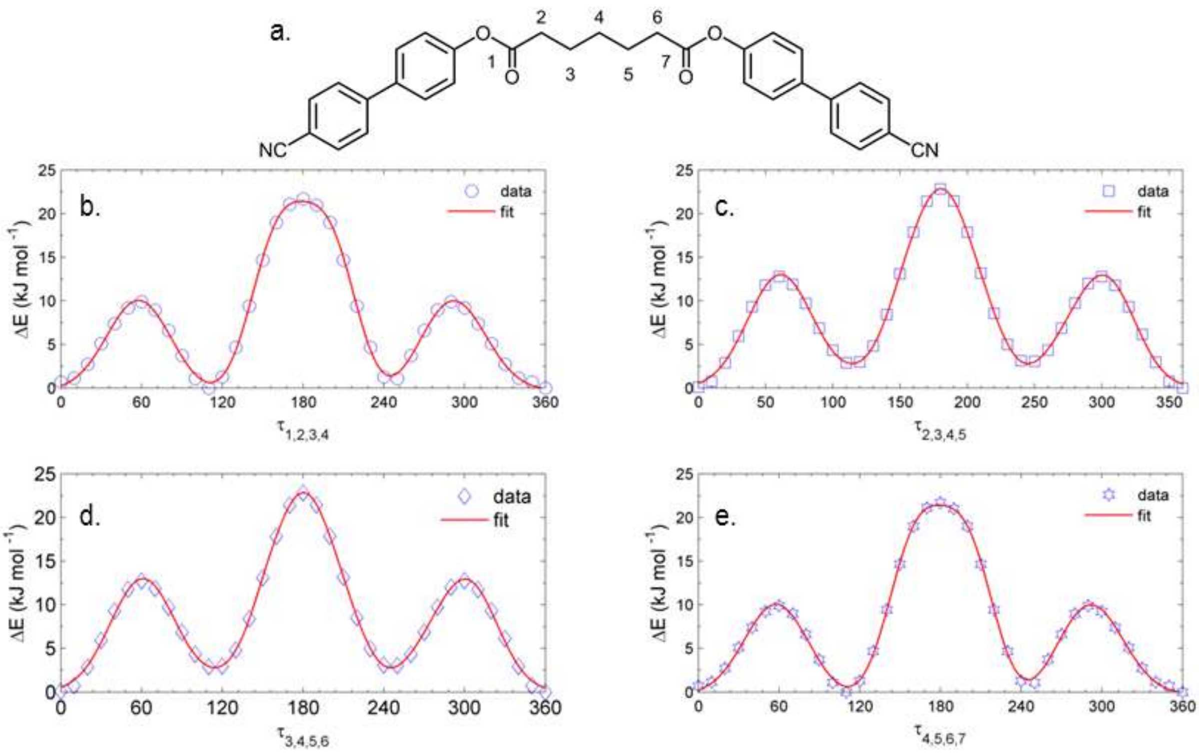

Figure SI34: The dihedral angles labelled as τ are defined based on the atomic numbering shown in (a). Plots of energy as a function of each of the four dihedral angles present in the flexible portion of the spacer of compound **4** at the B3LYP/6-31G(d) level of theory (36 x 10 ° steps) are given along with 6-term Gaussian fits (b – e). Energies are given in kJ mol^{-1} and are relative to the lowest energy conformer.

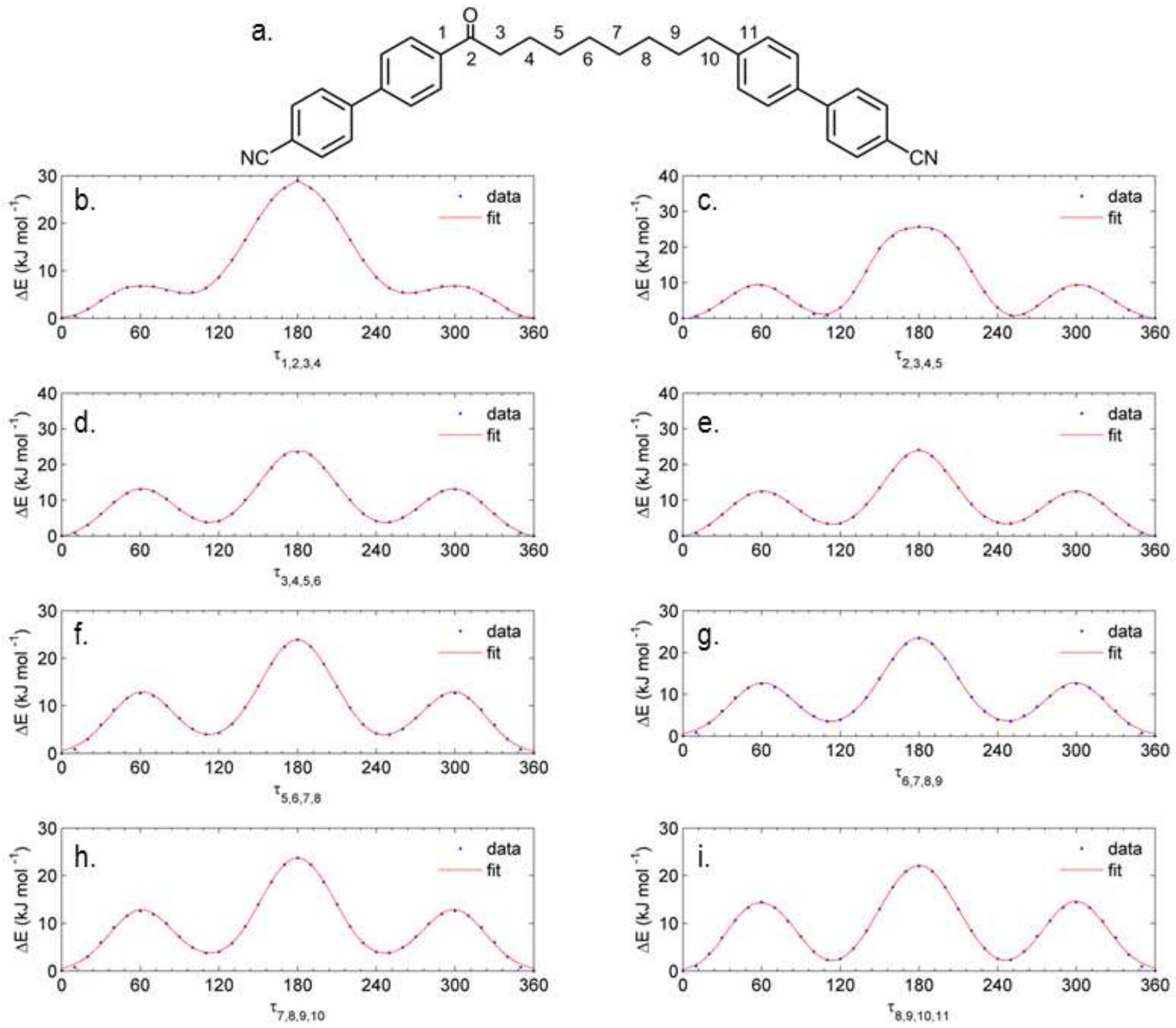

Figure SI35: The dihedral angles labelled as τ are defined based on the atomic numbering shown in (a). Plots of energy as a function of each of the eight dihedral angles present in the flexible portion of the spacer of compound **6** at the B3LYP/6-31G(d) level of theory (36 x 10 ° steps) are given along with 6-term Gaussian fits (b – i). Energies are given in kJ mol^{-1} and are relative to the lowest energy conformer.

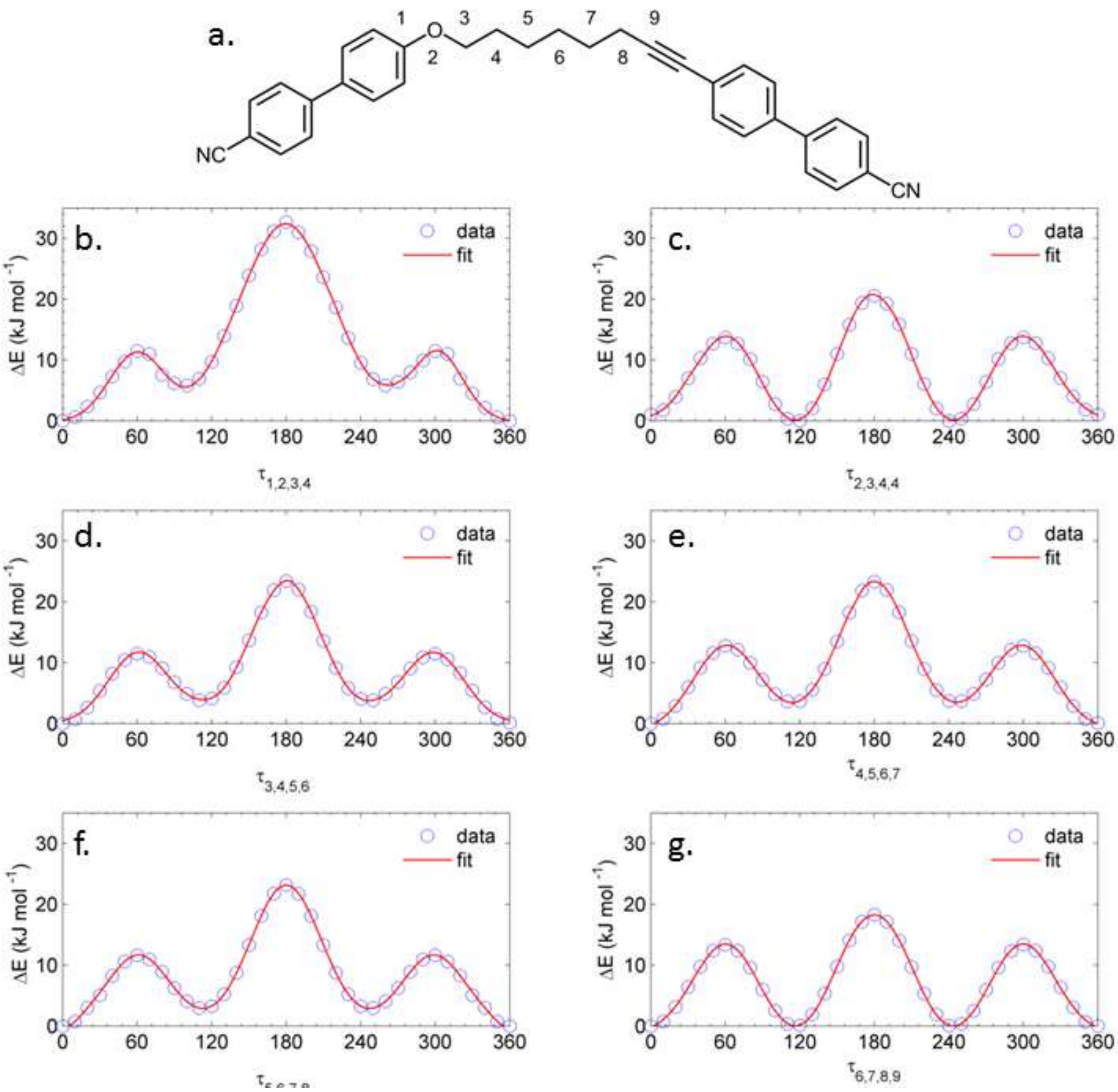

Figure SI36: The dihedral angles labelled as τ are defined based on the atomic numbering shown in (a). Plots of energy as a function of each of the eight dihedral angles present in the flexible portion of the spacer of compound 7**6** at the B3LYP/6-31G(d) level of theory (36 x 10 ° steps) are given along with 6-term Gaussian fits (b – g). Energies are given in kJ mol^{-1} and are relative to the lowest energy conformer.

1.4. Supplemental Phase Diagrams.

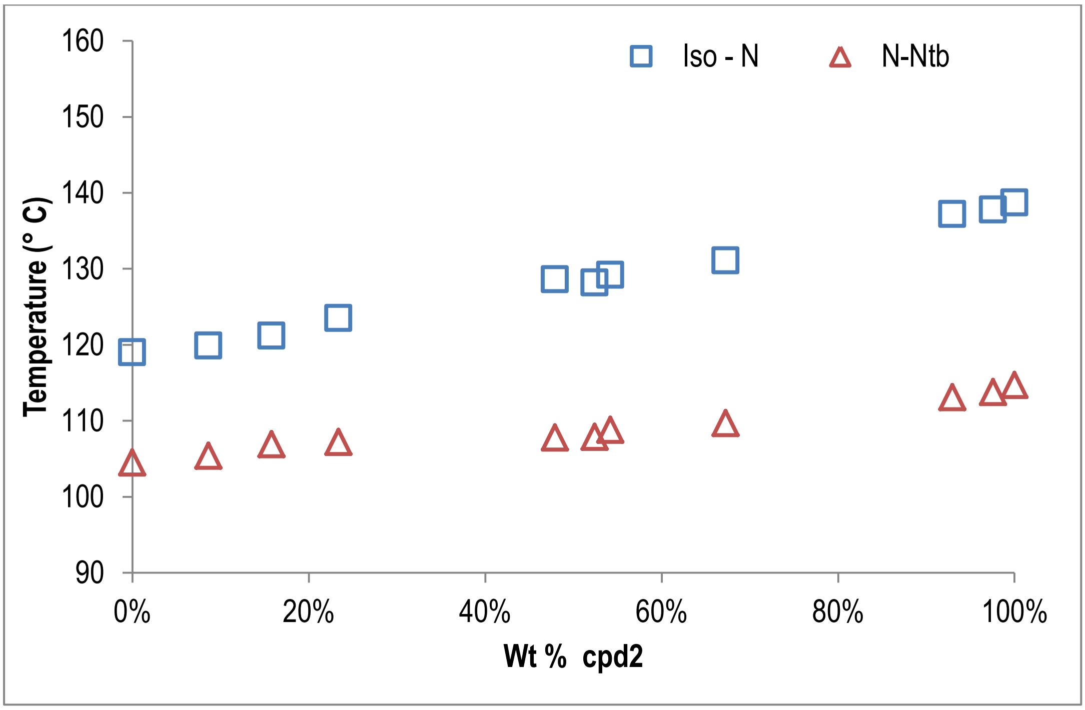

Figure SI37: Binary phase diagram for mixtures of compound **2** and CB9CB

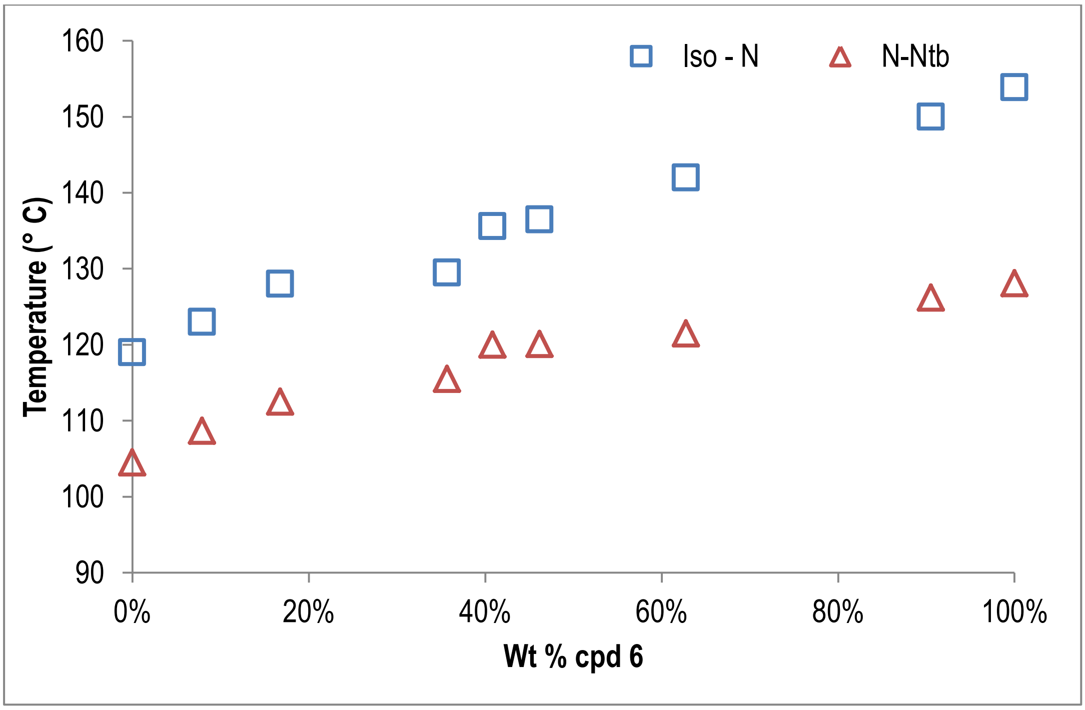

Figure SI38: Binary phase diagram for mixtures of compound **6** and CB9CB

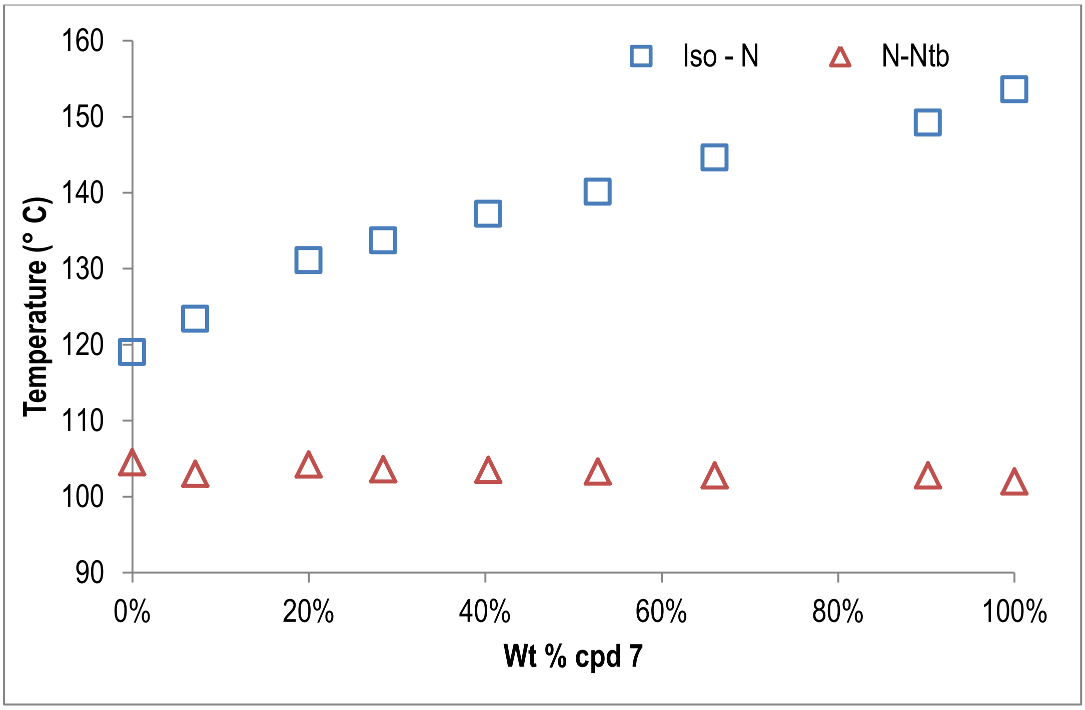

Figure SI39: Binary phase diagram for mixtures of compound **7** and CB9CB

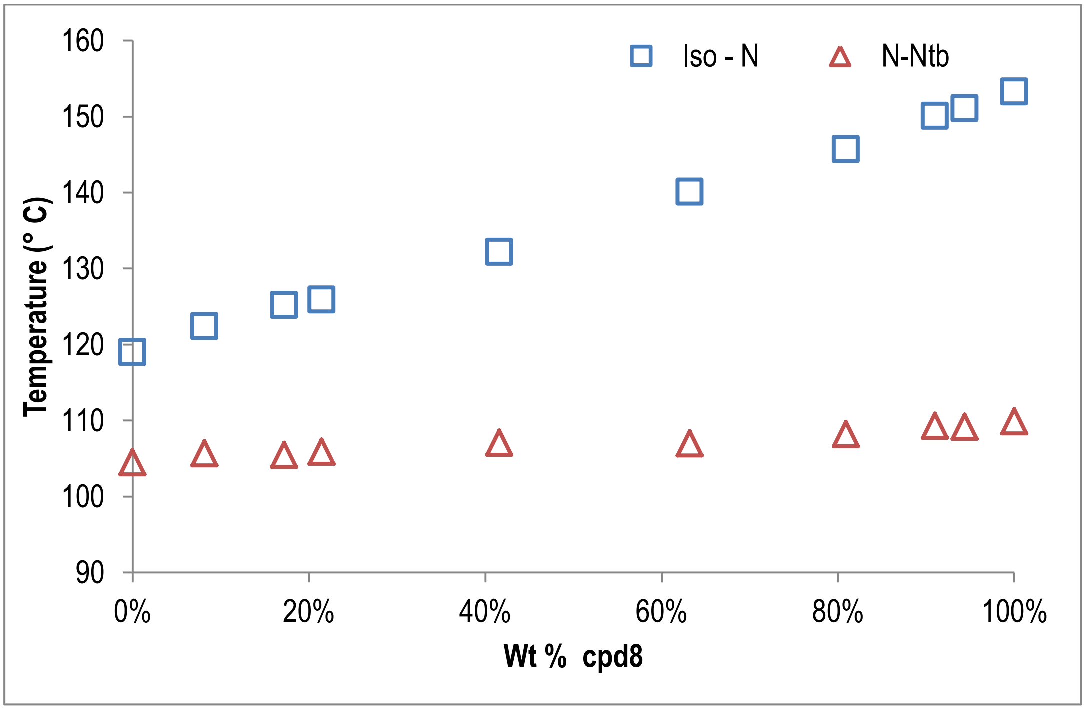

Figure SI40: Binary phase diagram for mixtures of compound **8** and CB9CB

References

1. R. J. Mandle, E. J. Davis, C.C.A. Voll, C.T. Archbold, J.W. Goodby and S.J. Cowling, *Liq. Cryst.,* **2015**, *42*, 688-703
2. J.W. Elmsley, G.R. Luckhurst, G.N.Shilstone and I. Sage, *Mol. Cryst. Liq. Cryst.,* **1984**, *102*, 223-233.
3. H. Kessler, H. Oschkinat, C. Griesinger and W. Bermel, J_{H-H}*. Magn. Reson.*, **1986**, *70*, 106
4. J. Stonehouse, P. Adell, J. Keeler and A.J. Shaka, J_{H-H}*ACS*, **1994**, *116*, 6037
5. K. Stott, J. Stonehouse, J. Keeler, T.L. Hwang and A.J. Shaka, J_{H-H}*ACS*, **1995**, *117*, 4199-4200
6. Gaussian 09, Revision D.01, M. J. Frisch, G. W. Trucks, H. B. Schlegel, G. E. Scuseria, M. A. Robb, J. R. Cheeseman, G. Scalmani, V. Barone, B. Mennucci, G. A. Petersson, H. Nakatsuji, M. Caricato, X. Li, H. P. Hratchian, A. F. Izmaylov, J. Bloino, G. Zheng, J. L. Sonnenberg, M. Hada, M. Ehara, K. Toyota, R. Fukuda, J. Hasegawa, M. Ishida, T. Nakajima, Y. Honda, O. Kitao, H. Nakai, T. Vreven, J. A. Montgomery, Jr., J. E. Peralta, F. Ogliaro, M. Bearpark, J. J. Heyd, E. Brothers, K. N. Kudin, V. N. Staroverov, R. Kobayashi, J. Normand, K. Raghavachari, A. Rendell, J. C. Burant, S. S. Iyengar, J. Tomasi, M. Cossi, N. Rega, J. M. Millam, M. Klene, J. E. Knox, J. B. Cross, V. Bakken, C. Adamo, J. Jaramillo, R. Gomperts, R. E. Stratmann, O. Yazyev, A. J. Austin, R. Cammi, C. Pomelli, J. W. Ochterski, R. L. Martin, K. Morokuma, V. G. Zakrzewski, G. A. Voth, P. Salvador, J. J. Dannenberg, S. Dapprich, A. D. Daniels, Ö. Farkas, J. B. Foresman, J. V. Ortiz, J. Cioslowski, and D. J. Fox, Gaussian, Inc., Wallingford CT, **2009**.
7. M. Tarini, P. Cignoni and C. Montani, *IEEE Transactions on Visualization and Computer Graphics*, **2006**, *12*, 1237-1244
